# Metabolomic and flavoromic insights into the effects of *Polygonatum kingianum* extract during black tea processing

**DOI:** 10.3389/fnut.2026.1882990

**Published:** 2026-07-08

**Authors:** Tianyu Wu, Xiaohua Wang, Xinghua Wang, Junjie He, Wenxia Yuan, Raoqiong Che, Qiaomei Wang, Zhie Zhu, Xinya Chen, Cangyi Zhao, Chunyan Zhao, Weihao Liu, Chun Wang, Zejun Wang, Hongmei An, Xiujuan Deng, Baijuan Wang

**Affiliations:** 1 College of Tea Science, Yunnan Agricultural University, Kunming, China; 2 Yunnan Organic Tea Industry Intelligent Engineering Research Center, Kunming, China; 3 Guoke Tea Yunnan Company Limited, Kunming, China

**Keywords:** black tea, key aroma components, medicinal and edible, metabolomics, *Polygonatum kingianum* extract

## Abstract

This study aims to elucidate the effects of the compounding treatment of *Polygonatum kingianum* extract (PKE) on the sensory quality, flavor chemical characteristics, and potential functional activities of control Dianhong black tea (CK). The results showed that *Polygonatum kingianum* black tea (PKB) had a darker and more lustrous appearance, a sweeter and more mellow taste, and a distinct floral and fruity aroma along with a refreshing herbal fragrance. The water extract and soluble sugar contents of PKB increased from 41.20 to 44.98% and from 7.03 to 10.91%, respectively (*p* < 0.01). LC–MS analysis identified the downregulation of bitter-associated flavonol glycosides, including quercetin 3-glucosyl-(1 → 2)-galactoside and quercetin 3-galactoside, together with the upregulation of sweet-related metabolites such as mannose, sorbose, and 6-phosphogluconic acid. Comprehensive two-dimensional gas chromatography–time-of-flight mass spectrometry (GC × GC–TOF-MS) analysis indicated that the volatile components of PKB, such as alcohols, ketones, and esters, significantly increased. The key aroma compounds cooperatively enhanced the aroma richness. Based on variable importance in projection (VIP) and Relative Odor Activity Value (ROAV) screening, 7 key aroma components were identified; among them, 2-methylbutanal (ROAV = 100) was the core component for fruity and sweet scents. These changes were associated with alterations in flavonoid, amino acid, carbohydrate, and aroma-related metabolic pathways. Overall, PKE treatment significantly improved the sensory quality and flavor characteristics of Dianhong black tea.

## 1 Introduction

Black tea is one of the most widely consumed teas worldwide and is classified as a fully fermented type. Its traditional manufacturing process consists of withering, rolling, fermentation, and drying, during which enzymatic oxidation converts leaf catechins into theaflavins (TFs) and thearubigins (TRs). Fresh tea leaves inherently contain a range of volatile compounds, but these are substantially reshaped during black tea processing. Through withering, rolling, fermentation, and drying, precursor substances are transformed, and new volatile aromatic compounds, such as linalool and geraniol, are generated, thereby contributing to the floral and sweet aroma characteristics of black tea. Meanwhile, coordinated changes in non-volatile components are also responsible for the characteristic reddish liquor and sweet-mellow taste of black tea (1). In recent years, increasing consumer demand for enhanced health benefits and diversified flavor profiles has driven the development of innovative processing strategies aimed at conferring black tea with novel functional attributes and distinctive sensory qualities (2).

*Polygonatum kingianum* Coll. et Hemsl., a distinctive medicinal and edible plant native to Yunnan Province, China, possesses rhizomes rich in polysaccharides, saponins, flavonoids, and polyphenolic compounds, which have been associated with various bioactive properties (3–5). Owing to its unique flavor and nutritional characteristics, *Polygonatum kingianum* has been increasingly utilized in functional foods, beverages, and tea-like products. However, current commercial products are mainly limited to simple tea–*Polygonatum kingianum* blends or single-ingredient extracts, and studies investigating its application in tea processing remain scarce.

In recent years, the incorporation of medicinal and edible plants into tea products has attracted increasing attention because of their potential to improve sensory quality and diversify product characteristics. Various medicinal and edible plants, including goji berry (*Lycium barbarum*) and *Eucommia ulmoides*, have been developed into tea beverages or tea-like products owing to their distinctive flavor profiles and phytochemical compositions (6–8). Nevertheless, compared with these medicinal and edible plants, the influence of *P. kingianum* on flavor formation, aroma development, and metabolite composition during black tea processing has not been systematically investigated. Therefore, elucidating the changes in sensory quality and flavor-related metabolites induced by *P. kingianum* during Dianhong black tea processing is important for developing novel tea products and understanding their flavor formation mechanisms.

To address the current knowledge gap, this study investigated the effects of *P. kingianum* extract (PKE) on the sensory quality, flavor characteristics, and metabolite profiles of Dianhong black tea prepared from ‘Fengqing Large Leaf’, a representative Yunnan large-leaf tea cultivar belonging to *Camellia sinensis* var. assamica (9). Particular attention was paid to the changes in flavor-related metabolites and aroma-active compounds associated with PKE treatment during black tea processing. The findings provide new insights into the flavor formation mechanisms of medicinal–edible homologous tea products and offer a scientific basis for the development of novel *P. kingianum*-based black tea beverages.

## 2 Materials and methods

### 2.1 Fresh tea leaves and *Polygonatum kingianum*

The fresh tea leaves were sourced from Fanjiawo, Fengqing County, Lincang City, Yunnan Province (99°52′E; 24°37′N), with an elevation of 1,954.1 m. The tea tree variety is the ‘Fengqing large-leaf variety,’ and the fresh leaf grade consists of one bud with two to three leaves. The *P. kingianum* material used was processed through the traditional “Nine Cycles of Steaming and Sun-Drying” method and sourced from Pu′er Liangbao Biotechnology Co., Ltd.

### 2.2 Production process of PKB

**Figure 1:**
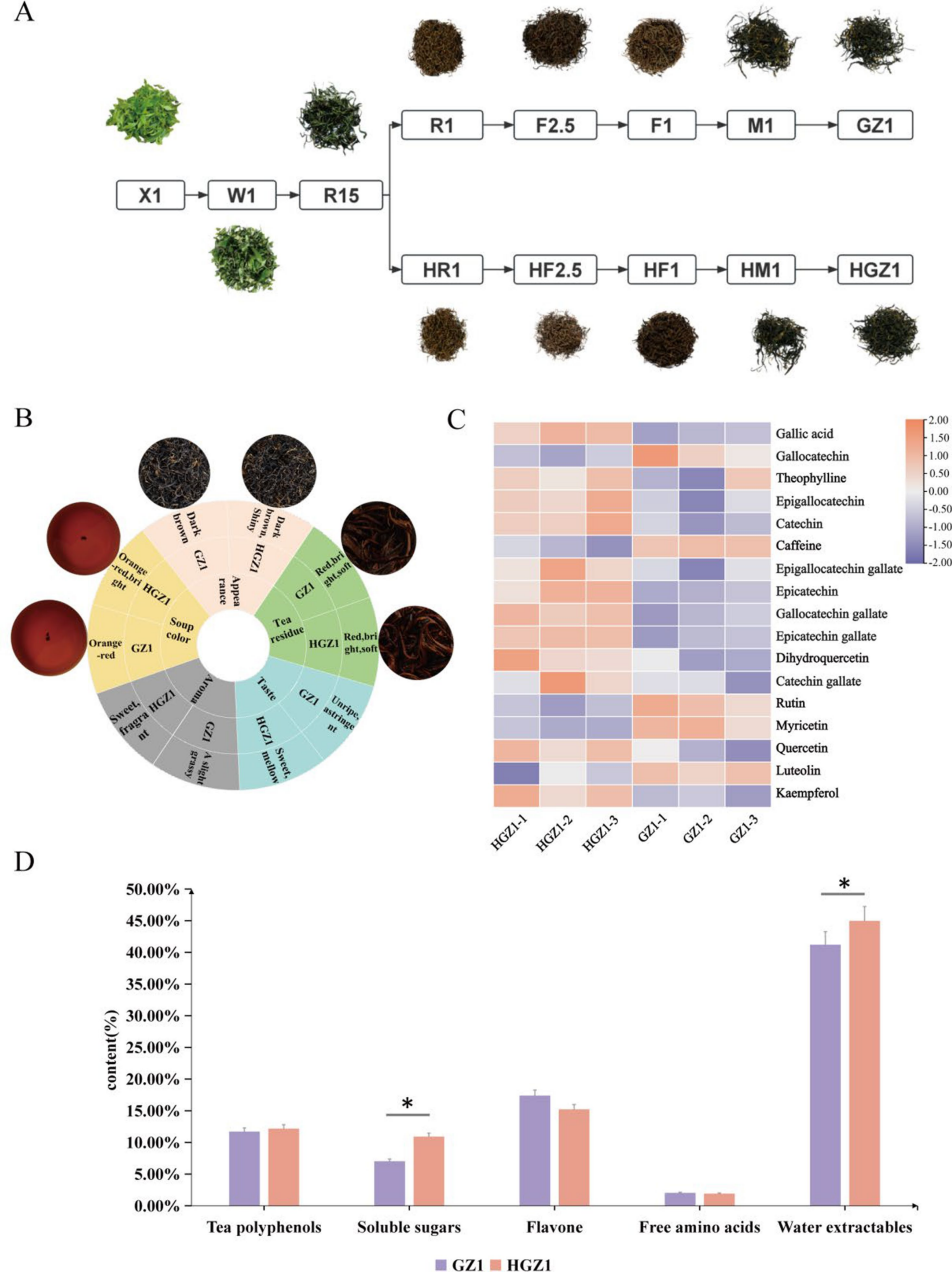
Changes in the sensory quality and characteristics of HGZ1 and GZ1. **(A)** Process samples of HGZ1 and GZ1 during production. **(B)** Images and evaluation results of HGZ1 and GZ1 sensory evaluation. **(C)** Results of quantitative analysis by high-performance liquid chromatography (HPLC) of HGZ1 and GZ1. **(D)** Basic physical and chemical results of HGZ1 and GZ1 leaves.

Fresh leaves of ‘Fengqing Large Leaf’ (*C. sinensis* var. *assamica*), a representative Yunnan large-leaf tea cultivar., were used as raw materials. The traditional black tea manufacturing process consisted of withering, rolling, fermentation, and drying. As shown in Figure 1A, the PKB manufacturing process differed from that of the control black tea in that *P. kingianum* extract (PKE) was added after 15 min of rolling.

Fresh leaves were first subjected to withering using a combination of hot and cold air. Hot-air withering was conducted for 1 h 40 min, followed by cold-air withering for 2 h 30 min. The withered leaves were then rolled using a 6CR-55 tea rolling machine. After 15 min of rolling, PKE was added to the treatment group. Rolling was subsequently carried out under a stepwise regime consisting of light rolling for 45 min, medium rolling for 60 min, and heavy rolling for 30 min, resulting in a total rolling time of 2.5 h. Fermentation was performed at 29 ± 1 °C and 90% relative humidity for 5 h. After fermentation, tea samples were dried in two stages: primary drying at 110 °C and final drying at 90 °C.

The sampled stages were defined as follows: fresh leaves (X1), withering (W1), rolling for 15 min (R15), end of rolling at 2.5 h for CK (R1), end of rolling at 2.5 h for PKB (HR1), fermentation for 2.5 h for CK (F25), fermentation for 2.5 h for PKB (HF25), end of fermentation at 5 h for CK (F1), end of fermentation at 5 h for PKB (HF1), end of primary drying for CK (M1), end of primary drying for PKB (HM1), end of final drying for CK (GZ1), and end of final drying for PKB (HGZ1).

For clarity, all tea samples were classified into two groups: PKB and control Dianhong black tea (CK). The PKB group comprised X1, W1, R15, HR1, HF25, HF1, HM1, and HGZ1, whereas the CK group comprised X1, W1, R15, R1, F25, F1, M1, and GZ1.

PKE: Rhizomes of *P. kingianum*, steamed and sun-dried nine cycles, were selected and soaked in purified water at 90 °C for 1 h to obtain Extract 1. Purified water was then added again to the *P. kingianum* and soaked at 90 °C for 1 h to obtain Extract 2. Finally, purified water was added once more and soaked at 90 °C for 1 h to obtain Extract 3. The three extracts were thoroughly mixed and concentrated under reduced pressure to produce the PKE.

### 2.3 Sensory evaluation methods for tea

According to GB/T 23776-2018, Sensory Evaluation Method for Tea, sensory evaluation of the tea samples was carried out by five trained professional evaluators based on appearance, aroma, taste, liquor color, and infused leaves. Each attribute was scored and described according to GB/T 14487-2017, sensory evaluation terminology for tea.

### 2.4 Physicochemical analysis of tea

The water-extractable content in tea is measured according to GB/T 8305-2013. The total polyphenol content in tea infusion is determined using the Folin–Ciocalteu method, following GB/T 8313-2018. Flavonoid content is measured by the aluminum chloride colorimetric method (10). Soluble sugar content is determined using the anthrone-sulfuric acid colorimetric method (11). Catechin, flavonoid, amino acid components, and caffeine content are analyzed by high-performance liquid chromatography (HPLC), as described in related studies (12).

### 2.5 Metabolomics analysis

#### 2.5.1 Non-volatile metabolite detection

An appropriate amount of sample was accurately weighed into a 2 mL centrifuge tube, 600 μL of methanol containing 2-amino-3-(2-chloro-phenyl)-propionic acid (4 ppm) was added, and the mixture was vortex-mixed for 30 s. Subsequently, steel balls were introduced, and the tube was subjected to tissue grinding at 55 Hz for 60 s, followed by ultrasonic extraction at room temperature for 15 min. The resulting suspension was then centrifuged at 12,000 rpm and 4 °C for 10 min, and the supernatant was filtered through a 0.22 μm membrane and transferred to a detection vial for LC–MS analysis.

The LC analysis was performed on a Vanquish UHPLC System (Thermo Fisher Scientific, USA). Chromatography was carried out with an ACQUITY UPLC ® HSS T3 (2.1 × 100 mm, 1.8 μm) (Waters, Milford, MA, USA). The column was maintained at 40 °C. The flow rate and injection volume were set at 0.3 mL/min and 2 μL, respectively. For LC-ESI (+)-MS analysis, the mobile phases consisted of (B2) 0.1% formic acid in acetonitrile (v/v) and (A2) 0.1% formic acid in water (v/v). Separation was conducted under the following gradient: 0~1 min, 10% B2; 1~5 min, 10%~98% B2; 5~6.5 min, 98% B2; 6.5~6.6 min, 98%~10% B2; 6.6~8 min, 10% B2. For LC-ESI (−)-MS analysis, the analytes were carried out with (B3) acetonitrile and (A3) ammonium formate (5 mM). Separation was conducted under the following gradient: 0~1 min, 10% B3; 1~5 min, 10%~98% B3; 5~6.5 min, 98% B3; 6.5~6.6 min, 98%~10% B3; 6.6~8 min, 10% B3.

#### 2.5.2 Mass spectrum conditions

Mass spectrometric detection of metabolites was performed on a Q Exactive Focus (Thermo Fisher Scientific, USA) with an ESI ion source. Simultaneous MS1 and MS/MS (Full MS-ddMS2 mode, data-dependent MS/MS) acquisition was used. The parameters were as follows: sheath gas pressure, 40 arb; aux gas flow, 10 arb; spray voltage, 3.50 kV and −2.50 kV for ESI (+) and ESI (−), respectively; capillary temperature, 325 °C; MS1 range, *m*/*z* 100–1,000; MS1 resolving power, 70000 FWHM; number of data-dependent scans per cycle, 3; MS/MS resolving power, 17500 FWHM; normalized collision energy, 30 eV; dynamic exclusion time, automatic (13).

### 2.6 Aroma analysis

#### 2.6.1 Internal standard solution preparation

1 mg/L n-hexyl-d13 alcohol stock solution was prepared in 50% (v/v) ethanol, whereas an equivalent 1 mg L^−1^ n-alkane stock solution was prepared in n-hexane; both solutions were subsequently stored at 4 °C to ensure long-term stability.

#### 2.6.2 Sample preparation

An aliquot of each sample was transferred into a 20 mL headspace vial, treated with 4 mL saturated NaCl solution, and spiked with 10 μL of internal standard (ISTD) solution. After equilibrating the vial at 80 °C for 10 min, a conditioned SPME fiber was introduced into the headspace at the same temperature for 25 min. Prior to extraction, the fiber had been thermally conditioned at 270 °C for 10 min; following extraction, it was desorbed in the GC injector at 250 °C for 5 min and subsequently re-conditioned for 10 min at 270 °C. To establish retention indices, 10 μL of an n-alkane standard was transferred into a separate 20 mL headspace vial and subjected to identical incubation, extraction, and desorption conditions.

#### 2.6.3 Identification and sensory annotation of volatile compounds

Volatile compounds were identified using Chroma TOF software based on their retention times and retention indices, with reference standards obtained from the NIST 2020 library. The sensory flavor characteristics of all tea samples were further evaluated by comparison with the Odor Database and FlavorDB (14).

#### 2.6.4 Relative Odor Activity Value (ROAV) analysis

The contribution of individual volatile compounds to the overall aroma profile was evaluated using the Relative Odor Activity Value (ROAV). ROAV was calculated according to the following equation:


$${\text{ROAV}}_{i}\approx 100\times \frac{C_{\mathrm{ri}}}{C_{r\;\text{stan}}}\times \frac{T_{\text{stan}}}{T_{i}}$$

Where $C_{r\;\text{stan}}$ is the relative percentage content (%) of the compound with the greatest contribution to the overall aroma profile; $T_{\text{stan}}$ is the odor threshold (μg/kg) of the compound with the greatest contribution to the overall aroma profile; $C_{\mathrm{ri}}$ is the relative percentage content (%) of the target compound; and $T_{i}$ is the odor threshold (μg/kg) of the target compound.

### 2.7 Statistical analysis

Physicochemical analysis, metabolomics determination, flavoromics analysis, and electronic nose measurements were carried out in three independent experiments (*n* = 3). All data are expressed as the mean ± standard deviation (SD). One-way analysis of variance (ANOVA) was performed using SPSS 27.0. Multivariate statistical analyses, including principal component analysis (PCA), hierarchical clustering analysis (HCA), orthogonal partial least squares discriminant analysis (OPLS-DA), heatmaps, and Venn diagrams, were performed using the Majorbio Cloud Platform and R (v4.2.2). The reliability of the OPLS-DA model was evaluated using a 100-permutation test. Model fitness and predictive ability were assessed based on *R*^2^*Y* and *Q*^2^ values. A model was considered reliable when the permutation test showed no obvious overfitting and the permuted *Q*^2^ values were lower than the original *Q*^2^ value. Differential metabolites were screened using a combination of OPLS-DA variable importance in projection (VIP) values, Student’s *t*-test with Benjamini–Hochberg false discovery rate (FDR) correction, and fold change (FC) analysis. Metabolites meeting the criteria of VIP > 1.0, FDR < 0.05, and FC ≥ 1.5 or ≤ 0.67 were defined as significant differential metabolites. Volcano plots and heatmaps were used to visualize the distribution and abundance patterns of differential metabolites. For electronic nose analysis, OPLS-DA was performed using processed sensor response data. The VIP values of individual sensors were calculated to identify the most important sensors contributing to sample discrimination, and radar plots were subsequently generated to visualize their relative contributions to aroma characteristics. Figures were generated using Origin 2024.

## 3 Results and discussion

### 3.1 Changes in the sensory quality and characteristics of PKB

Compared to GZ1, HGZ1 exhibits a darker and more lustrous appearance, a sweeter and more mellow taste, and a richer, longer-lasting aroma characterized by fresh sweetness with herbal notes (Figure 1B). Physicochemical analyses (Figure 1D) reveal that HGZ1 contained higher tea polyphenols (12.18% vs. 11.70%) and substantially elevated soluble sugars (10.91% vs. 7.03%), while flavonoids were slightly reduced (15.22% vs. 17.40%). Free amino acid levels showed minimal difference (HGZ1: 2.03%; GZ1: 1.92%). Notably, HGZ1 exhibited significantly higher water-extractable substances (44.98% vs. 41.20%). PKE treatment significantly increased soluble sugar and water-extractable substance content (*p* < 0.01). The markedly soluble sugar content was significantly higher than that of the CK, which may explain why HGZ1 exhibits a noticeably sweeter and mellower taste compared to GZ1. This difference could be attributed to the high content of monosaccharides and disaccharides in PKE (15). HPLC quantitative analysis further revealed distinct compositional profiles (Figure 1C): HGZ1 contained elevated levels of EGCG, EC, GCG, ECG, GA, and quercetin, whereas GZ1 showed higher concentrations of caffeine, rutin, and luteolin. These differential compound distributions may drive variations in antioxidant activity and sensory characteristics between groups.

### 3.2 Main changes in PKB non-volatile metabolites

**Figure 2:**
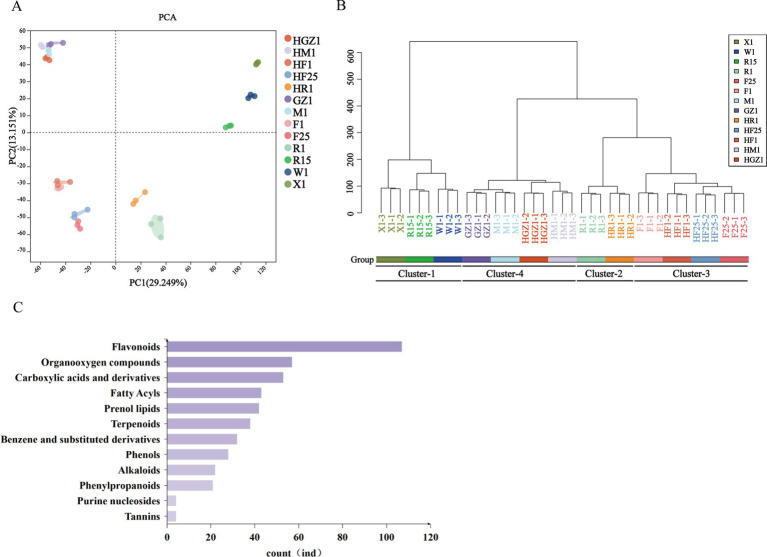
Overview of non-volatile metabolite profiles during PKB and CK processing. **(A)** Principal component analysis (PCA) score plot based on the combined positive and negative ion datasets. **(B)** Hierarchical cluster analysis (HCA) of samples based on the combined positive and negative ion datasets. Each node represents a sample, and node size reflects the number of samples within the corresponding cluster. **(C)** Classification of the 728 identified non-volatile metabolites.

A total of 728 non-volatile metabolites were identified, including 107 flavonoids, 57 oxidized azo compounds, 53 carboxylic acids and their derivatives, 43 fatty acids and their derivatives, 42 isoprenoid lipids, 38 terpenes, 32 benzenes and substituted derivatives, 28 phenolics, 22 alkaloids, 21 phenylpropanoids, 4 purine nucleosides, 4 tannins, and 277 other metabolites (Figure 2C). To further elucidate the changes in non-volatile components throughout the entire processing of PKB and the CK, we performed principal component analysis (PCA) on the non-volatile metabolites. The PCA score plots revealed distinct grouping patterns between PKB and the CK, as well as among different processing stages of PKB, indicating significant changes in non-volatile compounds during black tea processing. Clear sample separation was observed under the combined positive and negative ion modes. PC1 and PC2 explained 29.25 and 13.15% of the variance under the combined ion mode (Figure 2A), reflecting the separation trends of metabolic components between PKB and CK at different processing stages. Reflecting the separation trends of metabolic components in PKB across different processing stages.

Hierarchical cluster analysis (HCA) of metabolites detected in positive and negative ion mode (Figure 2B) revealed four distinct clusters corresponding to key processing stages: Cluster-1 (pre-rolling: X1, W1, R15), Cluster-2 (rolling completion: HR1, R1), Cluster-3 (fermentation: pre-fermentation HF25/F25; post-fermentation HF1/F1), and Cluster-4 (drying: primary HM1/M1; final HGZ1/GZ1). These findings suggest that processing stages significantly impact black tea quality by generating unique metabolic profiles. In Cluster-4, HGZ1 and GZ1 exhibited distinct subgroups under identical processing conditions, indicating that PKE addition significantly alters metabolite composition.

**Figure 3:**
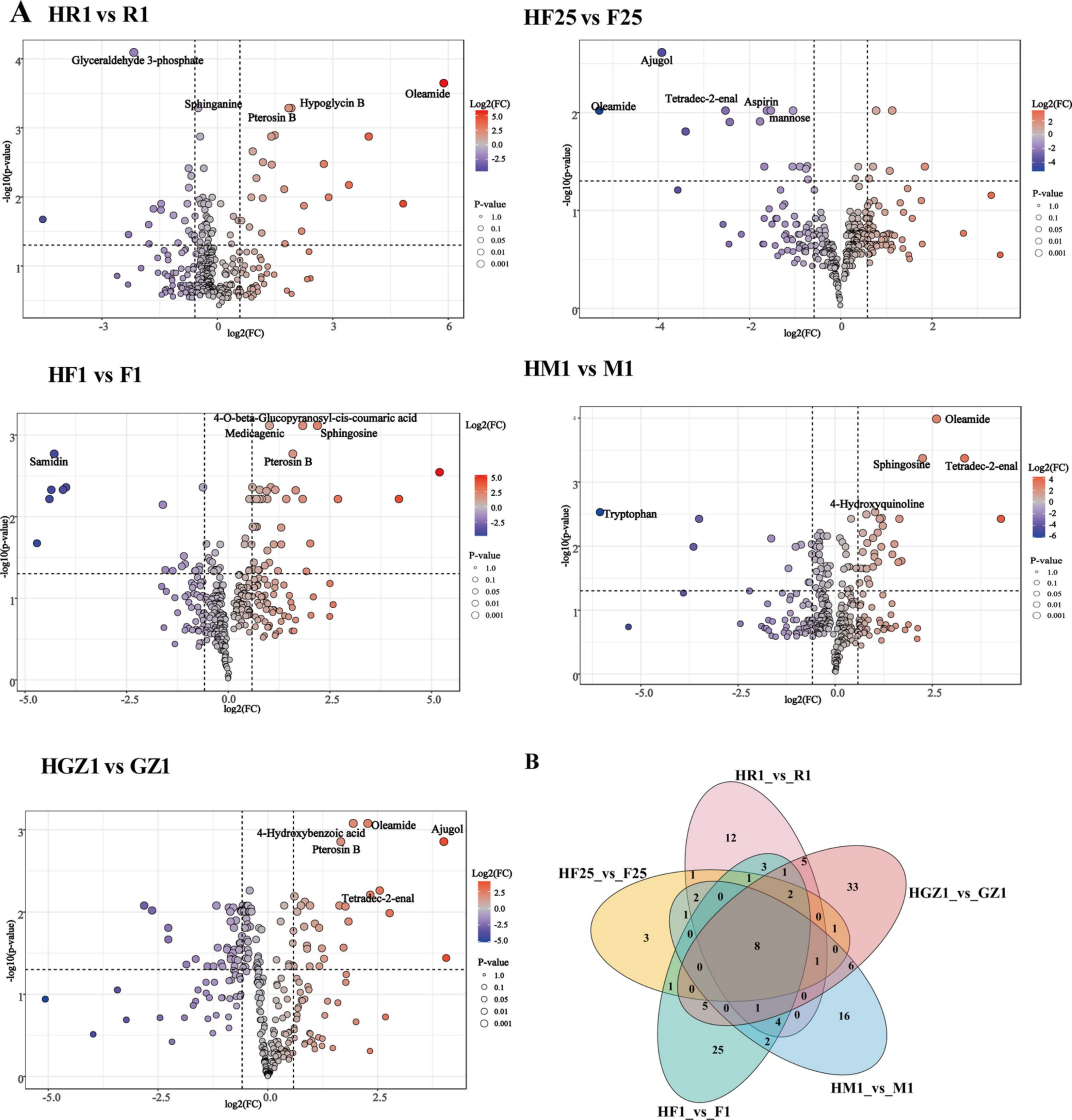
Major changes in the non-volatile metabolites of PKB. **(A)** Volcano plot showing the differences in metabolites between PKB and CK at the same stage of processing. **(B)** A Venn diagram showing the metabolites in common and the differences between PKB and CK at different stages of processing.

**Figure 4:**
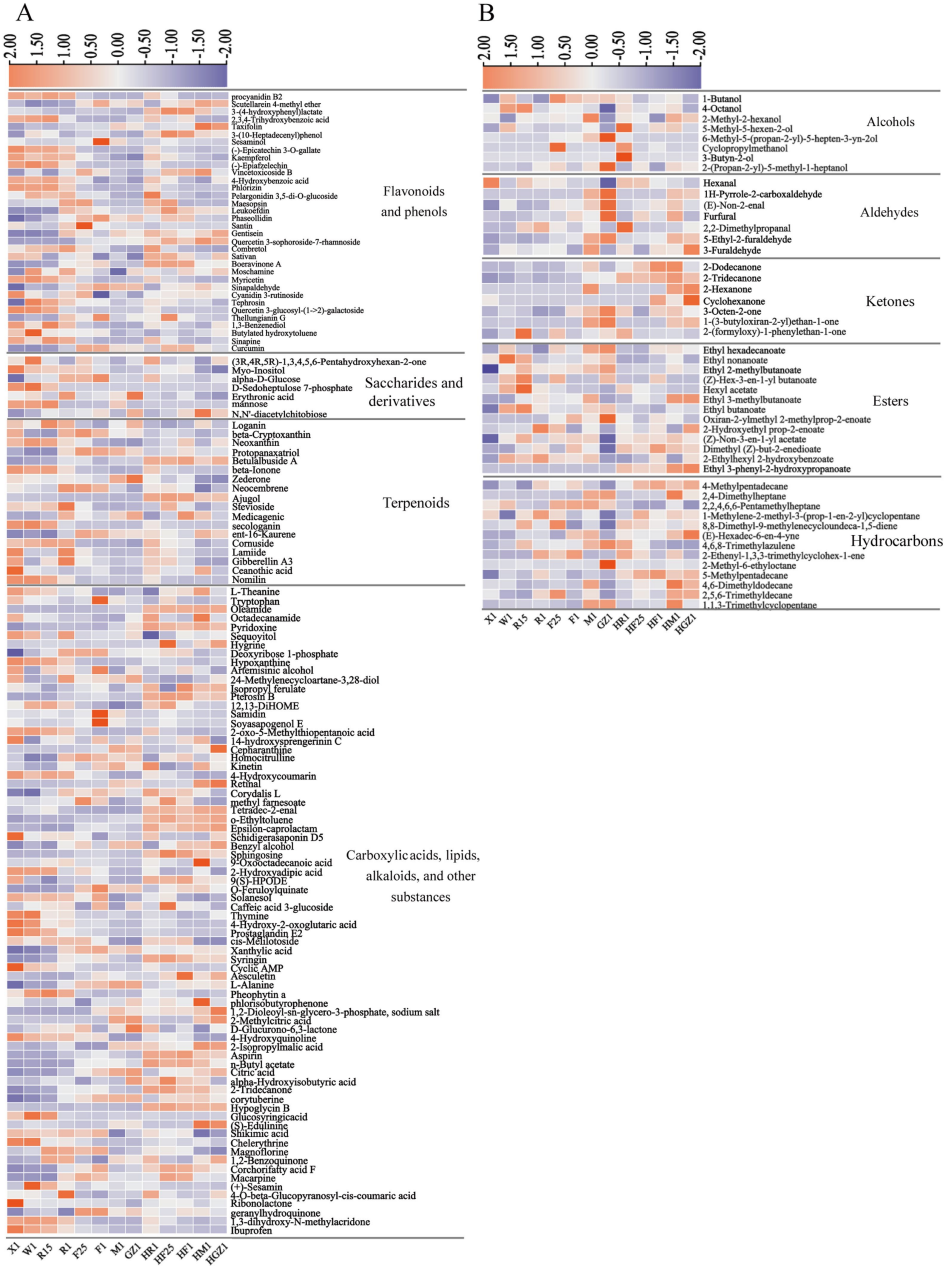
Heat maps of non-volatile and volatile metabolites in PKB. **(A)** Heat map of non-volatile metabolites. **(B)** Heat map of volatile metabolites.

Differential metabolites between adjacent processing stages were screened using one-way ANOVA combined with the criteria VIP > 1.0, fold change (FC) ≥ 1.5 or ≤ 0.67, and FDR < 0.05, and visualized by volcano plots (Figure 3A). Significant metabolic divergence between PKB and CK was observed across all processing stages. Among the comparison groups, HGZ1_vs_GZ1 exhibited the largest number of differential metabolites (27 up-regulated and 36 down-regulated), followed by HF1_vs_F1 (36 up-regulated and 17 down-regulated), HM1_vs_M1 (30 up-regulated and 11 down-regulated), HR1_vs_R1 (23 up-regulated and 18 down-regulated), and HF25_vs_F25 (6 up-regulated and 15 down-regulated). After merging and removing redundancy among differential non-volatile metabolites identified from HR1_vs_R1, HF25_vs_F25, HF1_vs_F1, HM1_vs_M1, and HGZ1_vs_GZ1, a total of 133 unique differential metabolites were obtained (Figure 4A, Supplementary Table S1).

Notably, large numbers of differential metabolites were detected during both fermentation and drying stages, indicating that these two processing steps represent critical windows for metabolite remodeling during PKB manufacture. In particular, substantially more metabolites were up-regulated in the HGZ1_vs_GZ1 and HM1_vs_M1 comparison groups, suggesting that drying plays a decisive role in promoting the accumulation of compounds associated with PKB quality formation. In contrast, the rolling stage contributed relatively less to the overall metabolic divergence between PKB and CK.

Venn diagram analysis further revealed eight common differential metabolites shared among HR1_vs_R1, HF25_vs_F25, HF1_vs_F1, HM1_vs_M1, and HGZ1_vs_GZ1, indicating that these metabolites were continuously influenced by PKE supplementation throughout processing. These metabolites included 9(S)-HPODE, Ajugol, Pterosin B, Sphingosine, and Tetradec-2-enal (Figure 3B). Several of these metabolites began to accumulate from the rolling stage onward and remained significantly enriched in HGZ1 compared with GZ1, suggesting sustained promotion of their biosynthesis during PBK processing following PKE addition (Figure 4A, Supplementary Table S1).

The 133 metabolites were classified into 18 chemical categories, including lipids and lipid-like molecules (7), flavonoids (19), phenolics (15), carboxylic acids and derivatives (12), phenylpropanoids (9), alkaloids (8), terpenoids (18), saccharides and derivatives (5), carbohydrates (2), nucleosides and nucleotide analogues (1), ribose phosphates (1), phosphates (1), steroids and derivatives (2), vitamins and derivatives (1), ketones/aldehydes/acids (9), and others (23) (Supplementary Table S1).

#### 3.2.1 Flavonoids and phenols

Flavonoids and phenolic compounds are key metabolites determining the astringency, body, and color of tea infusion. During processing, they undergo oxidation, polymerization, and degradation reactions, contributing to the formation of theaflavins and thearubigins (16). In the present study, procyanidin B2 and taxifolin were the most representative differential flavonoids. Procyanidin B2 increased by 2.10- and 2.93-fold in HF25_vs_F25 and HGZ1_vs_GZ1, respectively, indicating enhanced accumulation of condensed polyphenols. Taxifolin was consistently enriched in PKB-treated samples, potentially due to the hydrolysis of flavonoid glycosides under high-temperature conditions, which releases the corresponding aglycones (17). Meanwhile, several bitter flavonoid glycosides showed significant decreases. Previous studies have indicated that flavonoid glycosides not only possess intrinsic bitterness but can also enhance the bitterness of caffeine (18). For instance, quercetin 3-glucosyl-(1 → 2)-galactoside has an extremely low bitterness threshold, and its content was markedly reduced in HGZ1 (19). Therefore, PKB treatment reduced the levels of bitter flavonoid glycosides, which may collectively contribute to the sweeter and more mellow taste of the tea infusion.

#### 3.2.2 Saccharides and derivatives

Sugars are fundamental contributors to the sweetness of tea infusion. During processing, the degradation of polysaccharides and disruption of cellular structures promote the release of soluble sugars, providing carbon sources for subsequent metabolism (20). Among the detected sugar-related metabolites, mannose exhibited the most pronounced variation, increasing by 2.34–4.79-fold across multiple comparison groups. As mannose constitutes a major component of *P. kingianum* polysaccharides (21), its accumulation likely reflects the degradation and release of these polysaccharides and may represent a critical metabolic basis for the enhanced sweet and mellow taste observed in HGZ1.

#### 3.2.3 Terpenoids

Terpenoids constitute an important class of precursors for tea aroma. According to the data, Ajugol was significantly enriched in F12-treated samples, showing 15.38–20.25-fold increases in HF1, HF25, HF1, HM1, and HGZ1 relative to the corresponding controls (Figure 4A), indicating pronounced modulation of specific terpenoid metabolites. Ajugol, an iridoid glycoside, is a bioactive compound commonly found in several medicinal plants (22). In contrast, other terpenoids, including β-Ionone and Loganin, showed relatively minor changes, whereas Zederone slightly decreased during processing.

#### 3.2.4 Carboxylic acids and derivatives, lipids, alkaloids, and other substances

Among other metabolite classes, lipids and lipid-like molecules, organic acids and their derivatives, alkaloids, and aldehydes exhibited particularly pronounced changes during processing, indicating substantial remodeling of membrane-associated metabolism and secondary metabolic pathways following PKE supplementation. For example, sphingosine increased by 6.89-, 5.52-, 4.36-, 5.01-, and 6.32-fold, respectively, suggesting enhanced sphingolipid-related metabolic activity associated with PKE treatment. Sphingosine is a unique *P. kingianum* metabolite that appears during the *P. kingianum* rolling process and remains present after sufficient firing (23). In addition, the aldehyde compound tetradec-2-enal increased by 5.99-, 6.08-, 10.61-, and 7.34-fold in HF25_vs_F25, HF1_vs_F1, HM1_vs_M1, and HGZ1_vs_GZ1, respectively, indicating its potential contribution to aroma formation during PBK processing. Two alkaloids, Hygrine and 4-hydroxyquinoline, accumulated substantially in HGZ1 and represented key differential alkaloid metabolites distinguishing HGZ1 from GZ1. Several amino acids and their derivatives associated with flavor formation also exhibited significant changes. L-theanine increased by 1.50-, 1.86-, and 1.09-fold in the HF1_vs_F1, HM1_vs_M1, and HGZ1_vs_GZ1 groups, respectively. As a characteristic umami compound in tea, the accumulation of L-theanine can enhance the freshness and sweetness of the infusion while mitigating bitterness (24). Conversely, the bitter amino acid tryptophan decreased to 0.04-, 0.05-, and 0.02-fold in the HR1_vs_R1, HF1_vs_F1, and HM1_vs_M1 groups, respectively, suggesting that it may be further metabolized during PKB processing, thereby contributing to the reduction of tea bitterness (25). Meanwhile, chlorophyll degradation followed a typical transformation pathway during processing, in which chlorophyll was first converted into pheophytin and subsequently into pheophorbide a (26). These degradation products gradually accumulated during the fermentation and drying stages and ultimately contributed to the formation of the characteristic liquor color of PBK.

### 3.3 Analysis of volatile substances in PKB

**Figure 5:**
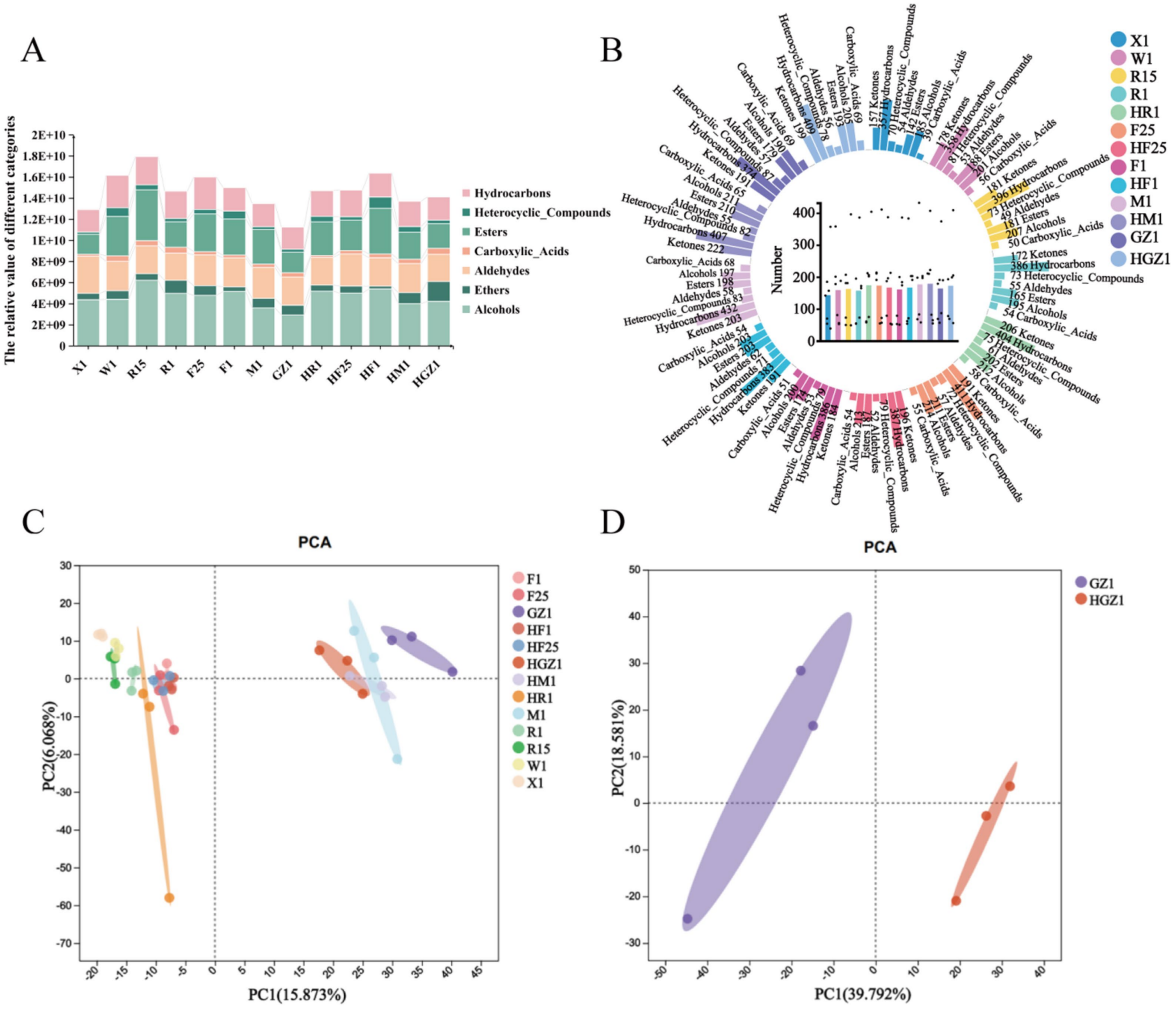
Analysis of volatile flavor compounds during PKB and CK processing. **(A)** Relative abundance of major classes of volatile flavor compounds identified by GC × GC–TOF-MS. **(B)** Distribution of volatile flavor compounds identified at different processing stages. **(C)** Principal component analysis (PCA) score plot of volatile flavor compounds from different processing stages. **(D)** Principal component analysis (PCA) score plot of volatile flavor compounds in GZ1 and HGZ1.

Using comprehensive two-dimensional gas chromatography–time-of-flight mass spectrometry (GC × GC–TOF-MS), a total of 2,319 volatile compounds were identified in PKB and CK. These compounds were classified into hydrocarbons, alcohols, ketones, esters, heterocyclic compounds, acids, aldehydes, and other chemical classes. The distribution of major volatile compound classes is presented in Figure 5B.

To better reflect differences between treatments, the relative abundance of each volatile class was compared (Figure 5A). Compared with CK, PKB exhibited substantially higher abundances of alcohols (4.23 × 10^9^ vs. 2.95 × 10^9^), ethers (1.88 × 10^9^ vs. 9.21 × 10^8^), esters (2.35 × 10^9^ vs. 1.94 × 10^9^), and carboxylic acids (5.63 × 10^8^ vs. 4.09 × 10^8^). Among these classes, alcohols showed the greatest overall abundance in both samples. The increased abundances of alcohols and esters in PKB may contribute to its enhanced floral, fruity, and sweet aroma characteristics.

#### 3.3.1 Electronic nose analysis

Supplementary Figure S1B presents the electronic nose odor radar fingerprint derived from (Supplementary Table S2) data. The radar plot demonstrates differential response patterns of all 10 sensors to volatile compounds in GZ1 versus HGZ1. Four sensors (W1C, W5S, W2S, W2W) showed statistically significant differences (*p* < 0.05, VIP > 1), indicating their critical role in discriminating the black tea volatiles. Of the 10 sensors, W1C shows a significantly higher response to HGZ1 than to GZ1; conversely, the response values of W5S, W2S, and W2W are significantly higher for GZ1 than for HGZ1. These four sensors may therefore contribute significantly to the OPLS-DA model in this experiment. The W1C sensor is particularly sensitive to aromatic components and benzene compounds, which include common aromatic substances found in tea.

#### 3.3.2 Screening of volatile metabolites with flavorome differences

As shown in Figure 5C, principal component analysis (PCA) revealed significant differences in principal component 1 (PC1) and principal component 2 (PC2) between tea samples at different processing stages. PC1 and PC2 explain 15.873 and 6.068% of the data variation, respectively. The fresh leaves (X1), withering (W1), 15-min rolling (R15), rolling completion (R1, HR1), 2.5-h fermentation (F25, HF25), and fermentation completion (F1, HF1) samples are concentrated on the left side of the plot. Samples such as initial firing (M1, HM1) and final firing (GZ1, HGZ1) are distributed on the right side. This indicates that, particularly during the drying process, the processing has the greatest impact on flavor compounds (27) and that the chemical composition of tea leaves undergoes significant changes during the rough firing stage. Significant differences exist between the samples of CK (GZ1) and black tea with added PKE (HGZ1) in principal component 1 (PC1) and principal component 2 (PC2). PC1 and PC2 explain 39.792 and 18.581% of the data variation, respectively (Figure 5D). The GZ1 samples are primarily distributed on the left side of the plot, forming a relatively dispersed cluster. In contrast, the HGZ1 samples are located on the right side, forming a relatively compact cluster. It is also noted that adding PKE significantly alters the flavor compounds in tea, preserving more alcohols, esters, and ketones.

Flavor compounds were screened in the finished product PKB (HGZ1) and the CK (GZ1) using the criteria of VIP > 1 and *p* < 0.05. A total of 537 different flavor compounds were identified (Supplementary Table S3). As shown in Figure 4B, the volatile aromatic components of black tea are primarily composed of alcohol compounds. 2-methyl-2-hexanol levels were significantly higher in the PKB than in the CK and exhibited woody and herbal aromas. Next are aldehydes and ketones, including 3-furaldehyde, 2-tridecanone, 2-hexanone, cyclohexanone, and particularly esters. Esters are renowned for their diverse varieties and subtle fruity aromas, making them some of the most important aromatic compounds in tea (28). Examples include ethyl 3-methylbutanoate, 2-hydroxyethyl acrylate, ethyl 2-hydroxy-3-phenylpropanoate, dimethyl (Z)-but-2-enedioate, (Z)-non-3-en-1-yl acetate, and 2-ethylhexyl salicylate. Six of these compounds showed significantly higher concentrations than the CK. The primary flavor compounds in *P. kingianum* are hydrocarbons, including 4-methylpentadecane, 4-methyltetradecane, cyclopentane (3-(prop-1-en-2-yl)-2-methyl-1-methylenecyclopentane), 8,8-dimethyl-9-methylene-1,5-cycloundecadiene, and 9-methylene-8,8-dimethyl-1,5-cycloundecadiene, (E)-6-hexadecen-4-yne, 4,6-dimethyldodecane, and 2,5,6-trimethyldecane. The increased content of these hydrocarbons in PKB may be due to the PKE solution.

#### 3.3.3 Key aromatic volatile compounds in PKB

The tea’s unique aroma is influenced not only by the content of volatile metabolites, but also by their odor threshold. The Relative Odor Activity Value (ROAV) is a quantitative indicator that assesses the contribution of various volatile metabolites to a tea’s overall aroma. Volatile metabolites with ROAV values of at least 1 are considered key aromatic components of tea samples, and higher ROAV values indicate greater aromatic influence. Those with 0.10 ≤ ROAV < 1 significantly impact tea flavor, and those with 0.01 ≤ ROAV < 0.1 are potential aromatic components (29). Using ROAV, we can precisely identify the key compounds that significantly influence the sensory experience of tea when PKE is added, enabling a deeper exploration of its aromatic complexity.

After processing with PKE, 20 flavor compounds with ROAV values higher than 0.01 were identified in the final product, as shown in (Supplementary Table S4). Of these compounds, seven had ROAV ≥ 1, four had 0.10 ≤ ROAV < 1, and nine had 0.01 < ROAV < 0.1. Flavor compounds with ROAV values of at least 1 include: 2-methylbutanal (ROAV = 100), (E)-2-nonenal (ROAV = 33.03864309), 2-pentylfuran (ROAV = 6.77883206), 2,3-butanedione (ROAV = 7.066135223), heptanal (ROAV = 8.295874587), (E)-2-octenal (ROAV = 2.749859003), and dimethyl sulfide (ROAV = 1.2E-010563251). 2-methylbutanal has a fruity, sweet aroma (30), 2,3-butanedione has a pleasant, buttery aroma (31), heptanal has a citrus and fatty aroma (32), and (E)-2-octenal has a nutty, fresh aroma. These four compounds are relatively prominent flavor compounds compared to the CK. After adding the PKE, the ROAV values of these compounds were higher than those of the CK at the end of kneading (R1, HR1), after 2.5 h of fermentation (F25, HF25), and at the end of fermentation (F1, HF1), rough firing (M1, HM1), and final firing (GZ1, HGZ1).

### 3.4 Metabolic pathways of PKB

**Figure 6:**
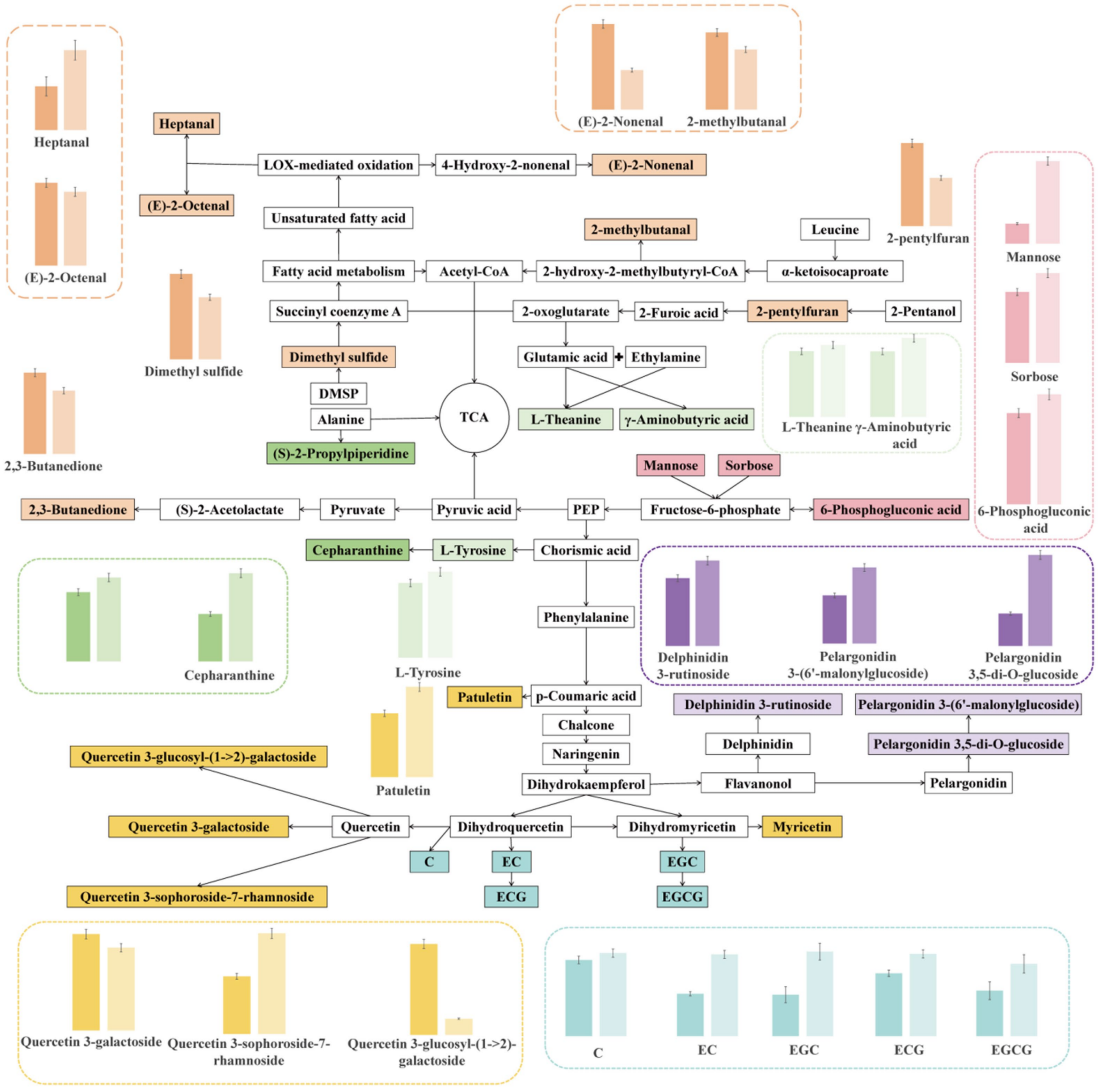
Metabolic pathways of PKB. The main metabolites include flavonoids, alkaloids, amino acids, and flavour components.

Figure 6 illustrates the major metabolic pathways associated with representative differential metabolites and key aroma-active compounds during PKB processing, mainly involving phenylpropanoid/flavonoid metabolism, amino acid metabolism, carbohydrate metabolism, lipid oxidation-related pathways, and sulfur-containing volatile formation.

Catechin components, particularly EC and EGC, were present at higher levels in PKB than in CK. These compounds are mainly derived from the shikimate and phenylpropanoid/flavonoid biosynthetic pathways (33), which are closely associated with tea taste, color, and overall quality formation. Quercetin-derived glycosides, including quercetin 3-glucosyl-(1 → 2)-galactoside, quercetin 3-galactoside, and quercetin 3-sophoroside-7-rhamnoside, are generated through flavonol glycosylation reactions. The reduced accumulation of certain bitter-associated flavonol glycosides in PKB may partly explain its reduced bitterness and enhanced mellow taste. In addition, delphinidin 3-rutinoside, pelargonidin 3-(6′-malonylglucoside), and pelargonidin 3,5-di-O-glucoside are anthocyanin derivatives synthesized through the flavonoid pathway with dihydroflavonols as important intermediates (34, 35).

Amino acid-related metabolites also showed substantial variation during processing. Glutamic acid serves as a central precursor for both L-theanine (36) and γ-aminobutyric acid (GABA) (37). L-theanine is synthesized from glutamic acid and ethylamine through the action of theanine synthase, whereas GABA is generated through the decarboxylation of glutamic acid. These metabolites contribute significantly to the characteristic taste quality and amino acid metabolism of tea.

Several key aroma-active compounds were associated with lipid oxidation and amino acid-derived aroma formation pathways. Heptanal, (E)-2-octenal, and (E)-2-nonenal are commonly produced through the oxidative degradation of unsaturated fatty acids and contribute fresh, fatty, citrus-like, and nutty aroma notes. 2-Pentylfuran is also considered a typical product of lipid oxidation-derived volatile formation (38). In contrast, 2-methylbutanal is an amino acid-derived volatile generated mainly from leucine degradation and Strecker-type reactions (39), contributing malty, fruity, and cocoa-like aroma characteristics. In addition, 2,3-butanedione is associated with pyruvate- and acetolactate-related metabolism and imparts buttery aroma notes. Dimethyl sulfide is a sulfur-containing volatile compound that contributes sweet and vegetal aroma characteristics and may originate from the degradation of sulfur-containing precursors during black tea processing (40).

Although several terpenoid metabolites were detected in the non-volatile metabolite profile, the volatile terpenoid pathway could not be comprehensively reconstructed based on the current dataset. Overall, the differential metabolites identified in PKB were mainly associated with flavonoid metabolism, amino acid metabolism, lipid oxidation-related pathways, and sulfur-containing volatile formation, which collectively contributed to the characteristic flavor profile of PKB.

## 4 Conclusion

This study demonstrated that the incorporation of *P. kingianum* extract (PKE) during black tea processing effectively improved the sensory quality and chemical composition of Dianhong black tea. Compared with the control, *P. kingianum* black tea (PKB) exhibited a darker and more lustrous appearance, a sweeter and mellower taste, and a richer floral–fruity aroma accompanied by herbal notes. These improvements were mainly associated with increased soluble sugar and water-extract contents, as well as changes in taste-related metabolites.

Metabolomics analysis revealed that PKE markedly reshaped the non-volatile metabolite profile during processing, particularly during the fermentation and drying stages. A total of 133 differential non-volatile metabolites were identified, mainly including flavonoids, phenolics, terpenoids, saccharides, lipids, organic acids, and alkaloids. The accumulation of sugar-related metabolites, together with the reduction of several bitter-associated flavonoid glycosides, may contribute to the improved taste characteristics of PKB.

Flavoromics analysis further revealed that PKE enhanced the volatile profile of black tea, particularly alcohols, ketones, and esters. Seven key aroma-active compounds were identified based on Relative Odor Activity Value (ROAV) analysis, among which 2-methylbutanal was the dominant contributor to the fruity and sweet aroma characteristics of PKB.

Overall, this study provides a metabolomics- and flavoromics-based explanation for the quality formation of PKB and demonstrates that PKE-assisted processing is a promising approach for developing novel medicinal–edible tea products with enhanced sensory quality and distinctive flavor characteristics.

## Data Availability

The original contributions presented in the study are included in the article/supplementary material, further inquiries can be directed to the corresponding author/s.
