# Supplementary material for: Metabolomic and flavoromic insights into the effects of Polygonatum kingianum extract during black tea processing
Source: Front Nutr. 2026 Jul 8;13:1882990. doi: 10.3389/fnut.2026.1882990 (PMC13388378; doi:10.3389/fnut.2026.1882990)
Supplement: Supplementary file 1 [file Supplementary_file_1.DOCX]

Supplementary Material

# 1 Supplementary Figures and Tables


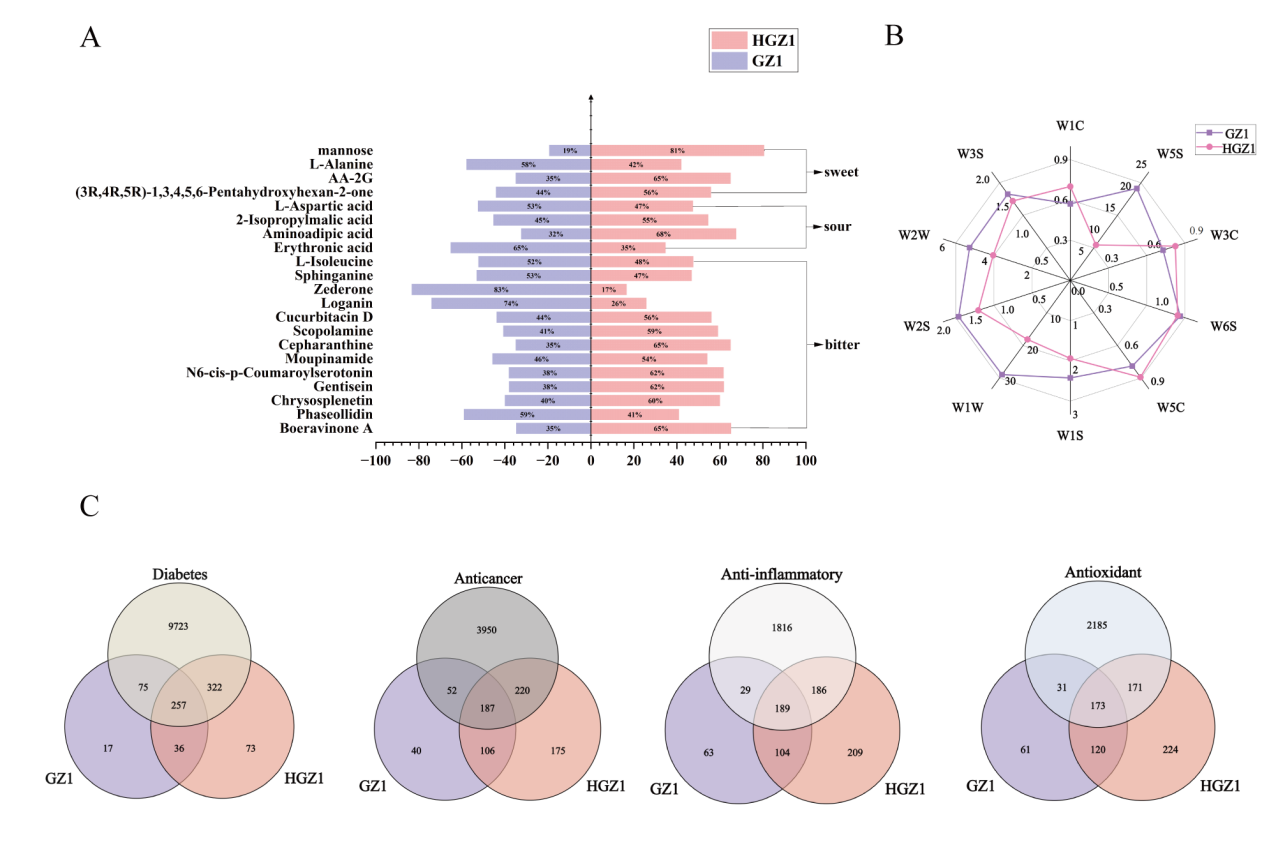


# Figures. S1. (A) Taste presentation of differential metabolites in PKB. (B) Electronic nose radar chart.

# Table S1 The information of 133 differential non-volatile metabolites during the black tea processing, including CAS, relative content, and fold changes.

| **N0** | **Compounds** | **Class** | **CAS** | **X1** | **W1** | **R15** | **R1** | **HR1** | **HR1/R1** | **F25** | **HF25** | **HF25/F25** | **F1** | **HF1** | **HF1/F1** | **M1** | **HM1** | **HM1/M1** | **GZ1** | **HGZ1** | **HGZ1/GZ1** |
| --- | --- | --- | --- | --- | --- | --- | --- | --- | --- | --- | --- | --- | --- | --- | --- | --- | --- | --- | --- | --- | --- |
| 1 | L-Theanine | Carboxylic acids and derivatives | 3081-61-6 | 1.30208E+11 | 1.15058E+11 | 1.06815E+11 | 88726208408 | 57958406221 | 0.65 | 97146178566 | 1.00078E+11 | 1.03 | 69550622677 | 1.04074E+11 | 1.50 | 69524493739 | 1.29325E+11 | 1.86 | 80273747978 | 87898581667 | 1.09 |
| 2 | Tryptophan | Carboxylic acids and derivatives | 73-22-3 | 4146498.511 | 1989047.689 | 1815876.754 | 534366.6555 | 23297.14201 | 0.04 | 1324497.232 | 3166765.561 | 2.39 | 8681868.652 | 399514.9591 | 0.05 | 2536082.869 | 40214.19445 | 0.02 | 1200519.794 | 988478.9873 | 0.82 |
| 3 | Loganin | Terpenoids | 18524-94-2 | 6804375.98 | 4889316.962 | 10024140.27 | 7885636.878 | 2320608.247 | 0.29 | 2720915.75 | 1395242.75 | 0.51 | 1439824.161 | 2250486.974 | 1.56 | 4605855.103 | 5883560.132 | 1.28 | 7785802.832 | 2712385.775 | 0.35 |
| 4 | procyanidin B2 | Flavonoids | 29106-49-8 | 274937548.8 | 234199589 | 287628258.1 | 250770131 | 184851649.9 | 0.74 | 43316754.65 | 90910317.22 | 2.10 | 5767496.786 | 10186729.39 | 1.77 | 4978004.556 | 2846765.384 | 0.57 | 4251348.414 | 12466449.84 | 2.93 |
| 5 | Oleamide | Lipids and lipid-like molecules | 301-02-0 | 135189.5714 | 275990.7259 | 282129.3706 | 813065.2762 | 48618710.88 | 59.80 | 1314345.359 | 52743197.86 | 40.13 | 1519689.761 | 52881461.5 | 34.80 | 8662632.735 | 55610932.59 | 6.42 | 9097546.514 | 55308920.99 | 6.08 |
| 6 | beta-Cryptoxanthin | Terpenoids | 472-70-8 | 100036181.6 | 57502168.67 | 55104739.45 | 98517211.75 | 66719613.36 | 0.68 | 109714597.2 | 73141248.08 | 0.67 | 96362810.63 | 69567561.86 | 0.72 | 78232037.22 | 60987004.17 | 0.78 | 83027593.02 | 67494872.1 | 0.81 |
| 7 | Neoxanthin | Terpenoids | 30743-41-0 | 31762898.12 | 37063723.56 | 34801439.65 | 26647961.64 | 18093323.27 | 0.68 | 22380807.69 | 20812709.8 | 0.93 | 15651632.02 | 29187749.58 | 1.86 | 13986151.01 | 16484898.6 | 1.18 | 12826427.44 | 25892092.93 | 2.02 |
| 8 | Octadecanamide | Others | 124-26-5 | 411596296.3 | 316640584.5 | 305778411 | 272811482 | 438538704.8 | 1.61 | 280246518.3 | 288118486.9 | 1.03 | 250861864.2 | 265385926.9 | 1.06 | 240177244.8 | 497815556 | 2.07 | 324146772.1 | 298857257.3 | 0.92 |
| 9 | Pyridoxine | Vitamins and derivatives | 65-23-6 | 5363426.263 | 7588110.34 | 8293369.907 | 8668105.358 | 19880145.87 | 2.29 | 8378893.926 | 17894786.84 | 2.14 | 7682628.412 | 16811328.9 | 2.19 | 10145672.12 | 19191476.98 | 1.89 | 14306154.06 | 16884747.12 | 1.18 |
| 10 | Protopanaxatriol | Terpenoids | 34080-08-5 | 21749411.93 | 17467993.49 | 18036021.37 | 33597552.22 | 25754932.6 | 0.77 | 43901424.59 | 27544186.18 | 0.63 | 41860634.71 | 32985378 | 0.79 | 39771693.8 | 27610534.31 | 0.69 | 33900767.84 | 28340447.2 | 0.84 |
| 11 | Sequoyitol | Others | 7600-53-5 | 10820434728 | 9834131836 | 6957414517 | 10751316035 | 3933719023 | 0.37 | 7158254262 | 6530635451 | 0.91 | 7379658415 | 8014722299 | 1.09 | 7415102685 | 7505312766 | 1.01 | 8628870279 | 7812053655 | 0.91 |
| 12 | Hygrine | Alkaloids | 45771-52-6 | 6948947.379 | 11913070.31 | 13699410 | 13831886.23 | 11673360.38 | 0.84 | 9290222.305 | 101140505.8 | 10.89 | 11827906.57 | 9776037.403 | 0.83 | 8528246.589 | 37699516.31 | 4.42 | 8240235.136 | 66910043.87 | 8.12 |
| 13 | Scutellarein 4-methyl ether | Flavonoids | 6563-66-2 | 509889.7507 | 92997.84106 | 235042.9703 | 389503.4406 | 414586.8043 | 1.06 | 1076291.142 | 1013931.339 | 0.94 | 1614471.13 | 1217300.5 | 0.75 | 1033555.801 | 1681931.707 | 1.63 | 1206562.268 | 1477827.772 | 1.22 |
| 14 | Betulalbuside A | Terpenoids | 64776-96-1 | 781835.6185 | 1058913.996 | 1290794.68 | 2431530.963 | 4794348.469 | 1.97 | 2419277.469 | 4698547.423 | 1.94 | 1443587.145 | 5180801.888 | 3.59 | 2024188.325 | 3729083.432 | 1.84 | 1996264.188 | 4658720.84 | 2.33 |
| 15 | beta-Ionone | Terpenoids | 79-77-6 | 60305335.53 | 54258882.53 | 57253178.23 | 34683813.84 | 41490225.91 | 1.20 | 14768379.29 | 20452580.51 | 1.38 | 13291904.45 | 15843439.57 | 1.19 | 8866777.663 | 14643679.16 | 1.65 | 10798686.78 | 15541876.24 | 1.44 |
| 16 | Deoxyribose 1-phosphate | Ribose phosphates | 17039-17-7 | 22172258.8 | 48373442.13 | 59314763.19 | 85540453.69 | 63467290.44 | 0.74 | 85713305.02 | 70844269.48 | 0.83 | 86274560.76 | 71912935.49 | 0.83 | 59319759.43 | 48031309.21 | 0.81 | 63634702.91 | 52851669.22 | 0.83 |
| 17 | Zederone | Terpenoids | 7727-79-9 | 1825313.209 | 1642278.206 | 1488161.871 | 1655510.005 | 1617043.012 | 0.98 | 1508205.935 | 1200232.335 | 0.80 | 1304745.41 | 1522105.563 | 1.17 | 2391834.069 | 739846.0256 | 0.31 | 3111400.914 | 622111.9148 | 0.20 |
| 18 | Hypoxanthine | Nucleosides and nucleotide analogues | 68-94-0 | 832244943.2 | 732150960.8 | 692368580.3 | 465193148.1 | 230672244.6 | 0.50 | 77723189.66 | 150880888.7 | 1.94 | 38096688.22 | 96239928.61 | 2.53 | 50598937.05 | 44938839.98 | 0.89 | 68518924.99 | 166236156.7 | 2.43 |
| 19 | Artemisinic alcohol | Others | 125184-95-4 | 13577513.81 | 8960598.837 | 12002190.26 | 11459144.49 | 9738711.165 | 0.85 | 10216894.69 | 10420097.14 | 1.02 | 14740916.98 | 11863403.45 | 0.80 | 8848857.675 | 10551249.76 | 1.19 | 11383252.13 | 8900660.196 | 0.78 |
| 20 | 24-Methylenecycloartane-3,28-diol | Others | 149252-86-8 | 29611581.92 | 2576650.785 | 5989277.513 | 33779472.74 | 6702411.809 | 0.20 | 16897824.49 | 1550237.426 | 0.09 | 19769212.25 | 1179958.803 | 0.06 | 22168503.58 | 7055770.514 | 0.32 | 30587574.3 | 17494566.11 | 0.57 |
| 21 | Isopropyl ferulate | Phenylpropanoids | 59831-94-6 | 194365454.3 | 173489997.5 | 134132053.7 | 157684238.9 | 239352022 | 1.52 | 176956782.6 | 117058449.2 | 0.66 | 188562164.1 | 272534140.4 | 1.45 | 139504637.5 | 229147412.7 | 1.64 | 171272981.8 | 236510668.4 | 1.38 |
| 22 | Pterosin B | Ketones/aldehydes/acids | 60657-37-6 | 410952.9444 | 476822.9719 | 527672.654 | 2702751.209 | 10127302.84 | 3.75 | 3260811.405 | 11410009.07 | 3.50 | 3951819.859 | 11325435.97 | 2.87 | 2204993.279 | 7291793.075 | 3.31 | 2072205.966 | 8220503.463 | 3.97 |
| 23 | 3-(4-hydroxyphenyl)lactate | Phenols | 306-23-0 | 115122678.8 | 105373438.7 | 78505819.03 | 61706253.76 | 212409432.5 | 3.44 | 91055821.83 | 291814749.5 | 3.20 | 92781367.73 | 278062003.5 | 3.00 | 68031798.33 | 195690546.2 | 2.88 | 55375578.55 | 107104352.6 | 1.93 |
| 24 | 12,13-DiHOME | Lipids and lipid-like molecules | 263399-35-5 | 3530470.973 | 5566525.663 | 5787456.593 | 4439316.49 | 4744059.944 | 1.07 | 2385667.95 | 6090971.873 | 2.55 | 1799768.719 | 3519506.528 | 1.96 | 562976.9663 | 2593863.571 | 4.61 | 1298151.771 | 2457431.027 | 1.89 |
| 25 | Neocembrene | Terpenoids | 31570-39-5 | 3858605.773 | 3496734.503 | 3326818.125 | 5236056.023 | 3891482.93 | 0.74 | 5087996.61 | 3987584.452 | 0.78 | 4688084.872 | 3677057.989 | 0.78 | 3369153.753 | 2261457.281 | 0.67 | 2768967.792 | 3046884.296 | 1.10 |
| 26 | Samidin | Phenylpropanoids | 477-33-8 | 11782828.19 | 8536074.903 | 16394087.87 | 6431828.507 | 2346106.787 | 0.36 | 15023982.91 | 17651703.34 | 1.17 | 120048859.9 | 5845452.105 | 0.05 | 32112368.68 | 2714362.485 | 0.08 | 1161147.355 | 43367.00304 | 0.04 |
| 27 | Soyasapogenol E | Others | 6750-59-0 | 6278048.389 | 489540.0073 | 370902.1039 | 2096517.472 | 1097722.863 | 0.52 | 5821536.636 | 6262916.102 | 1.08 | 46063191.05 | 1679657.493 | 0.04 | 7915035.393 | 552597.6298 | 0.07 | 723958.0666 | 582604.0687 | 0.80 |
| 28 | 2-oxo-5-Methylthiopentanoic acid | Ketones/aldehydes/acids | 107872-90-2 | 274664484.1 | 289743358.1 | 257495498 | 211754420.9 | 205447074.4 | 0.97 | 91453938.35 | 85444694.33 | 0.93 | 37855371.66 | 57011024.37 | 1.51 | 51098031.21 | 45108480.64 | 0.88 | 49833801.82 | 65510387.75 | 1.31 |
| 29 | 14-hydroxysprengerinin C | Steroids and derivatives | 1111088-89-1 | 2135958312 | 691659918.7 | 1226976954 | 1345658811 | 1036685194 | 0.77 | 1015828636 | 1141936427 | 1.12 | 1636084500 | 1452488037 | 0.89 | 1031213490 | 1735883335 | 1.68 | 1446767278 | 700369537.3 | 0.48 |
| 30 | Cepharanthine | Alkaloids | 481-49-2 | 4375017.732 | 4064852.451 | 3610813.351 | 3453264.738 | 3441262.732 | 1.00 | 6652139.919 | 3222403.718 | 0.48 | 12822579.51 | 8127544.203 | 0.63 | 49909780.63 | 22856470.72 | 0.46 | 43753433.83 | 81440568.16 | 1.86 |
| 31 | Homocitrulline | Carboxylic acids and derivatives | 1190-49-4 | 7184922.236 | 523674.6996 | 1995602.854 | 15966677.92 | 3246303.45 | 0.20 | 18828827.29 | 11891568.44 | 0.63 | 16301609.23 | 14459190.56 | 0.89 | 13117149.94 | 6854554.039 | 0.52 | 15084873.4 | 11243891.59 | 0.75 |
| 32 | Kinetin | Others | 525-79-1 | 15190150.86 | 11406162.08 | 10908352.59 | 18770314.17 | 30551239.29 | 1.63 | 14456867.01 | 9487379.54 | 0.66 | 22567164.25 | 12095643.55 | 0.54 | 26003848.79 | 26269957.63 | 1.01 | 18922075.92 | 18746500.39 | 0.99 |
| 33 | (3R,4R,5R)-1,3,4,5,6-Pentahydroxyhexan-2-one | Saccharides and derivatives | 2074739-93-6 | 700773029.2 | 1110006421 | 590429030.3 | 418023106.8 | 935782154.9 | 2.24 | 783830000.7 | 506139144.4 | 0.65 | 574126649.9 | 578559429 | 1.01 | 433040033.5 | 367184812.1 | 0.85 | 580963531 | 735238950.9 | 1.27 |
| 34 | 4-Hydroxycoumarin | Phenylpropanoids | 22105-09-5 | 17593392.03 | 12659006.36 | 15951143.45 | 14730798.35 | 8681683.551 | 0.59 | 7297048.096 | 6217166.313 | 0.85 | 5141905.099 | 3535735.055 | 0.69 | 883891.2669 | 964490.4086 | 1.09 | 1155395.795 | 1529140.912 | 1.32 |
| 35 | Retinal | Others | 116-31-4 | 326228.9641 | 501510.2241 | 488584.8035 | 2998131.181 | 6130907.567 | 2.04 | 4790254.789 | 7621784.167 | 1.59 | 5738172.778 | 8214227.667 | 1.43 | 33334023.55 | 53754398.4 | 1.61 | 28049629.99 | 68468929.77 | 2.44 |
| 36 | Corydalis L | Alkaloids | 483-34-1 | 928008.5413 | 684207.6745 | 2159779.183 | 3474003.693 | 4132816.031 | 1.19 | 2931993.085 | 3191586.505 | 1.09 | 3580903.247 | 3373883.013 | 0.94 | 3079941.087 | 2768941.722 | 0.90 | 2356830.577 | 1779335.82 | 0.75 |
| 37 | 2,3,4-Trihydroxybenzoic acid | Phenols | 610-02-6 | 651623271.6 | 722804190.9 | 792093349 | 319446341.7 | 539444387.8 | 1.69 | 123269182 | 269050851.8 | 2.18 | 108988654.8 | 422593634.8 | 3.88 | 99483258.88 | 113982936.9 | 1.15 | 255398160.8 | 101836961.9 | 0.40 |
| 38 | methyl farnesoate | Others | 10485-70-8 | 1495252.6 | 1406889.183 | 1561478.958 | 1550220.089 | 1761117.539 | 1.14 | 2736500.639 | 2654214.775 | 0.97 | 2016727.792 | 1857624.824 | 0.92 | 968917.1601 | 1072005.713 | 1.11 | 1184106.681 | 945817.4869 | 0.80 |
| 39 | Taxifolin | Flavonoids | 480-18-2 | 1734200.895 | 5335664.081 | 4148031.379 | 6217718.479 | 6936635.674 | 1.12 | 8002311.892 | 8697283.81 | 1.09 | 7493359.476 | 8795450.546 | 1.17 | 13852152.3 | 25568084.65 | 1.85 | 11448383.24 | 21128053.44 | 1.85 |
| 40 | Tetradec-2-enal | Ketones/aldehydes/acids | 51534-36-2 | 34165016.64 | 53130188.7 | 69247292.4 | 33919245.18 | 114661851.8 | 3.38 | 19181050.89 | 114862437.5 | 5.99 | 17078467.11 | 103794652.7 | 6.08 | 11773545.09 | 124938349.5 | 10.61 | 17300878.97 | 127038089.6 | 7.34 |
| 41 | Myo-Inositol | Saccharides and derivatives | 87-89-8 | 12345888.25 | 9619259.163 | 9244966.909 | 9177435.467 | 7369226.706 | 0.80 | 9289854.643 | 7949224.979 | 0.86 | 7541833.945 | 8246497.505 | 1.09 | 6535445.604 | 5704479.759 | 0.87 | 6749184.391 | 4697303.767 | 0.70 |
| 42 | 3-(10-Heptadecenyl)phenol | Phenols | 111047-33-7 | 280526.8002 | 1353089.682 | 1155814.319 | 545802.1147 | 1543231.859 | 2.83 | 790534.965 | 2384307.722 | 3.02 | 923635.2295 | 2167594.899 | 2.35 | 592135.5825 | 1429556.619 | 2.41 | 453628.014 | 1280885.656 | 2.82 |
| 43 | o-Ethyltoluene | Others | 25550-14-5 | 14186828.54 | 18740141.46 | 19833192.94 | 19890065.93 | 53703253.94 | 2.70 | 25479721.38 | 60646354.5 | 2.38 | 25261640.95 | 51600182.54 | 2.04 | 29768932.04 | 54742570.85 | 1.84 | 30401987.51 | 59682326.64 | 1.96 |
| 44 | Epsilon-caprolactam | Others | 9012-16-2 | 107263993.6 | 105802366.3 | 89140302.84 | 92668223.58 | 231715832.8 | 2.50 | 141057282.3 | 191983325.5 | 1.36 | 150180417.3 | 216821409.5 | 1.44 | 132710136.3 | 221814867.8 | 1.67 | 117922843.6 | 243049346.7 | 2.06 |
| 45 | Schidigerasaponin D5 | Steroids and derivatives | 266998-04-3 | 38819891.88 | 17017854.88 | 19806694.3 | 20243357.96 | 23805635.53 | 1.18 | 17466556.34 | 12428360.14 | 0.71 | 7792691.055 | 12553545.05 | 1.61 | 14437326.04 | 11114599.46 | 0.77 | 12616840.21 | 21455937.29 | 1.70 |
| 46 | alpha-D-Glucose | Saccharides and derivatives | 492-62-6 | 2471731.196 | 4783532.326 | 5009933.555 | 7027945.714 | 6704729.094 | 0.95 | 7002110.928 | 4872520.407 | 0.70 | 8148264.788 | 4697598.378 | 0.58 | 4713846.137 | 4294882.49 | 0.91 | 4591838.912 | 5111417.389 | 1.11 |
| 47 | Benzyl alcohol | Others | 100-51-6 | 506278.7442 | 719031.0894 | 568180.8527 | 989588.7529 | 605332.0746 | 0.61 | 1154642.437 | 972687.3178 | 0.84 | 820789.4208 | 1207294.747 | 1.47 | 1239241.446 | 1197058.304 | 0.97 | 1264705.424 | 1426539.787 | 1.13 |
| 48 | Sphingosine | Lipids and lipid-like molecules | 123-78-4 | 196835.7614 | 409287.5516 | 427938.9754 | 894959.5262 | 6164081.924 | 6.89 | 1866598.839 | 10312784.95 | 5.52 | 1969377.249 | 8579445.72 | 4.36 | 1174284.087 | 5884721.1 | 5.01 | 768620.1834 | 4859369.391 | 6.32 |
| 49 | Sesaminol | Phenols | 74061-79-3 | 14213695.85 | 1764007.019 | 2297085.877 | 6619592.693 | 2109200.038 | 0.32 | 14683982.05 | 15915693.95 | 1.08 | 116762957.7 | 5232699.632 | 0.04 | 30274210.29 | 2824215.083 | 0.09 | 1276628.936 | 149276.4101 | 0.12 |
| 50 | 9-Oxooctadecanoic acid | Lipids and lipid-like molecules | 4114-74-3 | 64500507.61 | 65822764.41 | 76091647.95 | 80990419.48 | 74043959.29 | 0.91 | 66761377.12 | 67344696.19 | 1.01 | 67584676.94 | 61111075.12 | 0.90 | 60069793.42 | 134702920.1 | 2.24 | 59799559.88 | 63105240.05 | 1.06 |
| 51 | Ajugol | Terpenoids | 6926-08-5 | 12761440.15 | 10145780.72 | 10629299.36 | 5197163.668 | 79948404.48 | 15.38 | 5535196.805 | 86756377.22 | 15.67 | 4796482.016 | 83667596.86 | 17.44 | 2839590.546 | 57573545.51 | 20.28 | 3071826.119 | 62194242.63 | 20.25 |
| 52 | Stevioside | Terpenoids | 57817-89-7 | 7881245.841 | 7130685.172 | 8607314.447 | 13504779.21 | 4017426.993 | 0.30 | 5958755.881 | 9720915.082 | 1.63 | 3035309.402 | 5393565.493 | 1.78 | 2893999.072 | 2345747.285 | 0.81 | 3265525.735 | 4166312.767 | 1.28 |
| 53 | (-)-Epicatechin 3-O-gallate | Flavonoids | 1257-08-5 | 57036481.85 | 46559789.16 | 46971021.23 | 33815180.57 | 28370939.94 | 0.84 | 8161363.887 | 13480451.66 | 1.65 | 4288188.726 | 9098995.146 | 2.12 | 5451430.58 | 5393735.734 | 0.99 | 4929444.377 | 9930365.109 | 2.01 |
| 54 | D-Sedoheptulose 7-phosphate | Saccharides and derivatives | 89927-08-2 | 1.43115E+11 | 1.82783E+11 | 85343396631 | 2176976330 | 1812092508 | 0.83 | 2355114738 | 2077272602 | 0.88 | 2847305789 | 1609720544 | 0.57 | 1282241235 | 1129097334 | 0.88 | 1552885974 | 1138842235 | 0.73 |
| 55 | 2-Hydroxyadipic acid | Ketones/aldehydes/acids | 18294-85-4 | 244172986.4 | 239751526.9 | 234610869.9 | 139149202.3 | 161771338.2 | 1.16 | 184259925.2 | 111288918.9 | 0.60 | 157828923.3 | 116379001.3 | 0.74 | 114761622.2 | 155996698.5 | 1.36 | 113797633.1 | 93315632.47 | 0.82 |
| 56 | 9(S)-HPODE | Lipids and lipid-like molecules | 29774-12-7 | 305506778.1 | 106886527.7 | 41113620.48 | 126492205.7 | 290097864.8 | 2.29 | 132453166.6 | 281734201.1 | 2.13 | 166886715 | 308965508.3 | 1.85 | 91513837.23 | 212848480.5 | 2.33 | 90067270.88 | 202718512.8 | 2.25 |
| 57 | Kaempferol | Phenols | 520-18-3 | 759320317.1 | 585588774.9 | 670465482.3 | 632820177.7 | 597331485.4 | 0.94 | 409383707.6 | 452654646 | 1.11 | 254756879.7 | 316924372.9 | 1.24 | 137399988.4 | 247603214.4 | 1.80 | 44569446.86 | 170423793 | 3.82 |
| 58 | O-Feruloylquinate | Phenylpropanoids | 2613-86-7 | 4030703.497 | 3949565.432 | 4239494.893 | 28734532.14 | 46349204.68 | 1.61 | 156298784 | 104908294.9 | 0.67 | 244630573.2 | 132658650.1 | 0.54 | 139138565.3 | 141557922.8 | 1.02 | 153887392.2 | 94445067.24 | 0.61 |
| 59 | Erythronic acid | Saccharides and derivatives | 13752-84-6 | 368376435.7 | 395603564.7 | 332925287.9 | 482065205 | 337823012.6 | 0.70 | 342010997.8 | 318855164.1 | 0.93 | 354801482.9 | 359551218.6 | 1.01 | 387050128.3 | 300526180.9 | 0.78 | 523623053.2 | 279046322 | 0.53 |
| 60 | Solanesol | Others | 13190-97-1 | 1211073.007 | 1048237.402 | 1134461.721 | 1044723.6 | 700190.7281 | 0.67 | 776756.8385 | 819576.7025 | 1.06 | 1263044.591 | 393320.6154 | 0.31 | 277665.2831 | 275074.2617 | 0.99 | 400581.3424 | 431765.061 | 1.08 |
| 61 | mannose | Saccharides and derivatives | 3458-28-4 | 36871431025 | 37386712620 | 43183812167 | 836270494.9 | 4007764311 | 4.79 | 1313607156 | 3906970440 | 2.97 | 1749681309 | 4099129565 | 2.34 | 23813620818 | 3672334244 | 0.15 | 814551858.5 | 3385309167 | 4.16 |
| 62 | Caffeic acid 3-glucoside | Phenylpropanoids | 24959-81-7 | 72822542.56 | 84339717.18 | 88927774.91 | 90132514.67 | 76942564.46 | 0.85 | 62899889.59 | 125235547.4 | 1.99 | 102865036 | 85617369.2 | 0.83 | 63627450.77 | 82153823.12 | 1.29 | 92571588.24 | 68767576.29 | 0.74 |
| 63 | (-)-Epiafzelechin | Flavonoids | 24808-04-6 | 56109822.97 | 66897705.32 | 55056986.53 | 27231187.16 | 25008873.05 | 0.92 | 4824777.697 | 5090327.024 | 1.06 | 2388994.015 | 4356414.039 | 1.82 | 4756511.527 | 3316314.728 | 0.70 | 3862612.929 | 6720002.458 | 1.74 |
| 64 | Thymine | Others | 65-71-4 | 12990068.62 | 13886466.38 | 5309201.476 | 5605815.814 | 3160881.59 | 0.56 | 2490587.204 | 2613276.822 | 1.05 | 2383917.571 | 871368.1082 | 0.37 | 2973964.854 | 1480476.858 | 0.50 | 1409339.74 | 4562902.848 | 3.24 |
| 65 | 4-Hydroxy-2-oxoglutaric acid | Carboxylic acids and derivatives | 1187-99-1 | 640161659.5 | 508139826.7 | 219010512.8 | 266212859 | 161260186.9 | 0.61 | 132498956.3 | 135414566.9 | 1.02 | 144359615.2 | 63815222.43 | 0.44 | 56519768.74 | 59326960.64 | 1.05 | 53636660.86 | 73168690.13 | 1.36 |
| 66 | Vincetoxicoside B | Flavonoids | 22007-72-3 | 3585323.131 | 7175705.243 | 3528042.442 | 7251369.965 | 6681770.784 | 0.92 | 12264890.99 | 11366670.1 | 0.93 | 9025827.755 | 11968484.29 | 1.33 | 5710422.947 | 14039509.86 | 2.46 | 4625440.706 | 7362458.373 | 1.59 |
| 67 | Prostaglandin E2 | Lipids and lipid-like molecules | 363-24-6 | 40346056.99 | 33355251.33 | 24930577.43 | 9807020.9 | 6199172.872 | 0.63 | 6221342.759 | 2927801.407 | 0.47 | 4452624.549 | 1917963.829 | 0.43 | 1058890.948 | 1703492.565 | 1.61 | 713837.4767 | 2092041.617 | 2.93 |
| 68 | 4-Hydroxybenzoic acid | Phenols | 99-96-7 | 34730342.28 | 32580766.7 | 35826487.21 | 26035017.4 | 28571733.96 | 1.10 | 17083981.44 | 20297573.38 | 1.19 | 3788080.456 | 9847379.52 | 2.60 | 10589969.99 | 4583475.218 | 0.43 | 4342005.19 | 20987592.69 | 4.83 |
| 69 | Medicagenic | Terpenoids | 599-07-5 | 8986176.201 | 8620372.687 | 10302472.85 | 19680056.76 | 7640471.528 | 0.39 | 14053039.94 | 11506187.23 | 0.82 | 11932242.23 | 22903410.56 | 1.92 | 16949206.01 | 17860690.62 | 1.05 | 20633844.2 | 11499994.5 | 0.56 |
| 70 | secologanin | Terpenoids | 19351-63-4 | 1077685571 | 1174355239 | 927129182.2 | 221670509.5 | 424093529.9 | 1.91 | 89533063.03 | 155619758.9 | 1.74 | 107991598.1 | 118223233 | 1.09 | 54316791.85 | 93569462.5 | 1.72 | 50915532.56 | 96214161.3 | 1.89 |
| 71 | cis-Melilotoside | Phenylpropanoids | 2446-60-8 | 88661126.4 | 68552522.36 | 95315508.72 | 94352764.17 | 82871424.8 | 0.88 | 94052146.69 | 83624249.32 | 0.89 | 63934571.47 | 91634481.44 | 1.43 | 21762827.27 | 18011857.36 | 0.83 | 16294349.61 | 14892844.76 | 0.91 |
| 72 | Xanthylic acid | Phosphates | 523-98-8 | 674566.4405 | 1196917.275 | 2462480.74 | 6426926.114 | 5174983.842 | 0.81 | 7845823.659 | 4724312.784 | 0.60 | 8375011.97 | 5198860.626 | 0.62 | 6435423.735 | 5961013.992 | 0.93 | 7357286.116 | 5742329.094 | 0.78 |
| 73 | Syringin | Phenylpropanoids | 118-34-3 | 5043293.597 | 5928445.918 | 10132771.35 | 21928185.63 | 34294857.99 | 1.56 | 19399681.12 | 37187226.94 | 1.92 | 23253487.19 | 31865380.51 | 1.37 | 14451994.99 | 24917091.35 | 1.72 | 11020434.66 | 25585430.32 | 2.32 |
| 74 | Cyclic AMP | Others | 60-92-4 | 70418951.82 | 30612173.63 | 25921933.08 | 16640782.37 | 18204940.95 | 1.09 | 6677459.313 | 6914571.744 | 1.04 | 2947431.893 | 4548627.698 | 1.54 | 3550246.369 | 2914945.333 | 0.82 | 4574669.774 | 6196722.115 | 1.35 |
| 75 | Aesculetin | Phenylpropanoids | 91753-33-2 | 1995538.684 | 1242540.311 | 517299.1379 | 751706.5472 | 2915364.685 | 3.88 | 2667007.739 | 6046394.146 | 2.27 | 5284246.901 | 13508392.57 | 2.56 | 4069977.894 | 5632621.856 | 1.38 | 2778043.593 | 8505097.59 | 3.06 |
| 76 | L-Alanine | Carboxylic acids and derivatives | 56-41-7 | 36763939.64 | 162434505.9 | 163803412.4 | 318384125.7 | 258839371.1 | 0.81 | 463132913.9 | 307235500.9 | 0.66 | 478544617 | 380589180.5 | 0.80 | 556809975.2 | 362284144.9 | 0.65 | 545325770.1 | 396331215.7 | 0.73 |
| 77 | Pheophytin a | Others | 603-17-8 | 6673019.659 | 14229302.56 | 16751938.29 | 12238427.19 | 5057211.872 | 0.41 | 1922348.579 | 3390480.06 | 1.76 | 261314.879 | 1513568.091 | 5.79 | 1111778.237 | 374075.2457 | 0.34 | 653239.3332 | 1746270.873 | 2.67 |
| 78 | phlorisobutyrophenone | Others | 35458-21-0 | 18639051.31 | 13953455.32 | 14048268.21 | 17287242.97 | 17673055.5 | 1.02 | 8048345.458 | 20520116.57 | 2.55 | 16826657.13 | 17944727.26 | 1.07 | 16082196.77 | 38632804.75 | 2.40 | 22901845.12 | 16212772.02 | 0.71 |
| 79 | Phlorizin | Flavonoids | 112318-65-7 | 14205266.42 | 13654947.01 | 14140882.91 | 9259053.95 | 8040860.026 | 0.87 | 3040913.848 | 4009232.353 | 1.32 | 1644098.6 | 2605958.07 | 1.59 | 1833931.181 | 2805041.889 | 1.53 | 1881063.542 | 3282899.579 | 1.75 |
| 80 | 1,2-Dioleoyl-sn-glycero-3-phosphate, sodium salt | Others | 108392-02-5 | 972996.4947 | 666832.2071 | 202949.5386 | 386899.8174 | 11078482.7 | 28.63 | 1105945.279 | 13542185.25 | 12.24 | 13087833.44 | 13398893.25 | 1.02 | 18037218.15 | 17238774.06 | 0.96 | 12806464.62 | 29850466.86 | 2.33 |
| 81 | ent-16-Kaurene | Terpenoids | 20070-61-5 | 3110876.046 | 4128549.24 | 2905377.509 | 6874036.825 | 9949790.226 | 1.45 | 13278131.48 | 8698918.131 | 0.66 | 12315091.01 | 7183903.5 | 0.58 | 10020707.39 | 12389868.54 | 1.24 | 9660742.243 | 10385244.84 | 1.07 |
| 82 | Pelargonidin 3,5-di-O-glucoside | Flavonoids | 177334-58-6 | 5275287.383 | 11324786.58 | 9716330.191 | 10862806.17 | 16443430.82 | 1.51 | 4306739.356 | 3529024.834 | 0.82 | 4512276.476 | 2302719.742 | 0.51 | 933926.9492 | 605569.3341 | 0.65 | 584409.579 | 1645705.344 | 2.82 |
| 83 | 2-Methylcitric acid | Ketones/aldehydes/acids | 6061-96-7 | 12786300.7 | 11848874.58 | 10400899.35 | 12749181.95 | 14565713.31 | 1.14 | 12933673.97 | 8080699.485 | 0.62 | 9127064.986 | 9017896.253 | 0.99 | 91840901.03 | 67387359.42 | 0.73 | 100882891.9 | 104738206.7 | 1.04 |
| 84 | N,N'-diacetylchitobiose | Saccharides and derivatives | 35061-50-8 | 9567794.55 | 11476463.18 | 14641613.85 | 13647724.98 | 11515802.64 | 0.84 | 14479596.92 | 13063895.46 | 0.90 | 16441378.31 | 16800914.8 | 1.02 | 11761265.64 | 24371608.89 | 2.07 | 19900806.55 | 17623456.5 | 0.89 |
| 85 | Maesopsin | Flavonoids | 5989-16-2 | 112680607.6 | 159340776 | 173970360.2 | 508280801.9 | 463561810.2 | 0.91 | 543110162.7 | 550134854.4 | 1.01 | 85743812.44 | 41731307.6 | 0.49 | 61537729.23 | 56565504.28 | 0.92 | 60729631.62 | 43966126.34 | 0.72 |
| 86 | Leukoefdin | Flavonoids | 98919-67-6 | 40770.78051 | 32337.32181 | 76395.42981 | 4537655.731 | 3905306.826 | 0.86 | 3771503.868 | 5876182.894 | 1.56 | 2791288.351 | 4037623.058 | 1.45 | 1795425.135 | 3381020.128 | 1.88 | 1782699.589 | 3245388.272 | 1.82 |
| 87 | D-Glucurono-6,3-lactone | Ketones/aldehydes/acids | 32449-92-6 | 28143275.27 | 31185267.72 | 35812276.82 | 35068747.76 | 40401918.2 | 1.15 | 39569689.6 | 27450096 | 0.69 | 27363106.19 | 31979991.44 | 1.17 | 35546357.57 | 24514752.37 | 0.69 | 48229035.82 | 33885155.42 | 0.70 |
| 88 | 4-Hydroxyquinoline | Alkaloids | 611-36-9 | 33558910.32 | 24513272.21 | 21828353.5 | 22500426.88 | 10859675.35 | 0.48 | 16125223.12 | 14042725.1 | 0.87 | 17324889.09 | 11263930.95 | 0.65 | 792486.8618 | 1697622.523 | 2.14 | 741127.8828 | 1814378.644 | 2.45 |
| 89 | Phaseollidin | Flavonoids | 37831-70-2 | 39077351.35 | 53721739.62 | 73751945.87 | 84986125.39 | 80914249.73 | 0.95 | 108396996.6 | 105209283.9 | 0.97 | 125588262.4 | 103900497.8 | 0.83 | 93635288.24 | 74089565.08 | 0.79 | 103595259.2 | 71843028.35 | 0.69 |
| 90 | Santin | Flavonoids | 27782-63-4 | 1404005.864 | 887771.2293 | 759187.467 | 1655315.898 | 760221.0297 | 0.46 | 3377058.586 | 976858.8551 | 0.29 | 1172784.139 | 932864.7627 | 0.80 | 1002557.768 | 884112.9624 | 0.88 | 918248.4459 | 926694.0805 | 1.01 |
| 91 | Gentisein | Phenols | 529-49-7 | 52196.87281 | 35778.45501 | 314409.1714 | 2771706.501 | 2973429.026 | 1.07 | 2111492.663 | 2459420.6 | 1.16 | 1170750.975 | 1891818 | 1.62 | 2497066.726 | 2387814.968 | 0.96 | 2024635.568 | 3289445.865 | 1.62 |
| 92 | 2-Isopropylmalic acid | Carboxylic acids and derivatives | 49601-06-1 | 573283728.6 | 513500344.1 | 574949583.6 | 710572226.2 | 887376656 | 1.25 | 455308016.2 | 775972962.9 | 1.70 | 460794031.5 | 747686924.9 | 1.62 | 899388872.6 | 1147774686 | 1.28 | 901408577.4 | 1085727298 | 1.20 |
| 93 | Aspirin | Carboxylic acids and derivatives | 11126-35-5 | 332323.9431 | 403252.2735 | 339487.5158 | 2125771.467 | 9742144.337 | 4.58 | 3082371.111 | 9727381.442 | 3.16 | 4227467.441 | 10785920.2 | 2.55 | 2501998.82 | 7306025.641 | 2.92 | 1504373.778 | 6684694.54 | 4.44 |
| 94 | n-Butyl acetate | Others | 123-86-4 | 1818139.077 | 3154138.617 | 1991509.078 | 3544616.071 | 38284025.48 | 10.80 | 17745160.09 | 33202604.5 | 1.87 | 17665130.38 | 34886300.98 | 1.97 | 18057457.05 | 29657894.61 | 1.64 | 13015410.38 | 18975739.46 | 1.46 |
| 95 | Quercetin 3-sophoroside-7-rhamnoside | Flavonoids | 64828-40-6 | 2628466.845 | 4599531.168 | 4293999.825 | 4133254.859 | 30961153.17 | 7.49 | 18247787.72 | 48361189.63 | 2.65 | 21572780.49 | 37861284.2 | 1.76 | 23285563.43 | 56772246.95 | 2.44 | 25916220.87 | 45244704.63 | 1.75 |
| 96 | Citric acid | Carboxylic acids and derivatives | 77-92-9 | 950017.1969 | 2257853.602 | 1927285.817 | 4846152.542 | 4634806.434 | 0.96 | 8577716.05 | 8127311.487 | 0.95 | 11405399.68 | 8486877.516 | 0.74 | 14285487.7 | 10972728.55 | 0.77 | 15891149.56 | 13205283.76 | 0.83 |
| 97 | Combretol | Flavonoids | 5084-19-5 | 62499497.09 | 90094144.62 | 93651351.5 | 120605461.4 | 121458776 | 1.01 | 71380364.44 | 41341669.57 | 0.58 | 45827684.46 | 20899113.32 | 0.46 | 14546943.87 | 14473281.58 | 0.99 | 7713840.55 | 7909188.393 | 1.03 |
| 98 | alpha-Hydroxyisobutyric acid | Carboxylic acids and derivatives | 594-61-6 | 160678475.3 | 158460633.8 | 177716098.4 | 175880791.2 | 204591781.3 | 1.16 | 143562198.1 | 248466061.9 | 1.73 | 168534016.9 | 207964946.1 | 1.23 | 160265386.6 | 198121552.2 | 1.24 | 234567272.8 | 155174234.9 | 0.66 |
| 99 | Cornuside | Terpenoids | 131189-57-6 | 3632321.429 | 4150252.041 | 4665578.338 | 4524160.397 | 3971026.297 | 0.88 | 877180.2758 | 2413905.666 | 2.75 | 717160.6501 | 1977965.662 | 2.76 | 724852.6033 | 507001.679 | 0.70 | 286327.758 | 1228521.768 | 4.29 |
| 100 | Sativan | Flavonoids | 41743-86-6 | 3570498.254 | 3261231.361 | 1000700.864 | 2149299.856 | 3913245.56 | 1.82 | 2644327.776 | 3853762.351 | 1.46 | 513275.0608 | 2879773.016 | 5.61 | 1854297.072 | 1825646.905 | 0.98 | 390473.1018 | 3350629.157 | 8.58 |
| 101 | Boeravinone A | Flavonoids | 114567-33-8 | 1324093.188 | 2536545.408 | 3191237.1 | 9980985.272 | 18536925.32 | 1.86 | 11203567.49 | 18913954.42 | 1.69 | 12945829.45 | 18305772.33 | 1.41 | 5536529.269 | 9821051.521 | 1.77 | 5442973.495 | 10216089.15 | 1.88 |
| 102 | Moschamine | Phenols | 193224-22-5 | 9618573.711 | 22910242.08 | 16234054.01 | 21391064.48 | 15145740.31 | 0.71 | 16614424.89 | 14083331.94 | 0.85 | 13771559.19 | 18112008.27 | 1.32 | 7655374.307 | 16218624.98 | 2.12 | 17759016.61 | 18651426.84 | 1.05 |
| 103 | Lamiide | Terpenoids | 27856-54-8 | 114880882.1 | 16480279.18 | 16838272.38 | 115734222.3 | 69703090.33 | 0.60 | 44731695.12 | 50955767.09 | 1.14 | 15220394.91 | 24017946.5 | 1.58 | 6338421.64 | 7410142.971 | 1.17 | 7369888.058 | 9752931.686 | 1.32 |
| 104 | Myricetin | Flavonoids | 529-44-2 | 74703360.36 | 83815636.96 | 70094817.32 | 49466206.23 | 52750931.73 | 1.07 | 22959389.9 | 25380389.46 | 1.11 | 10215051.12 | 15688022.37 | 1.54 | 12487540.2 | 9420198.315 | 0.75 | 16207264.65 | 11255290.12 | 0.69 |
| 105 | 2-Tridecanone | Ketones/aldehydes/acids | 593-08-8 | 3675368.673 | 6673636.705 | 8400773.155 | 17843082.33 | 32138965.44 | 1.80 | 17235227.07 | 32957463.1 | 1.91 | 18478612.04 | 26458757.94 | 1.43 | 12229560.95 | 27783089.45 | 2.27 | 10616555.35 | 20403376.06 | 1.92 |
| 106 | corytuberine | Alkaloids | 517-56-6 | 52775.79965 | 727670.7466 | 1449008.282 | 3513210.191 | 2473618.178 | 0.70 | 4399750.322 | 4452238.613 | 1.01 | 5588631.367 | 4670545.799 | 0.84 | 5896519.222 | 4934786.45 | 0.84 | 5903060.947 | 3918099.788 | 0.66 |
| 107 | Sinapaldehyde | Phenols | 20649-43-8 | 1567116.291 | 3191728.511 | 2838771.659 | 3951306.438 | 2925622.382 | 0.74 | 5504321.088 | 4569684.228 | 0.83 | 6326699.419 | 4730769.738 | 0.75 | 5845011.37 | 5414447.165 | 0.93 | 5666186.762 | 4551545.427 | 0.80 |
| 108 | Hypoglycin B | Carboxylic acids and derivatives | 502-37-4 | 888838.6146 | 789388.1862 | 807712.6165 | 713816.5336 | 2723196.625 | 3.81 | 897962.8691 | 2925699.027 | 3.26 | 783303.9685 | 2653801.499 | 3.39 | 737715.5455 | 2536137.264 | 3.44 | 663042.6177 | 2564401.686 | 3.87 |
| 109 | Glucosyringicacid | Ketones/aldehydes/acids | 33228-65-8 | 3849735.797 | 8563977.819 | 6412938.753 | 614303.6867 | 546421.6739 | 0.89 | 91511.29625 | 103045.8249 | 1.13 | 45293.09497 | 107341.1586 | 2.37 | 322309.4156 | 167106.0845 | 0.52 | 208289.7348 | 361069.0309 | 1.73 |
| 110 | (S)-Edulinine | Others | 27495-36-9 | 4542724.262 | 10904824.42 | 8603724.517 | 4719832.167 | 3733235.241 | 0.79 | 3340749.533 | 3294930.006 | 0.99 | 4014607.076 | 3980314.094 | 0.99 | 21760873.55 | 65964846.59 | 3.03 | 21394239.2 | 56563073.19 | 2.64 |
| 111 | Shikimic acid | Carboxylic acids and derivatives | 138-59-0 | 2055924.863 | 1962348.483 | 1900495.631 | 2042009.377 | 1839791.933 | 0.90 | 1943018.538 | 1698537.118 | 0.87 | 2094687.135 | 1745920.924 | 0.83 | 1113162.565 | 1039183.186 | 0.93 | 1518359.851 | 1253552.804 | 0.83 |
| 112 | Chelerythrine | Alkaloids | 34316-15-9 | 14461469.34 | 15221445.06 | 5081670.314 | 6199599.256 | 3913139.538 | 0.63 | 4323884.121 | 4797035.674 | 1.11 | 2191574.402 | 3699729.564 | 1.69 | 2531976.979 | 4924064.653 | 1.94 | 2105522.004 | 2789229.49 | 1.32 |
| 113 | Cyanidin 3-rutinoside | Flavonoids | 259813-55-3 | 618529326.3 | 462717378.1 | 403098736.9 | 493639276.6 | 447880830.1 | 0.91 | 553379026.3 | 457376276.4 | 0.83 | 245624601.9 | 469700356.5 | 1.91 | 405113508.4 | 469681748 | 1.16 | 341760067.4 | 452333423.6 | 1.32 |
| 114 | Magnoflorine | Alkaloids | 2141-09-5 | 21635932.89 | 24801243.28 | 52625640.58 | 48424240.12 | 33004816.95 | 0.68 | 43662772.79 | 32185044.08 | 0.74 | 48873870.47 | 30151200.02 | 0.62 | 16924273.69 | 13900682.75 | 0.82 | 21543851.48 | 8141273.61 | 0.38 |
| 115 | Tephrosin | Flavonoids | 76-80-2 | 50875.16588 | 2284437.479 | 1881906.666 | 1484936.838 | 1304050.608 | 0.88 | 1130648.033 | 1207460.928 | 1.07 | 1100761.405 | 896245.6964 | 0.81 | 763327.1259 | 963467.2925 | 1.26 | 939798.0765 | 637922.4297 | 0.68 |
| 116 | Quercetin 3-glucosyl-(1->2)-galactoside | Phenols | 27459-71-8 | 158690114.7 | 153742495.2 | 123832832.9 | 19098292.02 | 51078599.25 | 2.67 | 3037496.864 | 1520559.991 | 0.50 | 3924256.647 | 1313108.793 | 0.33 | 4537984.533 | 3177432.318 | 0.70 | 4525255.233 | 802404.0142 | 0.18 |
| 117 | Gibberellin A3 | Terpenoids | 77-06-5 | 1580543.034 | 326512.3102 | 1004224.571 | 1918250.862 | 1161571.525 | 0.61 | 849961.8797 | 1135423.293 | 1.34 | 618668.4321 | 664458.1612 | 1.07 | 460928.7455 | 244611.2535 | 0.53 | 564406.038 | 229435.1194 | 0.41 |
| 118 | 1,2-Benzoquinone | Others | 20526-43-6 | 11168634.17 | 12714752.61 | 32312019.97 | 30898858.65 | 28954729.16 | 0.94 | 13412507.03 | 14512452.96 | 1.08 | 7397375.201 | 12448470.47 | 1.68 | 24345644.86 | 22495973.41 | 0.92 | 21785606.32 | 31625407.12 | 1.45 |
| 119 | Corchorifatty acid F | Lipids and lipid-like molecules | 185147-97-1 | 130107569.7 | 196025201.7 | 194858537.8 | 492999448.1 | 741382115.3 | 1.50 | 643623923.3 | 964206181.8 | 1.50 | 939408779.3 | 917676677.8 | 0.98 | 363156468.1 | 772065019.3 | 2.13 | 438806953.3 | 606144949.3 | 1.38 |
| 120 | Macarpine | Others | 23594-80-1 | 7819774.823 | 6787915.835 | 1335862.032 | 78200417.71 | 52216987.11 | 0.67 | 107573888.1 | 118649795.5 | 1.10 | 94191540.34 | 110222354.2 | 1.17 | 51625491.86 | 52812773.89 | 1.02 | 47353485.21 | 50025388.87 | 1.06 |
| 121 | Thellungianin G | Phenols | 97180-28-4 | 2290121.693 | 4903074.446 | 3370179.72 | 14128477 | 10242416.64 | 0.72 | 10427621.85 | 20792797.7 | 1.99 | 20502815.41 | 9787845.026 | 0.48 | 6807136.819 | 6731618.641 | 0.99 | 10021095.43 | 6014790.957 | 0.60 |
| 122 | 1,3-Benzenediol | Phenols | 26982-54-7 | 33753877.39 | 17529258.94 | 39799140.41 | 25078846.5 | 16425322.33 | 0.65 | 6239904.05 | 11062520.35 | 1.77 | 1939681.18 | 5308350.002 | 2.74 | 471856.6773 | 1348970.34 | 2.86 | 800689.6282 | 16438419.64 | 20.53 |
| 123 | (+)-Sesamin | Phenylpropanoids | 81602-22-4 | 1644599.872 | 22885026.7 | 12286527.19 | 5874172.325 | 4514524.641 | 0.77 | 2152011.073 | 1841806.029 | 0.86 | 5041490.649 | 1642277.216 | 0.33 | 3527724.478 | 2112493.521 | 0.60 | 5043205.299 | 1291658.833 | 0.26 |
| 124 | 4-O-beta-Glucopyranosyl-cis-coumaric acid | Ketones/aldehydes/acids | 117405-48-8 | 8875113.351 | 9254536.588 | 6715617.642 | 25773289.6 | 17584923.69 | 0.68 | 3280607.712 | 7735265.245 | 2.36 | 1382683.818 | 4674927.528 | 3.38 | 5350131.631 | 5126387.143 | 0.96 | 5047709.013 | 11883812.9 | 2.35 |
| 125 | Ceanothic acid | Terpenoids | 21302-79-4 | 12108353.26 | 4301086.77 | 3702034.396 | 2762415.17 | 6210898.532 | 2.25 | 3372110.014 | 4162048.873 | 1.23 | 4202810.438 | 4137204.437 | 0.98 | 2526616.536 | 8199536.706 | 3.25 | 2626147.057 | 2961739.012 | 1.13 |
| 126 | Ribonolactone | Others | 42417-44-7 | 91096043.97 | 31187793.98 | 30091936.61 | 31319216.33 | 30405457.83 | 0.97 | 29878011.73 | 34622592.72 | 1.16 | 28540239.39 | 33958831.17 | 1.19 | 25713027.12 | 25286172.06 | 0.98 | 16438985.62 | 32687350.42 | 1.99 |
| 127 | geranylhydroquinone | Others | 10457-66-6 | 2984322.671 | 4711358.841 | 5230175.278 | 2917876.075 | 6489482.312 | 2.22 | 9848113.419 | 5820596.654 | 0.59 | 9922037.473 | 6971951.567 | 0.70 | 6679049.915 | 6829413.823 | 1.02 | 7675648.862 | 5823697.893 | 0.76 |
| 128 | 1,3-dihydroxy-N-methylacridone | Alkaloids | 28333-02-0 | 1673398133 | 1804614780 | 1743149571 | 1286882232 | 1029100195 | 0.80 | 454133843.5 | 696890031.9 | 1.53 | 228805491.3 | 366451780.7 | 1.60 | 257745431.7 | 264404651.4 | 1.03 | 237060925.6 | 452983338.9 | 1.91 |
| 129 | Ibuprofen | Carboxylic acids and derivatives | 139466-08-3 | 1317947.596 | 958162.992 | 801934.553 | 484954.4096 | 438575.5567 | 0.90 | 221822.5157 | 68026.74514 | 0.31 | 160748.444 | 105860.0408 | 0.66 | 12876.92402 | 22161.20652 | 1.72 | 11004.00968 | 24543.4887 | 2.23 |
| 130 | Butylated hydroxytoluene | Phenols | 1219805-92-1 | 12599013.22 | 19767165.89 | 10584440.99 | 10098628.68 | 6459362.945 | 0.64 | 10031494.83 | 8719246.233 | 0.87 | 10483421.54 | 7374066.641 | 0.70 | 6878488.563 | 8790910.906 | 1.28 | 8165619.301 | 10385453.68 | 1.27 |
| 131 | Sinapine | Phenols | 18696-26-9 | 32745960.49 | 21579853.12 | 37512035.31 | 31191147.8 | 27737339.86 | 0.89 | 9176552.586 | 14188299.63 | 1.55 | 4262599.292 | 5764704.484 | 1.35 | 4343490.884 | 3792411.911 | 0.87 | 4744317.881 | 10055131.38 | 2.12 |
| 132 | Nomilin | Terpenoids | 1063-77-0 | 6295583.975 | 6310784.054 | 5020023.198 | 2569277.561 | 2430323.419 | 0.95 | 58314.26157 | 364911.7122 | 6.26 | 691511.0131 | 39654.61248 | 0.06 | 319169.8362 | 218206.8742 | 0.68 | 269468.0185 | 188705.2599 | 0.70 |
| 133 | Curcumin | Phenols | 94875-80-6 | 887741.2544 | 516253.076 | 1777226.965 | 5702740.804 | 5925252.325 | 1.04 | 16544351.95 | 15018673.77 | 0.91 | 16088659.76 | 15025325.59 | 0.93 | 10889068.39 | 9944663.176 | 0.91 | 10242806.5 | 7165272.254 | 0.70 |

# Table S2 Flavor Substance Screening

| NO. | Name | Class | CAS | VIP | FC | Pvalue |
| --- | --- | --- | --- | --- | --- | --- |
| 1 | 3-Hexen-1-ol, (E)- | Alcohols | 928-97-2 | 1.49793415 | 0.75 | 0.011814899 |
| 2 | trans-2-Methyl-4-hexen-3-ol | Alcohols | 96346-76-8 | 1.49793415 | 0.75 | 0.011814899 |
| 3 | 5-Hexen-2-ol, 5-methyl- | Alcohols | 50551-88-7 | 1.616882693 | 741.57 | 0.007748982 |
| 4 | 5-Hepten-3-yn-2-ol, 6-methyl-5-(1-methylethyl)- | Alcohols | 63922-41-8 | 1.64312764 | 0 | 0.000820014 |
| 5 | 3-Penten-1-ol, 2-methylene-, (E)- | Alcohols | 116203-78-2 | 1.49793415 | 0.75 | 0.011814899 |
| 6 | 3-Methyl-hepta-1,6-dien-3-ol | Alcohols | 34780-69-3 | 1.49793415 | 0.75 | 0.011814899 |
| 7 | 3-Decen-2-ol, (E)- | Alcohols | 69668-92-4 | 1.560754875 | 0.75 | 0.003704764 |
| 8 | 3,6-Nonadien-1-ol, (E,Z)- | Alcohols | 56805-23-3 | 1.344775367 | 0.86 | 0.047481003 |
| 9 | 3,6,6-Trimethyl-cyclohex-2-enol | Alcohols | 73741-62-5 | 1.549920427 | 0.82 | 0.005053804 |
| 10 | 3,5-Hexadien-2-ol | Alcohols | 3280-51-1 | 1.49793415 | 0.75 | 0.011814899 |
| 11 | 2-Cyclohexen-1-ol, 3,5,5-trimethyl- | Alcohols | 470-99-5 | 1.49793415 | 0.75 | 0.011814899 |
| 12 | 2-Penten-1-ol, 2-methyl-, (Z)- | Alcohols | 16958-20-6 | 1.49793415 | 0.75 | 0.011814899 |
| 13 | 5-Hepten-1-ol, 2,6-dimethyl- | Alcohols | 4234-93-9 | 1.49793415 | 0.75 | 0.011814899 |
| 14 | 4-Hexen-3-ol, 2,5-dimethyl- | Alcohols | 60703-31-3 | 1.49793415 | 0.75 | 0.011814899 |
| 15 | 1,5-Hexadien-3-ol | Alcohols | 924-41-4 | 1.49793415 | 0.75 | 0.011814899 |
| 16 | 1,3-Cyclohexadiene-1-methanol, a,2,6,6-tetramethyl-, (.+-.)- | Alcohols | 102676-97-1 | 1.595245612 | 0.79 | 0.001435881 |
| 17 | 1-Nonanol | Alcohols | 143-08-8 | 1.483137274 | 0.83 | 0.014223877 |
| 18 | Cyclopropyl carbinol | Alcohols | 2516-33-8 | 1.613568179 | 132.37 | 0.008589839 |
| 19 | 1-Propanol | Alcohols | 71-23-8 | 1.49793415 | 0.75 | 0.011814899 |
| 20 | 4,6-Diketo-1-heptanol | Alcohols | 57245-94-0 | 1.49793415 | 0.75 | 0.011814899 |
| 21 | 6-Methylheptane-1,6-diol | Alcohols | 5392-57-4 | 1.49793415 | 0.75 | 0.011814899 |
| 22 | 2-Heptanol, 5-methyl- | Alcohols | 54630-50-1 | 1.49793415 | 0.75 | 0.011814899 |
| 23 | 5-Hexyn-3-ol | Alcohols | 19780-84-8 | 1.49793415 | 0.75 | 0.011814899 |
| 24 | 4-Octanol | Alcohols | 589-62-8 | 1.478812379 | 44.82 | 0.01489546 |
| 25 | 2-Heptanol, 4-methyl- | Alcohols | 56298-90-9 | 1.49793415 | 0.75 | 0.011814899 |
| 26 | 3-Hydroxy-2-methylbutanenitrile | Alcohols | 38046-46-7 | 1.49793415 | 0.75 | 0.011814899 |
| 27 | 1,2-Cyclopentanediol, 3-methyl- | Alcohols | 27583-37-5 | 1.49793415 | 0.75 | 0.011814899 |
| 28 | 3-Heptanol | Alcohols | 589-82-2 | 1.49793415 | 0.75 | 0.011814899 |
| 29 | 3-Butyn-2-ol | Alcohols | 2028-63-9 | 1.640091382 | 0 | 0.001926462 |
| 30 | 2-Isopropyl-5-methyl-1-heptanol | Alcohols | 91337-07-4 | 1.548670109 | 0.55 | 0.005063981 |
| 31 | Propanoic acid, 2-hydroxy-2-methyl-, methyl ester | Alcohols | 2110-78-3 | 1.49793415 | 0.75 | 0.011814899 |
| 32 | 2-Hexanol, 2-methyl- | Alcohols | 625-23-0 | 1.64506427 | 173.5 | 5.43823E-07 |
| 33 | 2-Amino-1,3-propanediol | Alcohols | 534-03-2 | 1.49793415 | 0.75 | 0.011814899 |
| 34 | 2-Hexanol, 2,5-dimethyl-, (S)- | Alcohols | 3730-60-7 | 1.49793415 | 0.75 | 0.011814899 |
| 35 | 1-Octanol | Alcohols | 111-87-5 | 1.467292739 | 0.85 | 0.017264107 |
| 36 | 1-Pentanol | Alcohols | 71-41-0 | 1.348893507 | 0.71 | 0.046156584 |
| 37 | 1-Decanol | Alcohols | 112-30-1 | 1.551306285 | 0.72 | 0.004827333 |
| 38 | 1-Butanol | Alcohols | 71-36-3 | 1.4769513 | 0.65 | 0.015400901 |
| 39 | Heptan-2-yl formate | Alcohols | 103884-53-3 | 1.49793415 | 0.75 | 0.011814899 |
| 40 | 2-Nonenal, (E)- | Aldehydes | 18829-56-6 | 1.62698453 | 0.46 | 0.000189988 |
| 41 | 2-Octenal, (E)- | Aldehydes | 2548-87-0 | 1.342892684 | 0.89 | 0.047476336 |
| 42 | 2-Heptenal, (Z)- | Aldehydes | 57266-86-1 | 1.49793415 | 0.75 | 0.011814899 |
| 43 | 1-Cyclohexene-1-acetaldehyde, 2,6,6-trimethyl- | Aldehydes | 472-66-2 | 1.556491555 | 0.85 | 0.004747612 |
| 44 | 2,4-Hexadienal, (E,E)- | Aldehydes | 142-83-6 | 1.608594801 | 0.71 | 0.000900333 |
| 45 | Octanal | Aldehydes | 124-13-0 | 1.565793373 | 0.7 | 0.00351386 |
| 46 | Propanal, 2,2-dimethyl- | Aldehydes | 630-19-3 | 1.644067383 | 562.87 | 2.84452E-06 |
| 47 | Furfural | Aldehydes | 98-01-1 | 1.645322881 | 0 | 4.68404E-07 |
| 48 | Hexanal | Aldehydes | 66-25-1 | 1.644248213 | 8.54 | 1.81345E-06 |
| 49 | Decanal | Aldehydes | 112-31-2 | 1.494048468 | 0.73 | 0.01258109 |
| 50 | Butanal | Aldehydes | 123-72-8 | 1.336383169 | 0.75 | 0.048964082 |
| 51 | 1H-Pyrrole-2-carboxaldehyde | Aldehydes | 1003-29-8 | 1.618777457 | 0.39 | 0.000394394 |
| 52 | 5-Ethyl-2-furaldehyde | Aldehydes | 23074-10-4 | 1.546593973 | 0.65 | 0.005326192 |
| 53 | 2-Furancarboxaldehyde, 5-methyl- | Aldehydes | 620-02-0 | 1.570360514 | 0.67 | 0.002945618 |
| 54 | 3-Furaldehyde | Aldehydes | 498-60-2 | 1.645554877 | 1174.29 | 7.77043E-08 |
| 55 | Propanal, 3-methoxy- | Aldehydes | 2806-84-0 | 1.49793415 | 0.75 | 0.011814899 |
| 56 | Butanal, 2-methyl- | Aldehydes | 96-17-3 | 1.521985903 | 0.78 | 0.008304944 |
| 57 | Benzeneacetaldehyde, a,2,5-trimethyl- | Aldehydes | 52417-50-2 | 1.49793415 | 0.75 | 0.011814899 |
| 58 | Undecanal | Aldehydes | 112-44-7 | 1.537126772 | 0.71 | 0.006424647 |
| 59 | Piperidine, 2-propyl-, (S)- | Alkaloids and derivatives | 458-88-8 | 1.49793415 | 0.75 | 0.011814899 |
| 60 | Benzenemethanol, a-(3-methyl-2-butenyl)- | Benzenoids | 27644-03-7 | 1.49793415 | 0.75 | 0.011814899 |
| 61 | Benzeneethanol, beta-ethenyl- | Benzenoids | 6052-63-7 | 1.49793415 | 0.75 | 0.011814899 |
| 62 | Benzene,1-methyl-1,2-propadienyl- | Benzenoids | 22433-39-2 | 1.4109627 | 0.84 | 0.029824958 |
| 63 | Benzene, (2-methyl-1-propenyl)- | Benzenoids | 768-49-0 | 1.49793415 | 0.75 | 0.011814899 |
| 64 | Benzene, 2-ethenyl-1,3,5-trimethyl- | Benzenoids | 769-25-5 | 1.489381839 | 0.83 | 0.014088761 |
| 65 | Butanoic acid, 3-methyl-, phenylmethyl ester | Benzenoids | 103-38-8 | 1.535605985 | 0.67 | 0.006354912 |
| 66 | Ethylbenzene | Benzenoids | 100-41-4 | 1.370071825 | 0.68 | 0.038981634 |
| 67 | Tolycaine | Benzenoids | 3686-58-6 | 1.49793415 | 0.75 | 0.011814899 |
| 68 | Thiophene, 2,5-di(benzoylthio)- | Benzenoids | 22511-32-6 | 1.49793415 | 0.75 | 0.011814899 |
| 69 | Benzene, n-butyl- | Benzenoids | 104-51-8 | 1.644428155 | 151.31 | 1.61444E-06 |
| 70 | Benzene, propyl- | Benzenoids | 103-65-1 | 1.393254583 | 0.71 | 0.033140975 |
| 71 | 3,5-Di-tert-butyl-2-hydroxybenzonitrile | Benzenoids | 95091-86-4 | 1.49793415 | 0.75 | 0.011814899 |
| 72 | Benzene, (2-ethoxyethyl)- | Benzenoids | 1817-90-9 | 1.645188635 | 254.04 | 3.38977E-07 |
| 73 | Benzene, (1-methylenepropyl)- | Benzenoids | 2039-93-2 | 1.49793415 | 0.75 | 0.011814899 |
| 74 | Benzene, [1-(2,4-cyclopentadien-1-ylidene)ethyl]- | Benzenoids | 2320-32-3 | 1.49793415 | 0.75 | 0.011814899 |
| 75 | 5-Hydroxy-3-methyl-1-indanone | Benzenoids | 57878-30-5 | 1.632867233 | 0.64 | 0.000134086 |
| 76 | Benzaldehyde, 4-ethyl- | Benzenoids | 4748-78-1 | 1.384441552 | 0.8 | 0.03550629 |
| 77 | Benzaldehyde, 4-chloro- | Benzenoids | 104-88-1 | 1.49793415 | 0.75 | 0.011814899 |
| 78 | Naphthalene, 1,2-dihydro-4-methyl- | Benzenoids | 4373-13-1 | 1.538562459 | 0.54 | 0.00597938 |
| 79 | Phenol, 4-hexyl- | Benzenoids | 2446-69-7 | 1.49793415 | 0.75 | 0.011814899 |
| 80 | Benzene, 4-butyl-1,2-dimethoxy- | Benzenoids | 59056-76-7 | 1.49793415 | 0.75 | 0.011814899 |
| 81 | 4-(4-Methylphenyl)-2-butanone | Benzenoids | 7774-79-0 | 1.49793415 | 0.75 | 0.011814899 |
| 82 | 9H-Fluorene, 3-methyl- | Benzenoids | 2523-39-9 | 1.49793415 | 0.75 | 0.011814899 |
| 83 | 2-Isopropylbenzaldehyde | Benzenoids | 6502-22-3 | 1.49793415 | 0.75 | 0.011814899 |
| 84 | 2-Ethyl-5-n-propylphenol | Benzenoids | 72386-20-0 | 1.49793415 | 0.75 | 0.011814899 |
| 85 | Phenol, 2-ethyl-4-methyl- | Benzenoids | 3855-26-3 | 1.49793415 | 0.75 | 0.011814899 |
| 86 | Phenol, 2-bromo-4-methyl- | Benzenoids | 6627-55-0 | 1.644481965 | 372.03 | 1.65052E-06 |
| 87 | Propanoic acid, 2-phenylethyl ester | Benzenoids | 122-70-3 | 1.485796035 | 0.74 | 0.013822877 |
| 88 | 2-(2-Naphthyl)-2-propanol | Benzenoids | 20351-54-6 | 1.645506208 | 553.42 | 8.17013E-08 |
| 89 | 4-tert-Butyl-1(1-thioxo-2,2-dimethyl-propyl)-benzene | Benzenoids | 72194-24-2 | 1.49793415 | 0.75 | 0.011814899 |
| 90 | 1H-Inden-1-one, 2,3-dihydro-3,3,5,6-tetramethyl- | Benzenoids | 54789-22-9 | 1.645578072 | 1324.99 | 6.96452E-08 |
| 91 | Naphthalene, 1,7-dimethyl- | Benzenoids | 575-37-1 | 1.567761234 | 0.58 | 0.003215516 |
| 92 | Naphthalene, 1,4,6-trimethyl- | Benzenoids | 2131-42-2 | 1.645754724 | 0 | 1.07314E-08 |
| 93 | Naphthalene, 1,4,5-trimethyl- | Benzenoids | 2131-41-1 | 1.645676356 | 2472.14 | 2.59465E-08 |
| 94 | 4-Hydroxy-5-methyl-3-phenyl-d(2)-1,2,4-oxadiazoline | Benzenoids | 16227-04-6 | 1.49793415 | 0.75 | 0.011814899 |
| 95 | Benzene, 1,1'-[ethylidenebis(oxy-2,1-ethanediyl)]bis- | Benzenoids | 122-71-4 | 1.49793415 | 0.75 | 0.011814899 |
| 96 | 1(2H)-Naphthalenone, 3,4-dihydro-4,5,6-trimethyl- | Benzenoids | 30316-31-5 | 1.460293516 | 0.84 | 0.0185972 |
| 97 | 3-(4-(tert-Butyl)phenyl)-2-methylpropan-1-ol, O-acetyl | Benzenoids | 79211-55-5 | 1.49793415 | 0.75 | 0.011814899 |
| 98 | 1-Naphthalenemethanol, 1,2,3,4-tetrahydro-8-methyl- | Benzenoids | 36052-28-5 | 1.49793415 | 0.75 | 0.011814899 |
| 99 | Benzene, (1,3,3-trimethylnonyl)- | Benzenoids | 54986-44-6 | 1.49793415 | 0.75 | 0.011814899 |
| 100 | Orcinol | Benzenoids | 504-15-4 | 1.37398611 | 0.85 | 0.03798127 |
| 101 | 9H-Fluorene, 4-methyl- | Benzenoids | 1556-99-6 | 1.49793415 | 0.75 | 0.011814899 |
| 102 | 1H-Indene, 2,3-dihydro-1,1,2,3,3-pentamethyl- | Benzenoids | 1203-17-4 | 1.644773792 | 190.92 | 7.09063E-07 |
| 103 | Etidocaine | Carboxylic_Acids | 36637-18-0 | 1.49793415 | 0.75 | 0.011814899 |
| 104 | N,N'-Methylenebis(formamide) | Carboxylic_Acids | 6921-98-8 | 1.643476276 | 306.88 | 4.7061E-06 |
| 105 | Acetic acid, chloro-, ethyl ester | Carboxylic_Acids | 105-39-5 | 1.423225401 | 0.36 | 0.026208471 |
| 106 | L-Cysteine sulfinic acid | Carboxylic_Acids | 1115-65-7 | 1.645615136 | 0 | 1.48183E-07 |
| 107 | 1-Propen-2-ol, acetate | Esters | 108-22-5 | 1.49793415 | 0.75 | 0.011814899 |
| 108 | cis-3-Hexenyl cis-3-hexenoate | Esters | 61444-38-0 | 1.380216652 | 0.85 | 0.036821672 |
| 109 | Hexanoic acid, 3-hexenyl ester, (Z)- | Esters | 31501-11-8 | 1.502909724 | 0.72 | 0.010760537 |
| 110 | Butanoic acid, 3-hexenyl ester, (Z)- | Esters | 16491-36-4 | 1.592716798 | 0 | 0.017329493 |
| 111 | 3-Hexen-1-ol, benzoate, (Z)- | Esters | 25152-85-6 | 1.463529059 | 0.75 | 0.017605498 |
| 112 | 2-Propenoic acid, 2-methyl-, oxiranylmethyl ester | Esters | 106-91-2 | 1.642166267 | 0 | 4.03555E-06 |
| 113 | n-Caproic acid vinyl ester | Esters | 3050-69-9 | 1.409147279 | 0.83 | 0.029667083 |
| 114 | 2-Propenoic acid, cyclohexyl ester | Esters | 3066-71-5 | 1.49793415 | 0.75 | 0.011814899 |
| 115 | 2-Propenoic acid, 2-hydroxyethyl ester | Esters | 818-61-1 | 1.640043915 | 225.96 | 2.17833E-05 |
| 116 | 2-Pentenoic acid, 4-oxo-, methyl ester, (Z)- | Esters | 19522-27-1 | 1.49793415 | 0.75 | 0.011814899 |
| 117 | 2,4-Dinitrophenyl crotonate | Esters | 69817-88-5 | 1.49793415 | 0.75 | 0.011814899 |
| 118 | 1,3-Pentadien-2-ol, 4-methyl-, acetate | Esters | 34645-17-5 | 1.49793415 | 0.75 | 0.011814899 |
| 119 | Acetic acid, non-3-enyl ester, cis- | Esters | 13049-88-2 | 1.645385956 | 440.47 | 1.65298E-07 |
| 120 | 2-Hexenoic acid, 2-hexenyl ester, (E,E)- | Esters | 54845-28-2 | 1.502960585 | 0.76 | 0.010809316 |
| 121 | Butanoic acid, 3-methyl-, ethyl ester | Esters | 108-64-5 | 1.643016406 | 166.99 | 6.46404E-06 |
| 122 | Butanoic acid, methyl ester | Esters | 623-42-7 | 1.49793415 | 0.75 | 0.011814899 |
| 123 | Propanoic acid, ethyl ester | Esters | 105-37-3 | 1.451148334 | 0.74 | 0.020447259 |
| 124 | Acetic acid, hexyl ester | Esters | 142-92-7 | 1.583549883 | 1.74 | 0.002205988 |
| 125 | Hexadecanoic acid, ethyl ester | Esters | 628-97-7 | 1.495563009 | 0.65 | 0.012191229 |
| 126 | Propanoic acid, 2-methyl-, ethyl ester | Esters | 97-62-1 | 1.445871295 | 0.69 | 0.020855891 |
| 127 | 1-Butanol, 3-methoxy-, acetate | Esters | 4435-53-4 | 1.49793415 | 0.75 | 0.011814899 |
| 128 | 1,1-Ethanediol, diacetate | Esters | 542-10-9 | 1.49793415 | 0.75 | 0.011814899 |
| 129 | Butanoic acid, 3,7-dimethyl-2,6-octadienyl ester, (E)- | Esters | 106-29-6 | 1.436993011 | 0.77 | 0.02307372 |
| 130 | Benzoic acid, 2-hydroxy-, 2-methylbutyl ester | Esters | 51115-63-0 | 1.49793415 | 0.75 | 0.011814899 |
| 131 | Methyl tetradecanoate | Esters | 124-10-7 | 1.467283362 | 0.73 | 0.016720322 |
| 132 | Propyl 2,4-hexadienecarboxylate | Esters | 88973-62-0 | 1.49793415 | 0.75 | 0.011814899 |
| 133 | Nonanoic acid, ethyl ester | Esters | 123-29-5 | 1.591743427 | 0.56 | 0.001682602 |
| 134 | 2-Butenedioic acid (Z)-, dimethyl ester | Esters | 624-48-6 | 1.641621569 | 145.56 | 1.27909E-05 |
| 135 | Methyl formate | Esters | 107-31-3 | 1.49793415 | 0.75 | 0.011814899 |
| 136 | Methyl pentyl methylphosphonate | Esters | 170082-80-1 | 1.49793415 | 0.75 | 0.011814899 |
| 137 | Diisopropyl methylphosphonate | Esters | 1445-75-6 | 1.49793415 | 0.75 | 0.011814899 |
| 138 | Hexanoic acid, 1-methylhexyl ester | Esters | 6624-58-4 | 1.507131786 | 0.7 | 0.010033321 |
| 139 | Hexyl tiglate | Esters | 16930-96-4 | 1.480640597 | 0.76 | 0.014416187 |
| 140 | Butanoic acid, 2-methyl-, ethyl ester | Esters | 7452-79-1 | 1.574782837 | 0.59 | 0.002814038 |
| 141 | Heptanoic acid, ethyl ester | Esters | 106-30-9 | 1.450770123 | 0.76 | 0.020151675 |
| 142 | Butanoic acid, 3-methylbutyl ester | Esters | 106-27-4 | 1.49793415 | 0.75 | 0.011814899 |
| 143 | Butanoic acid, ethyl ester | Esters | 105-54-4 | 1.522349021 | 0.65 | 0.008064851 |
| 144 | Butanoic acid, 3-methylphenyl ester | Esters | 7476-80-4 | 1.49793415 | 0.75 | 0.011814899 |
| 145 | Benzeneacetic acid, phenyl ester | Esters | 722-01-0 | 1.49793415 | 0.75 | 0.011814899 |
| 146 | Butanoic acid, 2-methyl-, 2-phenylethyl ester | Esters | 24817-51-4 | 1.536077526 | 0.75 | 0.006587481 |
| 147 | Benzoic acid, 2-methylpropyl ester | Esters | 120-50-3 | 1.49793415 | 0.75 | 0.011814899 |
| 148 | n-Heptyl methylphosphonofluoridate | Esters | 162085-82-7 | 1.49793415 | 0.75 | 0.011814899 |
| 149 | 2-Butanone, 4-(acetyloxy)- | Esters | 10150-87-5 | 1.49793415 | 0.75 | 0.011814899 |
| 150 | 2-Ethylhexyl salicylate | Esters | 118-60-5 | 1.644821408 | 96.3 | 8.31592E-07 |
| 151 | Nonanoic acid, 2-oxo-, methyl ester | Esters | 56275-54-8 | 1.49793415 | 0.75 | 0.011814899 |
| 152 | Hexanoic acid, 2-oxo-, methyl ester | Esters | 6395-83-1 | 1.49793415 | 0.75 | 0.011814899 |
| 153 | Ethyl 2-hydroxy-3-phenylpropanoate | Esters | 15399-05-0 | 1.645320026 | 767.1 | 2.31076E-07 |
| 154 | Carbamic acid, 2-chloroethyl ester | Esters | 2114-18-3 | 1.49793415 | 0.75 | 0.011814899 |
| 155 | Propanoic acid, 2-methoxy-, methyl ester | Esters | 17639-76-8 | 1.49793415 | 0.75 | 0.011814899 |
| 156 | 2-Methoxy-1,3-dioxolane | Esters | 19693-75-5 | 1.49793415 | 0.75 | 0.011814899 |
| 157 | 2-Methylbenzyl benzoate | Esters | 80716-36-5 | 1.49793415 | 0.75 | 0.011814899 |
| 158 | 2-Hexanone, 6-(acetyloxy)- | Esters | 4305-26-4 | 1.49793415 | 0.75 | 0.011814899 |
| 159 | 1,3,2-Dioxaborolane, 2,4-diethyl- | Esters | 57633-63-3 | 1.49793415 | 0.75 | 0.011814899 |
| 160 | 1,3,2-Dioxaborinane, 2-ethyl-4-methyl- | Esters | 57633-65-5 | 1.49793415 | 0.75 | 0.011814899 |
| 161 | Cyclohexyl methylphosphonofluoridate | Esters | 329-99-7 | 1.49793415 | 0.75 | 0.011814899 |
| 162 | (Z)-Hex-3-enyl (E)-2-methylbut-2-enoate | Esters | 67883-79-8 | 1.504367272 | 0.77 | 0.010780473 |
| 163 | 3-Hexene, 1-(1-methoxyethoxy)-, (E)- | Ethers | 54340-97-5 | 1.49793415 | 0.75 | 0.011814899 |
| 164 | Cyclohexane, ethoxy- | Ethers | 932-92-3 | 1.61291501 | 812.25 | 0.009944463 |
| 165 | Ethane, 1,1-diethoxy- | Ethers | 105-57-7 | 1.513470828 | 0.8 | 0.008452963 |
| 166 | Ethane, methoxy- | Ethers | 540-67-0 | 1.49793415 | 0.75 | 0.011814899 |
| 167 | Cycloheptane, methoxy- | Ethers | 42604-04-6 | 1.49793415 | 0.75 | 0.011814899 |
| 168 | Dimethyl sulfide | Ethers | 75-18-3 | 1.517291452 | 0.73 | 0.009050912 |
| 169 | Ethanol, 2,2'-oxybis- | Ethers | 111-46-6 | 1.49793415 | 0.75 | 0.011814899 |
| 170 | Benzenamine, 4-methoxy- | Ethers | 104-94-9 | 1.49793415 | 0.75 | 0.011814899 |
| 171 | Butane, 1-methoxy-3-methyl- | Ethers | 626-91-5 | 1.49793415 | 0.75 | 0.011814899 |
| 172 | 4-Benzyloxybenzonitrile | Ethers | 52805-36-4 | 1.517683998 | 386571.05 | 0.041884535 |
| 173 | Butanoic acid, 4-phenoxy- | Ethers | 6303-58-8 | 1.645672922 | 2659.06 | 1.7533E-08 |
| 174 | 1,3-Dioxepin, 4,7-dihydro- | Ethers | 5417-32-3 | 1.49793415 | 0.75 | 0.011814899 |
| 175 | 2(3H)-Furanone, 5-(acetyloxy)dihydro-5-methyl- | Ethers | 57681-51-3 | 1.367882686 | 0.87 | 0.041528265 |
| 176 | 1-Methoxyadamantane | Ethers | 6221-74-5 | 1.544385844 | 0.57 | 0.005409688 |
| 177 | Ethane, 1,2-diethoxy- | Ethers | 629-14-1 | 1.49793415 | 0.75 | 0.011814899 |
| 178 | Hexane, 1,1-diethoxy- | Ethers | 3658-93-3 | 1.49793415 | 0.75 | 0.011814899 |
| 179 | 3-Heptyne, 5-methyl- | Hydrocarbons | 61228-09-9 | 1.49793415 | 0.75 | 0.011814899 |
| 180 | Cyclopentane, 2-propenyl- | Hydrocarbons | 3524-75-2 | 1.49793415 | 0.75 | 0.011814899 |
| 181 | Cyclopentane, 2-methyl-1-methylene-3-(1-methylethenyl)- | Hydrocarbons | 56710-83-9 | 1.64519179 | 159.38 | 3.39268E-07 |
| 182 | 1,7-Octadiene, 2-methyl-6-methylene- | Hydrocarbons | 1686-30-2 | 1.588227012 | 0.76 | 0.001826822 |
| 183 | 1,5-Cycloundecadiene, 8,8-dimethyl-9-methylene- | Hydrocarbons | 62338-54-9 | 1.645393605 | 800.03 | 1.39937E-07 |
| 184 | 6-Hexadecen-4-yne, (E)- | Hydrocarbons | 74744-52-8 | 1.64533264 | 557.81 | 1.38421E-07 |
| 185 | 4,6,8-Trimethylazulene | Hydrocarbons | 941-81-1 | 1.617674916 | 0 | 0.007665289 |
| 186 | 1-Pentene, 3-methyl- | Hydrocarbons | 760-20-3 | 1.49793415 | 0.75 | 0.011814899 |
| 187 | 2-Nonen-4-yne, (E)- | Hydrocarbons | 56392-49-5 | 1.49793415 | 0.75 | 0.011814899 |
| 188 | 2-Methyl-2-bornene | Hydrocarbons | 72540-93-3 | 1.49793415 | 0.75 | 0.011814899 |
| 189 | 1,5-Hexadiene, 2,5-dimethyl- | Hydrocarbons | 627-58-7 | 1.49793415 | 0.75 | 0.011814899 |
| 190 | 2,4,4-Trimethyl-1-hexene | Hydrocarbons | 51174-12-0 | 1.49793415 | 0.75 | 0.011814899 |
| 191 | 1,4-Pentadiene, 2,3,3-trimethyl- | Hydrocarbons | 756-02-5 | 1.49793415 | 0.75 | 0.011814899 |
| 192 | 1-Nonen-4-yne | Hydrocarbons | 31508-12-0 | 1.370641015 | 0.83 | 0.041278856 |
| 193 | 1-Hexen-3-yne | Hydrocarbons | 13721-54-5 | 1.49793415 | 0.75 | 0.011814899 |
| 194 | 1,5-Heptadiene, 2,6-dimethyl- | Hydrocarbons | 6709-39-3 | 1.464151173 | 0.8 | 0.017567387 |
| 195 | 1,5,9,11-Tridecatetraene, 12-methyl-, (E,E)- | Hydrocarbons | 62338-27-6 | 1.49793415 | 0.75 | 0.011814899 |
| 196 | 1,3-Octadiene | Hydrocarbons | 1002-33-1 | 1.406699991 | 0.85 | 0.030782245 |
| 197 | 1,3-Nonadiene, (E)- | Hydrocarbons | 56700-77-7 | 1.430983775 | 0.81 | 0.024865905 |
| 198 | 1,3,5-Heptatriene, (E,E)- | Hydrocarbons | 17679-93-5 | 1.472156383 | 0.82 | 0.016286304 |
| 199 | Cyclohexene, 2-ethenyl-1,3,3-trimethyl- | Hydrocarbons | 5293-90-3 | 1.644640039 | 0.01 | 8.89103E-07 |
| 200 | Cyclopentene, 1,2,3-trimethyl- | Hydrocarbons | 473-91-6 | 1.49793415 | 0.75 | 0.011814899 |
| 201 | 1,E-4,Z-8-Dodecatriene | Hydrocarbons | 83489-22-9 | 1.49793415 | 0.75 | 0.011814899 |
| 202 | 2,3-Octadiene, 7-methyl- | Hydrocarbons | 61129-33-7 | 1.49793415 | 0.75 | 0.011814899 |
| 203 | Cyclohexane, decyl- | Hydrocarbons | 1795-16-0 | 1.49793415 | 0.75 | 0.011814899 |
| 204 | Eicosane | Hydrocarbons | 112-95-8 | 1.49793415 | 0.75 | 0.011814899 |
| 205 | Cyclopropane, pentyl- | Hydrocarbons | 2511-91-3 | 1.49793415 | 0.75 | 0.011814899 |
| 206 | Bicyclo[6.1.0]nonane, 9-(1-methylethylidene)- | Hydrocarbons | 56666-90-1 | 1.49793415 | 0.75 | 0.011814899 |
| 207 | Bicyclo[2.1.0]pentane | Hydrocarbons | 185-94-4 | 1.49793415 | 0.75 | 0.011814899 |
| 208 | Dodecane | Hydrocarbons | 112-40-3 | 1.413892954 | 1.34 | 0.028629338 |
| 209 | Cyclopentane, (2-methylpropylidene)- | Hydrocarbons | 53366-58-8 | 1.49793415 | 0.75 | 0.011814899 |
| 210 | Cyclohexane, 1-ethyl-1,4-dimethyl-, cis- | Hydrocarbons | 62238-30-6 | 1.49793415 | 0.75 | 0.011814899 |
| 211 | Octane, 6-ethyl-2-methyl- | Hydrocarbons | 62016-19-7 | 1.369112292 | 0.02 | 0.038076932 |
| 212 | Undecane, 5-ethyl- | Hydrocarbons | 17453-94-0 | 1.49793415 | 0.75 | 0.011814899 |
| 213 | Pentadecane, 5-methyl- | Hydrocarbons | 25117-33-3 | 1.503162664 | 1.53 | 0.010296055 |
| 214 | Decane, 5-propyl- | Hydrocarbons | 17312-62-8 | 1.49793415 | 0.75 | 0.011814899 |
| 215 | Heptane, 4-ethyl- | Hydrocarbons | 2216-32-2 | 1.49793415 | 0.75 | 0.011814899 |
| 216 | Pentadecane, 4-methyl- | Hydrocarbons | 2801-87-8 | 1.645336923 | 978.22 | 1.45978E-07 |
| 217 | Tetradecane, 4-methyl- | Hydrocarbons | 25117-24-2 | 1.553082952 | 1.38 | 0.004793925 |
| 218 | Dodecane, 4,6-dimethyl- | Hydrocarbons | 61141-72-8 | 1.645578571 | 6736.48 | 5.35102E-08 |
| 219 | Undecane, 3-methyl- | Hydrocarbons | 1002-43-3 | 1.58535483 | 1.34 | 0.001893927 |
| 220 | Tetradecane, 3-methyl- | Hydrocarbons | 18435-22-8 | 1.369970867 | 0.8 | 0.040440865 |
| 221 | 2-Pentyne | Hydrocarbons | 627-21-4 | 1.49793415 | 0.75 | 0.011814899 |
| 222 | Pentadecane, 2-methyl- | Hydrocarbons | 1560-93-6 | 1.551974393 | 1.37 | 0.004641929 |
| 223 | Nonadecane, 2-methyl- | Hydrocarbons | 1560-86-7 | 1.49793415 | 0.75 | 0.011814899 |
| 224 | Octadecane, 2-methyl- | Hydrocarbons | 1560-88-9 | 1.49793415 | 0.75 | 0.011814899 |
| 225 | Decane, 2,5,6-trimethyl- | Hydrocarbons | 62108-23-0 | 1.637602178 | 769.03 | 2.86816E-05 |
| 226 | Heptane, 2,4-dimethyl- | Hydrocarbons | 2213-23-2 | 1.495674361 | 0.46 | 0.012082746 |
| 227 | Heptane, 2,2,4,6,6-pentamethyl- | Hydrocarbons | 13475-82-6 | 1.62971478 | 0 | 0.004578686 |
| 228 | Propane, 2-cyclopropyl- | Hydrocarbons | 3638-35-5 | 1.49793415 | 0.75 | 0.011814899 |
| 229 | Cyclopropane, 1-methyl-2-(1-methylpentyl)- | Hydrocarbons | 62238-06-6 | 1.49793415 | 0.75 | 0.011814899 |
| 230 | Cyclopentane, 1-hexyl-3-methyl- | Hydrocarbons | 61142-68-5 | 1.49793415 | 0.75 | 0.011814899 |
| 231 | Cyclopentane, 1,1,3-trimethyl- | Hydrocarbons | 4516-69-2 | 1.478322139 | 0.28 | 0.015758249 |
| 232 | Propyne | Hydrocarbons | 74-99-7 | 1.49793415 | 0.75 | 0.011814899 |
| 233 | 1,11-Dodecadiyne | Hydrocarbons | 20521-44-2 | 1.49793415 | 0.75 | 0.011814899 |
| 234 | 3-Octen-2-one | Ketones | 1669-44-9 | 1.601260042 | 0.61 | 0.001061469 |
| 235 | 3-Nonen-2-one | Ketones | 14309-57-0 | 1.515571172 | 0.79 | 0.008624715 |
| 236 | Bicyclo[2.2.2]oct-5-en-2-one | Ketones | 2220-40-8 | 1.49793415 | 0.75 | 0.011814899 |
| 237 | 5-Hepten-2-one, 6-methyl- | Ketones | 110-93-0 | 1.357749337 | 0.83 | 0.043640196 |
| 238 | 2-Cyclopenten-1-one, 3-methyl-2-(2,4-pentadienyl)-, (Z)- | Ketones | 22610-79-3 | 1.49793415 | 0.75 | 0.011814899 |
| 239 | 3-Decen-5-one, 2-methyl- | Ketones | 32064-75-8 | 1.49793415 | 0.75 | 0.011814899 |
| 240 | 3,6,6-TRIMETHYL-CYCLOHEX-2-ENONE | Ketones | 23438-77-9 | 1.469180865 | 0.8 | 0.016565048 |
| 241 | 2-Cyclopenten-1-one, 2-(2-butenyl)-3-methyl-, (Z)- | Ketones | 17190-71-5 | 1.49793415 | 0.75 | 0.011814899 |
| 242 | 4,4-Dimethylcyclohexadienone | Ketones | 1073-14-9 | 1.49793415 | 0.75 | 0.011814899 |
| 243 | Ethanone, 1-[4-(1-hydroxy-1-methylethyl)phenyl]- | Ketones | 54549-72-3 | 1.49793415 | 0.75 | 0.011814899 |
| 244 | Ethanone, 1-(3-butyloxiranyl)- | Ketones | 17257-80-6 | 1.476386145 | 0.59 | 0.015473373 |
| 245 | Spiro[2.4]heptan-4-one | Ketones | 5771-32-4 | 1.49793415 | 0.75 | 0.011814899 |
| 246 | Methyl Isobutyl Ketone | Ketones | 108-10-1 | 1.562657703 | 0.69 | 0.003937786 |
| 247 | Cyclohexanone | Ketones | 108-94-1 | 1.543146404 | 132.92 | 0.030830258 |
| 248 | Ethanone, 2-(formyloxy)-1-phenyl- | Ketones | 55153-12-3 | 1.501332034 | 0.38 | 0.011812282 |
| 249 | 5-Undecen-4-one | Ketones | 56312-55-1 | 1.49793415 | 0.75 | 0.011814899 |
| 250 | Cyclohexanone, 4-(1,1-dimethylpropyl)- | Ketones | 16587-71-6 | 1.49793415 | 0.75 | 0.011814899 |
| 251 | 2-Hexanone, 4-hydroxy-5-methyl- | Ketones | 38836-21-4 | 1.49793415 | 0.75 | 0.011814899 |
| 252 | 3-Octanone, 4-methyl- | Ketones | 6137-15-1 | 1.49793415 | 0.75 | 0.011814899 |
| 253 | 2-Heptanone, 4-methyl- | Ketones | 6137-06-0 | 1.38548021 | 0.71 | 0.035383361 |
| 254 | 3-Isopropyl-4-methyl-1-pentyn-3-ol | Ketones | 5333-87-9 | 1.49793415 | 0.75 | 0.011814899 |
| 255 | 3-Pentanone | Ketones | 96-22-0 | 1.49793415 | 0.75 | 0.011814899 |
| 256 | 3,4-Dihydroxyacetophenone | Ketones | 1197-09-7 | 1.49793415 | 0.75 | 0.011814899 |
| 257 | 2-Undecanone | Ketones | 112-12-9 | 1.59754607 | 0.69 | 0.00120564 |
| 258 | 2-Tridecanone | Ketones | 593-08-8 | 1.565875112 | 1.55 | 0.003595667 |
| 259 | 2-Dodecanone | Ketones | 6175-49-1 | 1.627275308 | 54.12 | 0.000166955 |
| 260 | 2-Nonanone | Ketones | 821-55-6 | 1.357804344 | 0.85 | 0.042765305 |
| 261 | 2-Hexanone | Ketones | 591-78-6 | 1.632178396 | 576.03 | 0.003970371 |
| 262 | 2-Decanone | Ketones | 693-54-9 | 1.393788195 | 0.82 | 0.033118894 |
| 263 | 2,7-Octanedione | Ketones | 1626-09-1 | 1.49793415 | 0.75 | 0.011814899 |
| 264 | Ethanone, 1-(2,4-difluorophenyl)- | Ketones | 364-83-0 | 1.49793415 | 0.75 | 0.011814899 |
| 265 | 2,3-Hexanedione | Ketones | 3848-24-6 | 1.490510929 | 0.74 | 0.012813836 |
| 266 | 2,3-Butanedione | Ketones | 431-03-8 | 1.391619158 | 0.78 | 0.033635844 |
| 267 | 2,2-Dimethyl-3-heptanone | Ketones | 19078-97-8 | 1.49793415 | 0.75 | 0.011814899 |
| 268 | Cyclohexanone, 2,2,6-trimethyl- | Ketones | 2408-37-9 | 1.53374931 | 0.82 | 0.006817508 |
| 269 | Cyclobutanone, 2,2,4,4-tetramethyl- | Ketones | 4298-75-3 | 1.49793415 | 0.75 | 0.011814899 |
| 270 | Ethanone, 1-(3-ethylcyclobutyl)- | Ketones | 56335-71-8 | 1.338493728 | 0.71 | 0.048743818 |
| 271 | 2-Pentenoic acid | Lipids and lipid-like molecules | 626-98-2 | 1.49793415 | 0.75 | 0.011814899 |
| 272 | 1,3-Cyclohexadiene, 5-(1,5-dimethyl-4-hexenyl)-2-methyl-, [S-(R*,S*)]- | Lipids and lipid-like molecules | 495-60-3 | 1.496617768 | 0.66 | 0.04705005 |
| 273 | p-Mentha-1,5,8-triene | Lipids and lipid-like molecules | 21195-59-5 | 1.49793415 | 0.75 | 0.011814899 |
| 274 | Copaene | Lipids and lipid-like molecules | 3856-25-5 | 1.592632684 | 0.53 | 0.001529687 |
| 275 | a-Phellandrene | Lipids and lipid-like molecules | 99-83-2 | 1.619058938 | 0.63 | 0.000457775 |
| 276 | 9-Octadecynoic acid | Lipids and lipid-like molecules | 506-24-1 | 1.524770935 | 0.74 | 0.007747093 |
| 277 | 5,9-Undecadien-2-ol, 6,10-dimethyl- | Lipids and lipid-like molecules | 53837-34-6 | 1.531500922 | 0.74 | 0.007057708 |
| 278 | 2-Cyclohexen-1-ol, 3-methyl-6-(1-methylethyl)- | Lipids and lipid-like molecules | 491-04-3 | 1.645751549 | 0 | 1.84321E-08 |
| 279 | 3-Methylene-2-norbornanone | Lipids and lipid-like molecules | 5597-27-3 | 1.355500595 | 14.64 | 0.046646018 |
| 280 | 3-Buten-2-one, 4-(2-hydroxy-2,6,6-trimethylcyclohexyl)- | Lipids and lipid-like molecules | 55955-46-9 | 1.390944176 | 0.89 | 0.034689752 |
| 281 | 2,4,6-Octatriene, 2,6-dimethyl- | Lipids and lipid-like molecules | 673-84-7 | 1.49793415 | 0.75 | 0.011814899 |
| 282 | 1,6,10-Dodecatrien-3-ol, 3,7,11-trimethyl- | Lipids and lipid-like molecules | 7212-44-4 | 1.49793415 | 0.75 | 0.011814899 |
| 283 | 1,4-Methanobenzocyclodecene, 1,2,3,4,4a,5,8,9,12,12a-decahydro- | Lipids and lipid-like molecules | 74708-73-9 | 1.49793415 | 0.75 | 0.011814899 |
| 284 | 1,3,6,10-Dodecatetraene, 3,7,11-trimethyl-, (Z,E)- | Lipids and lipid-like molecules | 26560-14-5 | 1.432455428 | 0.78 | 0.023512798 |
| 285 | (E,E,E)-3,7,11,15-Tetramethylhexadeca-1,3,6,10,14-pentaene | Lipids and lipid-like molecules | 77898-97-6 | 1.49793415 | 0.75 | 0.011814899 |
| 286 | (E,E)-7,11,15-Trimethyl-3-methylene-hexadeca-1,6,10,14-tetraene | Lipids and lipid-like molecules | 70901-63-2 | 1.49793415 | 0.75 | 0.011814899 |
| 287 | (3E,7E)-4,8,12-Trimethyltrideca-1,3,7,11-tetraene | Lipids and lipid-like molecules | 62235-06-7 | 1.49116296 | 0.71 | 0.012776175 |
| 288 | Aromandendrene | Lipids and lipid-like molecules | 489-39-4 | 1.631179667 | 0.43 | 0.000141505 |
| 289 | (E)-beta-Farnesene | Lipids and lipid-like molecules | 18794-84-8 | 1.616578224 | 0.58 | 0.000451728 |
| 290 | beta-Pinene | Lipids and lipid-like molecules | 127-91-3 | 1.49793415 | 0.75 | 0.011814899 |
| 291 | Cyclohexene, 3-(1,5-dimethyl-4-hexenyl)-6-methylene-, [S-(R*,S*)]- | Lipids and lipid-like molecules | 20307-83-9 | 1.64306901 | 3600.68 | 3.89006E-06 |
| 292 | Cyclohexene, 1-methyl-3-(1-methylethyl)- | Lipids and lipid-like molecules | 13828-31-4 | 1.49793415 | 0.75 | 0.011814899 |
| 293 | 6-Octen-1-ol, 7-methyl-3-methylene- | Lipids and lipid-like molecules | 13066-51-8 | 1.508935596 | 0.8 | 0.010246611 |
| 294 | 1,7-Nonadien-4-ol, 4,8-dimethyl- | Lipids and lipid-like molecules | 17920-92-2 | 1.406981949 | 0.65 | 0.031545448 |
| 295 | a-Campholenal | Lipids and lipid-like molecules | 4501-58-0 | 1.645560014 | 659.61 | 6.5356E-08 |
| 296 | Guaiol | Lipids and lipid-like molecules | 489-86-1 | 1.49793415 | 0.75 | 0.011814899 |
| 297 | 2-Cyclohexen-1-one, 3-methyl-6-(1-methylethenyl)- | Lipids and lipid-like molecules | 529-01-1 | 1.49793415 | 0.75 | 0.011814899 |
| 298 | Geranyl bromide | Lipids and lipid-like molecules | 6138-90-5 | 1.482456195 | 0.72 | 0.014530716 |
| 299 | Cyclohexene, 1-methyl-4-(1-methylethylidene)- | Lipids and lipid-like molecules | 586-62-9 | 1.540380236 | 0.79 | 0.006156002 |
| 300 | Naphthalene, 1,6-dimethyl-4-(1-methylethyl)- | Lipids and lipid-like molecules | 483-78-3 | 1.563163964 | 0.72 | 0.003761083 |
| 301 | Hexanamide | Lipids and lipid-like molecules | 628-02-4 | 1.49793415 | 0.75 | 0.011814899 |
| 302 | Enanthamide | Lipids and lipid-like molecules | 628-62-6 | 1.49793415 | 0.75 | 0.011814899 |
| 303 | a-Terpineol | Lipids and lipid-like molecules | 98-55-5 | 1.512263539 | 0.78 | 0.009783487 |
| 304 | Benzene, 1-(1,5-dimethyl-4-hexenyl)-4-methyl- | Lipids and lipid-like molecules | 644-30-4 | 1.568991486 | 0.68 | 0.003088985 |
| 305 | Terpinen-4-ol | Lipids and lipid-like molecules | 562-74-3 | 1.501221276 | 0.81 | 0.011568226 |
| 306 | 1-Dodecanol, 3,7,11-trimethyl- | Lipids and lipid-like molecules | 6750-34-1 | 1.421506908 | 0.75 | 0.02642007 |
| 307 | 2H-2,4a-Ethanonaphthalene, 1,3,4,5,6,7-hexahydro-2,5,5-trimethyl- | Lipids and lipid-like molecules | 32391-44-9 | 1.49793415 | 0.75 | 0.011814899 |
| 308 | Tricyclo[2.2.1.0(2,6)]heptane, 1,3,3-trimethyl- | Lipids and lipid-like molecules | 488-97-1 | 1.405740841 | 0.86 | 0.030522503 |
| 309 | Tricyclo[3.2.1.0(2,4)]octane, 8-methylene-, (1a,2a,4a,5a)- | Lipids and lipid-like molecules | 38310-48-4 | 1.49793415 | 0.75 | 0.011814899 |
| 310 | (+)-cis-Verbenol, acetate | Lipids and lipid-like molecules | 29135-27-1 | 1.645671555 | 2791.54 | 1.62569E-08 |
| 311 | Cyclohexene, 3-methyl-6-(1-methylethyl)- | Lipids and lipid-like molecules | 5256-65-5 | 1.49793415 | 0.75 | 0.011814899 |
| 312 | 3-Cyclohexene-1-acetaldehyde, a,4-dimethyl- | Lipids and lipid-like molecules | 29548-14-9 | 1.382828646 | 0.87 | 0.035554112 |
| 313 | 1,6,10-Dodecatrien-3-ol, 3,7,11-trimethyl-, (E)- | Lipids and lipid-like molecules | 40716-66-3 | 1.52515338 | 0.78 | 0.007836087 |
| 314 | 1H-3a,7-Methanoazulene, 2,3,4,7,8,8a-hexahydro-3,6,8,8-tetramethyl-, [3R-(3a,3abeta,7beta,8aa)]- | Lipids and lipid-like molecules | 469-61-4 | 1.446314972 | 1.41 | 0.02173496 |
| 315 | Cyclopentaneacetaldehyde, 2-formyl-3-methyl-a-methylene- | Lipids and lipid-like molecules | 5951-57-5 | 1.49793415 | 0.75 | 0.011814899 |
| 316 | Camphene | Lipids and lipid-like molecules | 79-92-5 | 1.53672306 | 0.79 | 0.006523245 |
| 317 | 1,6,10,14-Hexadecatetraen-3-ol, 3,7,11,15-tetramethyl-, (E,E)- | Lipids and lipid-like molecules | 1113-21-9 | 1.49793415 | 0.75 | 0.011814899 |
| 318 | Geraniol | Lipids and lipid-like molecules | 106-24-1 | 1.49793415 | 0.75 | 0.011814899 |
| 319 | Linalyl acetate | Lipids and lipid-like molecules | 115-95-7 | 1.637842261 | 131623.05 | 0.002035501 |
| 320 | Myrtenyl methyl ether | Lipids and lipid-like molecules | 202527-57-9 | 1.49793415 | 0.75 | 0.011814899 |
| 321 | Ylangene | Lipids and lipid-like molecules | 14912-44-8 | 1.592965848 | 0.65 | 0.001545628 |
| 322 | 2H-1b,4-Ethanopentaleno[1,2-b]oxirene, hexahydro-, (1aa,1bbeta,4beta,4aa,5aa)- | Lipids and lipid-like molecules | 117221-80-4 | 1.49793415 | 0.75 | 0.011814899 |
| 323 | a-Cubebene | Lipids and lipid-like molecules | 17699-14-8 | 1.433997402 | 0.71 | 0.024082027 |
| 324 | Benzene, 1-(1,5-dimethylhexyl)-4-methyl- | Lipids and lipid-like molecules | 1461-02-5 | 1.60917212 | 0.57 | 0.000709916 |
| 325 | 1,3-Cyclohexadiene-1-methanol, 4-(1-methylethyl)- | Lipids and lipid-like molecules | 1413-55-4 | 1.49793415 | 0.75 | 0.011814899 |
| 326 | Cyclohexanone, 2-(2-nitro-2-propenyl)- | Organic 1,3-dipolar compounds | 78551-08-3 | 1.49793415 | 0.75 | 0.011814899 |
| 327 | Methane, dichloronitro- | Organic 1,3-dipolar compounds | 7119-89-3 | 1.623549709 | 126.2 | 0.005368268 |
| 328 | Methane, nitro- | Organic 1,3-dipolar compounds | 75-52-5 | 1.645473986 | 581.18 | 1.34552E-07 |
| 329 | Acetamide, N,N'-ethylenebis(N-nitro- | Organic 1,3-dipolar compounds | 922-89-4 | 1.49793415 | 0.75 | 0.011814899 |
| 330 | Propane, 1-nitro- | Organic 1,3-dipolar compounds | 108-03-2 | 1.49793415 | 0.75 | 0.011814899 |
| 331 | Bicyclo[3.3.1]nonan-9-one, 1,2,4-trimethyl-3-nitro-, (2-endo,3-exo,4-exo)-(.+-.)- | Organic 1,3-dipolar compounds | 129967-65-3 | 1.448253942 | 0.8 | 0.021760001 |
| 332 | Acetamide | Organic acids and derivatives | 60-35-5 | 1.521146285 | 0.58 | 0.00812488 |
| 333 | Ethyl methanesulfinate | Organic acids and derivatives | 819-75-0 | 1.537159715 | 0.76 | 0.006146511 |
| 334 | 1-Propanesulfinic acid, methyl ester | Organic acids and derivatives | 41892-32-4 | 1.49793415 | 0.75 | 0.011814899 |
| 335 | Acetamide, 2-fluoro- | Organic acids and derivatives | 640-19-7 | 1.49793415 | 0.75 | 0.011814899 |
| 336 | Butane, 1-(ethenyloxy)-3-methyl- | Organic oxygen compounds | 39782-38-2 | 1.49793415 | 0.75 | 0.011814899 |
| 337 | 2-Butenal, 2-methyl-, (E)- | Organic oxygen compounds | 497-03-0 | 1.478480332 | 0.69 | 0.014749862 |
| 338 | 2-Propenal | Organic oxygen compounds | 107-02-8 | 1.644306915 | 0 | 5.80645E-07 |
| 339 | 2,6,6-Trimethylcyclohexa-1,4-dienecarbaldehyde | Organic oxygen compounds | 162376-82-1 | 1.586583324 | 0.78 | 0.002177189 |
| 340 | trans,trans-3,5-Heptadien-2-one | Organic oxygen compounds | 18402-90-9 | 1.49793415 | 0.75 | 0.011814899 |
| 341 | 1-Propene, 1-methoxy-2-methyl- | Organic oxygen compounds | 17574-84-4 | 1.49793415 | 0.75 | 0.011814899 |
| 342 | 1,4-Pentadien-3-one | Organic oxygen compounds | 1890-28-4 | 1.643896384 | 401.29 | 3.02498E-06 |
| 343 | Acetoin | Organic oxygen compounds | 513-86-0 | 1.49793415 | 0.75 | 0.011814899 |
| 344 | Butanal, 2-ethyl-3-methyl- | Organic oxygen compounds | 26254-92-2 | 1.645487704 | 1739.08 | 1.58762E-07 |
| 345 | Hydroxylamine, O-(3-methylbutyl)- | Organic oxygen compounds | 19411-65-5 | 1.49793415 | 0.75 | 0.011814899 |
| 346 | Pentanal, 2-methyl- | Organic oxygen compounds | 123-15-9 | 1.496231568 | 0.75 | 0.011973965 |
| 347 | 3-Hepten-2-one | Organic oxygen compounds | 1119-44-4 | 1.506172597 | 0.78 | 0.010539664 |
| 348 | Ethene, tetramethoxy- | Organic oxygen compounds | 1069-12-1 | 1.642389501 | 0 | 3.39072E-06 |
| 349 | 1,5-Hexadiene, 3,3,4,4-tetrafluoro- | Organohalogen compounds | 1763-21-9 | 1.49793415 | 0.75 | 0.011814899 |
| 350 | 1-Butene, 2-(chloromethyl)- | Organohalogen compounds | 23010-02-8 | 1.49793415 | 0.75 | 0.011814899 |
| 351 | Trifluoromethylthiocyanate | Organohalogen compounds | 690-24-4 | 1.49793415 | 0.75 | 0.011814899 |
| 352 | Trichloromethane | Organohalogen compounds | 67-66-3 | 1.60512248 | 124.69 | 0.011871276 |
| 353 | Hexane, 3,3,4,4-tetrafluoro- | Organohalogen compounds | 648-36-2 | 1.550067996 | 15046.22 | 0.028998181 |
| 354 | Octane, 2-bromo- | Organohalogen compounds | 557-35-7 | 1.466097031 | 0.61 | 0.016964464 |
| 355 | Hexane, 1-chloro- | Organohalogen compounds | 544-10-5 | 1.506468068 | 0.66 | 0.010577903 |
| 356 | Heptane, 1-chloro- | Organohalogen compounds | 629-06-1 | 1.485499929 | 0.69 | 0.01388067 |
| 357 | Methanesulfonyl fluoride | Organohalogen compounds | 558-25-8 | 1.57129001 | 0.61 | 0.002989755 |
| 358 | 7,9-Di-tert-butyl-1-oxaspiro(4,5)deca-6,9-diene-2,8-dione | Organoheterocyclic compounds | 82304-66-3 | 1.554651486 | 2.17 | 0.004471505 |
| 359 | Oxirane, ethenyl- | Organoheterocyclic compounds | 930-22-3 | 1.549523556 | 10682.98 | 0.031062491 |
| 360 | Furan, 2-(2-propenyl)- | Organoheterocyclic compounds | 75135-41-0 | 1.645860871 | 0 | 1.88936E-08 |
| 361 | 2H-Pyran, 2-ethenyltetrahydro-2,6,6-trimethyl- | Organoheterocyclic compounds | 7392-19-0 | 1.455163825 | 0.78 | 0.019667773 |
| 362 | 1-Oxaspiro[4.5]deca-3,6-diene, 2,6,10,10-tetramethyl- | Organoheterocyclic compounds | 54344-61-5 | 1.588601999 | 0.67 | 0.001817702 |
| 363 | (3R,6S)-2,2,6-Trimethyl-6-vinyltetrahydro-2H-pyran-3-ol | Organoheterocyclic compounds | 39028-58-5 | 1.417523873 | 0.8 | 0.027896223 |
| 364 | Pyrazine | Organoheterocyclic compounds | 290-37-9 | 1.634610935 | 0.31 | 5.9601E-05 |
| 365 | Eucalyptol | Organoheterocyclic compounds | 470-82-6 | 1.641653403 | 0 | 0.000794243 |
| 366 | 1,3,5-Triazine-2,4,6(1H,3H,5H)-trione, 1,3,5-trimethyl- | Organoheterocyclic compounds | 827-16-7 | 1.47108186 | 0.62 | 0.016296295 |
| 367 | Isoquinoline | Organoheterocyclic compounds | 119-65-3 | 1.49793415 | 0.75 | 0.011814899 |
| 368 | Oxirane, (2-methylpropyl)- | Organoheterocyclic compounds | 23850-78-4 | 1.49793415 | 0.75 | 0.011814899 |
| 369 | 2H-Pyran-2,6(3H)-dione, dihydro- | Organoheterocyclic compounds | 108-55-4 | 1.49793415 | 0.75 | 0.011814899 |
| 370 | 7-Isoquinolinol, 1,2,3,4-tetrahydro-1-[(4-hydroxyphenyl)methyl]-6-methoxy-, (S)- | Organoheterocyclic compounds | 486-39-5 | 1.49793415 | 0.75 | 0.011814899 |
| 371 | Furfuryl alcohol, tetrahydro-5-methyl-, cis- | Organoheterocyclic compounds | 16015-08-0 | 1.49793415 | 0.75 | 0.011814899 |
| 372 | Dibenzofuran | Organoheterocyclic compounds | 132-64-9 | 1.439448631 | 0.76 | 0.022774525 |
| 373 | 1-Oxaspiro[4.5]dec-6-ene, 2,6,10,10-tetramethyl- | Organoheterocyclic compounds | 36431-72-8 | 1.559597445 | 0.71 | 0.004004367 |
| 374 | Allopurinol | Organoheterocyclic compounds | 315-30-0 | 1.645747971 | 0 | 1.8958E-08 |
| 375 | 2H-Pyran-2-one, tetrahydro-6-propyl- | Organoheterocyclic compounds | 698-76-0 | 1.41578392 | 0.66 | 0.028879182 |
| 376 | 1H-Pyrrole-2,5-dione, 1-ethyl- | Organoheterocyclic compounds | 128-53-0 | 1.34960853 | 0.43 | 0.044866809 |
| 377 | 1H-Pyrrole, 1-ethyl- | Organoheterocyclic compounds | 617-92-5 | 1.529351167 | 0.73 | 0.00745546 |
| 378 | 1,3-Dimethyl-2,4,5-trioxoimidazolidine | Organoheterocyclic compounds | 5176-82-9 | 1.454603809 | 0.55 | 0.019526054 |
| 379 | 6H-Purine-6-thione, 1,7-dihydro-7-methyl- | Organoheterocyclic compounds | 3324-79-6 | 1.49793415 | 0.75 | 0.011814899 |
| 380 | 6-Methyl-6-(5-methylfuran-2-yl)heptan-2-one | Organoheterocyclic compounds | 50464-95-4 | 1.53543544 | 0.69 | 0.006597316 |
| 381 | 2,4-Diamino-6-methyl-1,3,5-triazine | Organoheterocyclic compounds | 542-02-9 | 1.49793415 | 0.75 | 0.011814899 |
| 382 | 5-Isoxazolecarboxylic acid, 4,5-dihydro-5-methyl-, methyl ester, (R)- | Organoheterocyclic compounds | 64018-42-4 | 1.49793415 | 0.75 | 0.011814899 |
| 383 | 1H-Benzimidazole, 5-methoxy- | Organoheterocyclic compounds | 4887-80-3 | 1.49793415 | 0.75 | 0.011814899 |
| 384 | Pyrimidine, 5-methyl- | Organoheterocyclic compounds | 2036-41-1 | 1.49793415 | 0.75 | 0.011814899 |
| 385 | 4-Methoxy-5H-furan-2-one | Organoheterocyclic compounds | 69556-70-3 | 1.49793415 | 0.75 | 0.011814899 |
| 386 | 4-Amino-3-hydroxytetrahydrothiophene 1,1-dioxide | Organoheterocyclic compounds | 55261-00-2 | 1.49793415 | 0.75 | 0.011814899 |
| 387 | 4H-Pyrido[1,2-a]pyrimidin-4-one, 3-ethyl-6-methyl- | Organoheterocyclic compounds | 57773-19-0 | 1.49793415 | 0.75 | 0.011814899 |
| 388 | Cyclopenta[c]pyran-1,3-dione, 4,4a,5,6-tetrahydro-4,7-dimethyl- | Organoheterocyclic compounds | 66407-26-9 | 1.49793415 | 0.75 | 0.011814899 |
| 389 | 2H-1,5-Benzodiazepin-2-one, 1,3,4,5-tetrahydro-4,7,8-trimethyl- | Organoheterocyclic compounds | 65847-12-3 | 1.49793415 | 0.75 | 0.011814899 |
| 390 | 3-Hydroxypyridine monoacetate | Organoheterocyclic compounds | 17747-43-2 | 1.49793415 | 0.75 | 0.011814899 |
| 391 | N-(Piperidin-3-yl)acetamide | Organoheterocyclic compounds | 5810-55-9 | 1.49793415 | 0.75 | 0.011814899 |
| 392 | Pyrrolidine, 3-methyl- | Organoheterocyclic compounds | 34375-89-8 | 1.49793415 | 0.75 | 0.011814899 |
| 393 | Pyridine, 3-methyl- | Organoheterocyclic compounds | 108-99-6 | 1.614366112 | 2.64 | 0.00058131 |
| 394 | 2H-Pyran, 3,4-dihydro-4-methyl- | Organoheterocyclic compounds | 2270-61-3 | 1.49793415 | 0.75 | 0.011814899 |
| 395 | Cinnoline, 3,4-dimethyl- | Organoheterocyclic compounds | 3929-83-7 | 1.642940844 | 4059.16 | 4.25703E-06 |
| 396 | 3,4,5,6-Tetrahydrophthalic anhydride | Organoheterocyclic compounds | 2426-02-0 | 1.49793415 | 0.75 | 0.011814899 |
| 397 | 2-Isopropylimidazole | Organoheterocyclic compounds | 36947-68-9 | 1.367076308 | 0.06 | 0.041570013 |
| 398 | Furan, 2-ethyl- | Organoheterocyclic compounds | 3208-16-0 | 1.583017752 | 0.7 | 0.002169705 |
| 399 | Pyrazine, ethyl- | Organoheterocyclic compounds | 13925-00-3 | 1.639559808 | 0.29 | 2.0462E-05 |
| 400 | 1H-Pyrrole, 2-ethyl- | Organoheterocyclic compounds | 1551-06-0 | 1.644862815 | 0 | 1.28793E-06 |
| 401 | Furan, 2-pentyl- | Organoheterocyclic compounds | 3777-69-3 | 1.457344441 | 0.58 | 0.018521002 |
| 402 | 2-Methoxytetrahydrofuran | Organoheterocyclic compounds | 13436-45-8 | 1.49793415 | 0.75 | 0.011814899 |
| 403 | 3(2H)-Furanone, dihydro-2-methyl- | Organoheterocyclic compounds | 3188-00-9 | 1.645567687 | 767.14 | 7.21344E-08 |
| 404 | 2H-Pyran, tetrahydro-2-methyl- | Organoheterocyclic compounds | 10141-72-7 | 1.49793415 | 0.75 | 0.011814899 |
| 405 | Thiazole, 2-methyl- | Organoheterocyclic compounds | 3581-87-1 | 1.614498796 | 0.51 | 0.000515207 |
| 406 | Furan, 2-methyl- | Organoheterocyclic compounds | 534-22-5 | 1.485894404 | 0.74 | 0.014137816 |
| 407 | Pyrazine, methyl- | Organoheterocyclic compounds | 109-08-0 | 1.631436638 | 0.45 | 0.000118556 |
| 408 | 2-n-Propylaziridine | Organoheterocyclic compounds | 3647-38-9 | 1.49793415 | 0.75 | 0.011814899 |
| 409 | 2-Pyrazoline, 1-isobutyl-3-methyl- | Organoheterocyclic compounds | 26964-53-4 | 1.434389328 | 0.01 | 0.023745203 |
| 410 | Azetidine, 2-phenyl- | Organoheterocyclic compounds | 22610-18-0 | 1.644337541 | 0 | 2.42583E-06 |
| 411 | 5-Acetyl-2-amino-4-methylthiazole | Organoheterocyclic compounds | 30748-47-1 | 1.49793415 | 0.75 | 0.011814899 |
| 412 | 2,4-Diamino-7(8H)-pteridinone | Organoheterocyclic compounds | 26212-13-5 | 1.49793415 | 0.75 | 0.011814899 |
| 413 | 2,2'-Bioxirane | Organoheterocyclic compounds | 1464-53-5 | 1.49793415 | 0.75 | 0.011814899 |
| 414 | 1-Methyl-1H-1,2,4-triazole | Organoheterocyclic compounds | 6086-21-1 | 1.49793415 | 0.75 | 0.011814899 |
| 415 | Oxirane, hexyl- | Organoheterocyclic compounds | 2984-50-1 | 1.49793415 | 0.75 | 0.011814899 |
| 416 | Oxirane, propyl- | Organoheterocyclic compounds | 1003-14-1 | 1.49793415 | 0.75 | 0.011814899 |
| 417 | Oxirane, pentyl- | Organoheterocyclic compounds | 5063-65-0 | 1.49793415 | 0.75 | 0.011814899 |
| 418 | 1,2,5-Trimethylpyrrole | Organoheterocyclic compounds | 930-87-0 | 1.49793415 | 0.75 | 0.011814899 |
| 419 | 1,2,4,5-Tetrazine | Organoheterocyclic compounds | 290-96-0 | 1.49793415 | 0.75 | 0.011814899 |
| 420 | 1,1'-Carbonyldiimidazole | Organoheterocyclic compounds | 530-62-1 | 1.49793415 | 0.75 | 0.011814899 |
| 421 | (S)-(-)-1-Amino-2-(methoxymethyl)-pyrrolidine | Organoheterocyclic compounds | 59983-39-0 | 1.644603881 | 229.72 | 1.22315E-06 |
| 422 | 2H-Pyran, 3,6-dihydro-4-methyl-2-(2-methyl-1-propenyl)- | Organoheterocyclic compounds | 1786-08-9 | 1.458642508 | 0.82 | 0.019076686 |
| 423 | Oxepine, 2,7-dimethyl- | Organoheterocyclic compounds | 1487-99-6 | 1.443622167 | 0.77 | 0.021944459 |
| 424 | Aziridinone, 1,3-bis(1,1-dimethylethyl)- | Organoheterocyclic compounds | 14387-89-4 | 1.49793415 | 0.75 | 0.011814899 |
| 425 | Octanoic acid, ethyl ester | Organoheterocyclic compounds | 106-32-1 | 1.550037172 | 0.7 | 0.004738545 |
| 426 | 2,5,5,8a-Tetramethyl-3,4,4a,5,6,8a-hexahydro-2H-chromene | Organoheterocyclic compounds | 72746-44-2 | 1.377911257 | 0.83 | 0.037402065 |
| 427 | Butanenitrile, 3-methyl- | Heterocyclic_Compounds | 625-28-5 | 1.49793415 | 0.75 | 0.011814899 |
| 428 | Acetaldehyde, propylhydrazone | Heterocyclic_Compounds | 7422-88-0 | 1.49793415 | 0.75 | 0.011814899 |
| 429 | Acetaldehyde, ethylhydrazone | Heterocyclic_Compounds | 20487-02-9 | 1.49793415 | 0.75 | 0.011814899 |
| 430 | Hexanenitrile | Heterocyclic_Compounds | 628-73-9 | 1.511875442 | 0.6 | 0.009508202 |
| 431 | Cyclohexanone, oxime | Heterocyclic_Compounds | 100-64-1 | 1.49793415 | 0.75 | 0.011814899 |
| 432 | Propanal, propylhydrazone | Heterocyclic_Compounds | 19718-39-9 | 1.49793415 | 0.75 | 0.011814899 |
| 433 | Aminoacetonitrile | Heterocyclic_Compounds | 540-61-4 | 1.49793415 | 0.75 | 0.011814899 |
| 434 | 1,2-Ethanediamine, N'-ethyl-N,N-dimethyl- | Heterocyclic_Compounds | 123-83-1 | 1.49793415 | 0.75 | 0.011814899 |
| 435 | Methanamine, N,N-difluoro- | Heterocyclic_Compounds | 753-58-2 | 1.49793415 | 0.75 | 0.011814899 |
| 436 | 1-Propyl-1,2,3,4-tetrahydropyrrolo[1,2-a]pyrazine | Heterocyclic_Compounds | 112758-86-8 | 1.49793415 | 0.75 | 0.011814899 |
| 437 | Thiourea | Organosulfur compounds | 62-56-6 | 1.49793415 | 0.75 | 0.011814899 |
| 438 | Dimethyl Sulfoxide | Organosulfur compounds | 67-68-5 | 1.470382714 | 0.62 | 0.016699096 |
| 439 | Disulfide, dimethyl | Organosulfur compounds | 624-92-0 | 1.638460065 | 22.19 | 3.04222E-05 |
| 440 | 3,5-Dithiahexanol 5,5-dioxide | Organosulfur compounds | 68483-74-9 | 1.49793415 | 0.75 | 0.011814899 |
| 441 | Benzene, 1,1'-(1,2-cyclobutanediyl)bis-, cis- | Phenylpropanoids and polyketides | 7694-30-6 | 1.49793415 | 0.75 | 0.011814899 |
| 442 | 3,5-Dimethoxy-4-hydroxycinnamic acid | Phenylpropanoids and polyketides | 530-59-6 | 1.645579864 | 834.45 | 6.09898E-08 |
| 443 | Benzenamine, 4,4'-(1,2-ethanediyl)bis- | Phenylpropanoids and polyketides | 621-95-4 | 1.49793415 | 0.75 | 0.011814899 |
| 444 | 1,2-Ethanediol, 1,2-diphenyl-, (R*,R*)-(ÃÂÃÂÃÂÃÂ±)- | Phenylpropanoids and polyketides | 655-48-1 | 1.49793415 | 0.75 | 0.011814899 |
| 445 | Ethanone, 2-chloro-1,2-diphenyl- | Phenylpropanoids and polyketides | 447-31-4 | 1.385718837 | 0.24 | 0.034208771 |
| 446 | Cyclopentene, 3-methyl-1-(1-methylethyl)- |  | 51115-02-7 | 1.49793415 | 0.75 | 0.011814899 |
| 447 | Furan, 2-(1-pentenyl)-, (E)- |  | 20992-69-2 | 1.58669025 | 0.66 | 0.001959925 |
| 448 | 6-Methyl-2-(4-methylcyclohex-3-en-1-yl)hepta-1,5-dien-4-ol |  | 38142-56-2 | 1.49793415 | 0.75 | 0.011814899 |
| 449 | 4-Oxohex-2-enal |  | 20697-55-6 | 1.422590825 | 0.79 | 0.026751493 |
| 450 | 3-Methylbut-2-en-1-yl pivalate |  | 211429-71-9 | 1.492068154 | 0.73 | 0.012546139 |
| 451 | 2-Cyclopentene-1-carboxylic acid, 1-methyl-, methyl ester |  | 68317-73-7 | 1.645315148 | 2371.56 | 2.22073E-07 |
| 452 | 1,5-Cyclooctadiene, 1,2-dimethyl- |  | 6588-51-8 | 1.49793415 | 0.75 | 0.011814899 |
| 453 | (S,E)-2,5-Dimethyl-4-vinylhexa-2,5-dien-1-yl acetate |  | 20384-05-8 | 1.49793415 | 0.75 | 0.011814899 |
| 454 | Bicyclo[3.1.0]hexane-6-methanol, 2-hydroxy-1,4,4-trimethyl- |  | 58795-41-8 | 1.49793415 | 0.75 | 0.011814899 |
| 455 | Bicyclo[2.2.1]heptane, 2,2-dimethyl-3-methylene-, (1R)- |  | 5794-03-6 | 1.58003409 | 0.8 | 0.002496322 |
| 456 | Pentadecanal- |  | 2765-11-9 | 1.473321923 | 0.66 | 0.016967849 |
| 457 | Geranyl nitrile |  | 101660-61-1 | 1.49793415 | 0.75 | 0.011814899 |
| 458 | Methanethiol |  | 74-93-1 | 1.539067898 | 0.34 | 0.00607574 |
| 459 | Methyl-6-deoxy-6-fluoro-2,3,4-tri-O-methylbetad-galactopyranoside |  | 2248-90-0 | 1.49793415 | 0.75 | 0.011814899 |
| 460 | Oxirane, 2,2'-(1,4-butanediyl)bis- |  | 2426-07-5 | 1.374304263 | 0 | 0.041065939 |
| 461 | Cyclohexyl isopropylphosphonofluoridate |  | 333416-55-0 | 1.49793415 | 0.75 | 0.011814899 |
| 462 | Cyclopropanecarboxylic acid, cyclohexylmethyl ester |  | 208941-23-5 | 1.49793415 | 0.75 | 0.011814899 |
| 463 | trans-8a-Methylperhydroazulen-4(1H)-one |  | 32166-45-3 | 1.49793415 | 0.75 | 0.011814899 |
| 464 | Butanal, 3,3-dimethyl-2-oxo-, hemihydrate |  | 77572-68-0 | 1.49793415 | 0.75 | 0.011814899 |
| 465 | 4-Imidazolacetic acid, butyl ester |  | 99133-89-8 | 1.49793415 | 0.75 | 0.011814899 |
| 466 | 3-Ethyl-4-methylpentan-1-ol |  | 38514-13-5 | 1.566545339 | 2.45 | 0.003997796 |
| 467 | 3-Methylnon-1-yn-3-ol |  | 5430-01-3 | 1.49793415 | 0.75 | 0.011814899 |
| 468 | 2-Acetoxytetralin |  | 4294536-57-0 | 1.49793415 | 0.75 | 0.011814899 |
| 469 | Propanal, 2-(4-ethoxyphenyl)-2-methyl- |  | 93622-71-0 | 1.49793415 | 0.75 | 0.011814899 |
| 470 | 1,8(2H,5H)-Naphthalenedione, hexahydro-8a-methyl-, cis- |  | 83406-41-1 | 1.349740318 | 0.67 | 0.045428618 |
| 471 | (1-Methoxy-pentyl)-cyclopropane |  | 94883-96-2 | 1.644810835 | 10163.32 | 1.15955E-06 |
| 472 | 2H-Benz[e]inden-3-ol, 3,3a,4,5-tetrahydro-3a-methyl-, (3S-cis)- |  | 71805-91-9 | 1.49793415 | 0.75 | 0.011814899 |
| 473 | (1R,4S,5S)-1,8-Dimethyl-4-(prop-1-en-2-yl)spiro[4.5]dec-7-ene |  | 43219-80-3 | 1.49793415 | 0.75 | 0.011814899 |
| 474 | (3R,4aS,8aS)-8a-Methyl-5-methylene-3-(prop-1-en-2-yl)-1,2,3,4,4a,5,6,8a-octahydronaphthalene |  | 212394-95-1 | 1.417375325 | 0.46 | 0.028253944 |
| 475 | (3S,4aR,8aS)-1,1,3,6-Tetramethyl-3-vinyl-3,4,4a,7,8,8a-hexahydro-1H-isochromene |  | 107602-52-8 | 1.528248984 | 0.76 | 0.007654989 |
| 476 | (3S,4aS,8aR)-1,1,3,6-Tetramethyl-3-vinyl-3,4,4a,7,8,8a-hexahydro-1H-isochromene |  | 107602-53-9 | 1.645705138 | 4711.23 | 1.54824E-08 |
| 477 | (4aS,8R)-4a,8-Dimethyl-4,4a,5,6,7,8-hexahydronaphthalen-2(3H)-one |  | 69460-62-4 | 1.49793415 | 0.75 | 0.011814899 |
| 478 | (E)-4-(But-1-en-1-yl)-1,2-dimethoxybenzene |  | 76252-28-3 | 1.49793415 | 0.75 | 0.011814899 |
| 479 | (R)-3,5,5-Trimethylcyclohex-3-en-1-ol |  | 211107-48-1 | 1.427598807 | 0.83 | 0.024798075 |
| 480 | 1-(7-Hydroxy-1,6,6-trimethyl-10-oxatricyclo[5.2.1.0(2,4)]dec-9-yl)ethanone |  | 90165-01-8 | 1.49793415 | 0.75 | 0.011814899 |
| 481 | 1-Ethyl-3-piperidinamine, N-(2-methylpropionyl)- |  | 1344346-84-4 | 1.49793415 | 0.75 | 0.011814899 |
| 482 | 1,3-Butadiene, 1-(ethylthio)- |  | 10574-85-3 | 1.49793415 | 0.75 | 0.011814899 |
| 483 | 1,5-Cyclooctadien-4-one |  | 1460-21-5 | 1.49793415 | 0.75 | 0.011814899 |
| 484 | 10,11-Epoxycalamenene |  | 143785-42-6 | 1.513072752 | 0.74 | 0.009585703 |
| 485 | 1H-Inden-1-one, 2,3,3a,4,5,7a-hexahydro-4,4,7a-trimethyl- |  | 109629-65-4 | 1.49793415 | 0.75 | 0.011814899 |
| 486 | 1H-Indene, 1-ethylideneoctahydro-7a-methyl-, cis- |  | 56362-87-9 | 1.639914053 | 3331.67 | 0.001676502 |
| 487 | 2-(1-Adamantyl)piperidine |  | 195243-62-0 | 1.49793415 | 0.75 | 0.011814899 |
| 488 | 2-Decene, 9-methyl-, (Z)- |  | 74630-24-3 | 1.49793415 | 0.75 | 0.011814899 |
| 489 | 2-Methylenecyclohexanol |  | 4065-80-9 | 1.49793415 | 0.75 | 0.011814899 |
| 490 | 2-Pentanone, 4-(1,3,3-trimethyl-7-oxabicyclo[4.1.0]hept-2-yl)- |  | 97306-61-1 | 1.449361081 | 0.68 | 0.020915978 |
| 491 | 2-Pentene, 4,4'-oxybis- |  | 52867-34-2 | 1.49793415 | 0.75 | 0.011814899 |
| 492 | 2-tert-Butyltoluene |  | 1074-92-6 | 1.645114006 | 227.78 | 2.34979E-07 |
| 493 | 2(1H)-Pentalenone, 3,3a,4,6a-tetrahydro- |  | 35200-12-5 | 1.600088614 | 790.81 | 0.013195294 |
| 494 | 3-Benzofurancarboxaldehyde, 2-methoxy- |  | 40800-89-3 | 1.49793415 | 0.75 | 0.011814899 |
| 495 | 3-Hexenoic acid, methyl ester, (Z)- |  | 13894-62-7 | 1.49793415 | 0.75 | 0.011814899 |
| 496 | 3-Methyl-2-(2-methyl-2-butenyl)-furan |  | 15186-51-3 | 1.464099717 | 0.77 | 0.01743248 |
| 497 | 3-Methylhexan-1-amine |  | 65530-93-0 | 1.49793415 | 0.75 | 0.011814899 |
| 498 | 3a,6-Methano-3ah-inden-5-ol, octahydro-, (3aa,5a,6a,7abeta)- |  | 16489-23-9 | 1.49793415 | 0.75 | 0.011814899 |
| 499 | 4,4-Dimethyl-1,2,3,4-tetrahydro-gamma-carboline |  | 22315-87-3 | 1.49793415 | 0.75 | 0.011814899 |
| 500 | 4,4-Dimethyl-4,5-dihydro-1,3-oxazol-2-amine, N,N-dimethyl- |  | 23802-98-4 | 1.49793415 | 0.75 | 0.011814899 |
| 501 | 4,4-Ethylenedioxy-pentanenitrile |  | 40159-07-7 | 1.543556115 | 0 | 0.031471464 |
| 502 | 4,8-Dioxatricyclo[5.1.0.0(3,5)]octane, 1-methyl-5-(1-methylethyl)-, (1a,3beta,5beta,7a)- |  | 42569-59-5 | 1.49793415 | 0.75 | 0.011814899 |
| 503 | 4a,8-Dimethyl-2-(prop-1-en-2-yl)-1,2,3,4,4a,5,6,7-octahydronaphthalene |  | 103827-22-1 | 1.624416035 | 0.68 | 0.000283406 |
| 504 | 5,8-Decadien-2-one, 5,9-dimethyl-, (E)- |  | 130876-99-2 | 1.532054179 | 1.19 | 0.006806106 |
| 505 | 6-Propenylbicyclo[3.1.0]hexan-2-one |  | 75283-46-4 | 1.49793415 | 0.75 | 0.011814899 |
| 506 | 6-Thiopyrazolo[3,4-d]pyrimidin-4,6(5H,7H)-dione-3-carboxamide |  | 96555-42-9 | 1.400658651 | 16.21 | 0.030976136 |
| 507 | 8-Oxabicyclo[5.1.0]oct-5-en-2-ol, 1,4,4-trimethyl- |  | 58795-43-0 | 1.49793415 | 0.75 | 0.011814899 |
| 508 | 8a-Methyl-1,2,3,5,8,8a-hexahydronaphthalene |  | 107914-93-2 | 1.49793415 | 0.75 | 0.011814899 |
| 509 | Acetic acid, 1-methylcyclopentyl ester |  | 26600-59-9 | 1.49793415 | 0.75 | 0.011814899 |
| 510 | Antibiotic NFAT 133 |  | 165133-85-7 | 1.49793415 | 0.75 | 0.011814899 |
| 511 | Benzoic acid, 2,5-dihydroxy-, ethyl ester |  | 3943-91-7 | 1.49793415 | 0.75 | 0.011814899 |
| 512 | Bicyclo[3.1.1]hept-3-ene, 2-formylmethyl-4,6,6-trimethyl- |  | 135004-95-4 | 1.49793415 | 0.75 | 0.011814899 |
| 513 | Borane, compd. with dimethylamine (1:1) |  | 74-94-2 | 1.49793415 | 0.75 | 0.011814899 |
| 514 | Butanoic acid, 2-methylcyclohexyl ester, trans- |  | 15287-80-6 | 1.49793415 | 0.75 | 0.011814899 |
| 515 | Caffeine |  | 58-08-2 | 1.452539096 | 0.67 | 0.019173801 |
| 516 | cis-3-Hexenyl salicylate |  | 65405-77-8 | 1.552382432 | 0.66 | 0.004715221 |
| 517 | Cyclohexane, butylidene- |  | 2272-03-9 | 1.49793415 | 0.75 | 0.011814899 |
| 518 | Cyclopentadiene, 1,5,5-trimethyl- |  | 4249-09-6 | 1.638657463 | 0.01 | 0.002044703 |
| 519 | Cyclopentanecarbonitrile |  | 4254-02-8 | 1.518730857 | 0.71 | 0.008667125 |
| 520 | Cyclopropanemethanol, a,2-dimethyl-2-(4-methyl-3-pentenyl)-, [1a(R*),2a]- |  | 121959-70-4 | 1.49793415 | 0.75 | 0.011814899 |
| 521 | Decanoic acid, cyclohexyl ester |  | 1551-40-2 | 1.49793415 | 0.75 | 0.011814899 |
| 522 | Di-epi-a-cedrene-(I) |  | 21996-77-0 | 1.644054804 | 271.39 | 2.04006E-06 |
| 523 | Ditrifluoromethyl(trifluorocarbonyloxy)amine |  | 15496-02-3 | 1.49793415 | 0.75 | 0.011814899 |
| 524 | Ethanamine, 2,2'-azobis[N,N,1,1,2,2-hexafluoro- |  | 105882-89-1 | 1.49793415 | 0.75 | 0.011814899 |
| 525 | Ethanone, 1-(2,3-dihydro-2,3-dimethoxy-2-benzofuranyl)-, trans- |  | 42178-44-9 | 1.643853857 | 58.36 | 2.92235E-06 |
| 526 | Methyl 7-(2-furyl)heptanoate |  | 98188-02-4 | 1.639443274 | 0 | 0.001699081 |
| 527 | Myrtenyl angelate |  | 138530-45-7 | 1.49793415 | 0.75 | 0.011814899 |
| 528 | N-Benzyloxy-2-carbomethoxyaziridine |  | 53084-33-6 | 1.49793415 | 0.75 | 0.011814899 |
| 529 | N-Ethyl-2-isopropoxycarbonylazetidine |  | 54773-06-7 | 1.49793415 | 0.75 | 0.011814899 |
| 530 | N-Methylcoclaurine |  | 3423-07-2 | 1.49793415 | 0.75 | 0.011814899 |
| 531 | Naphthalene, 6-methoxy-2-(1-buten-3-yl)- |  | 101327-54-2 | 1.645744565 | 0 | 1.2404E-08 |
| 532 | Oxamide, N-(3-methoxypropyl)-N'-cycloheptylidenamino- |  | 333441-36-4 | 1.49793415 | 0.75 | 0.011814899 |
| 533 | Oxetane, 2-methyl-4-propyl- |  | 7045-79-6 | 1.644068092 | 154.29 | 2.56586E-06 |
| 534 | Oxirane, 3-[5-(4-azido-2-nitrophenoxy)-3-methyl-3-pentenyl]-2,2-dimethyl-, (E)-ÃÂÃÂÃÂÃÂ±- |  | 106929-47-9 | 1.49793415 | 0.75 | 0.011814899 |
| 535 | Pentadecanoic acid, methyl ester |  | 7132-64-1 | 1.375399314 | 0.79 | 0.037762561 |
| 536 | Pethylbrene |  | 442900-37-0 | 1.645530422 | 511.61 | 5.94131E-05 |
| 537 | Phenol, 5-ethenyl-2-methoxy- |  | 621-58-9 | 1.49793415 | 0.75 | 0.011814899 |

# Table S3 ROAV values at different processing stages

| NO. | Name | Class | OdorCharacter | RangeofOdorMin | Source | ROAV | | | | | | | | | | | | |
| --- | --- | --- | --- | --- | --- | --- | --- | --- | --- | --- | --- | --- | --- | --- | --- | --- | --- | --- |
|  |  |  |  |  |  | X1 | W1 | R15 | R1 | F25 | F1 | M1 | GZ1 | HR1 | HF25 | HF1 | HM1 | HGZ1 |
| 1 | Butanal, 2-methyl- | Aldehydes | Cocoa, Almond | 0.001 | FlavorDB | 24.60502225 | 66.69714945 | 99.77958494 | 86.14801853 | 82.65413443 | 91.05916565 | 100 | 100 | 66.66686265 | 58.49677201 | 47.20668258 | 66.66683732 | 100 |
| 2 | 2-Nonenal, (E)- | Aldehydes | Fatty, Cucumber | 0.0002 | FlavorDB | 100 | 96.25512698 | 79.95609498 | 70.66322651 | 96.14434009 | 100 | 47.54002681 | 55.50209514 | 88.79437276 | 100 | 100 | 92.29065725 | 33.03864309 |
| 3 | Furan, 2-pentyl- | Organoheterocyclic compounds | Green Beans, Vegetable | 0.006 | FlavorDB | 25.98618814 | 13.48465569 | 9.698720577 | 11.67073963 | 14.93423593 | 15.17509367 | 9.09262309 | 8.985396143 | 14.31915728 | 21.01954996 | 19.37533016 | 14.8671957 | 6.77883206 |
| 4 | 2,3-Butanedione | Ketones | pleasant, buttery | 0.002 | FlavorDB | 1.840841246 | 4.186377457 | 3.930376098 | 4.258898293 | 4.674061061 | 4.869233111 | 7.699120781 | 7.02012467 | 6.191072693 | 5.900388905 | 4.771327258 | 14.91219584 | 7.066135223 |
| 5 | Heptanal | Aldehydes | Citrus, Fatty, Rancid | 0.003 | FlavorDB | 20.5830636 | 10.49371452 | 15.02563162 | 14.02392707 | 13.4873341 | 16.4119423 | 9.827329549 | 6.206517444 | 16.08081031 | 15.33469963 | 13.070516 | 20.09233669 | 8.295874587 |
| 6 | 2-Octenal, (E)- | Aldehydes | Nuts, Green, Fatty | 0.003 | FlavorDB | 16.71638283 | 8.377778418 | 9.858108087 | 8.678233152 | 9.743130646 | 11.55359861 | 3.320837236 | 2.396342704 | 10.99149267 | 10.99419921 | 9.486874875 | 6.619607329 | 2.749859003 |
| 7 | Dimethyl sulfide | Ethers | disagreeable,asparagus, putrid | 0.12 | FlavorDB | 0.49179598 | 0.519261383 | 0.894894152 | 0.678170148 | 0.584306353 | 0.63825917 | 0.893559127 | 1.076734497 | 0.788015223 | 0.665335626 | 0.490240195 | 2.080193453 | 1.010563251 |
| 8 | 2-Dodecenal, (E)- | Aldehydes | Green, Fatty, Sweet | 0.00053 | FlavorDB | 6.007626731 | 1.626303989 | 0.001203653 | 2.391382409 | 1.571443992 | 1.330631818 | 0.318503702 | 0.752021264 | 1.046528952 | 2.514060362 | 0.000861629 | 0.706541026 | 0.661758202 |
| 9 | 1-Octen-3-one | Ketones | Mushroom-Like | 0.005 | FlavorDB | 2.727270824 | 1.127142557 | 2.021497367 | 1.684768369 | 2.055272286 | 1.108247964 | 0.821401435 | 0.447040339 | 2.275728219 | 2.111558182 | 1.396012458 | 0.991227593 | 0.72251851 |
| 10 | 2-Undecanone | Ketones | Orange, Fresh, Green | 0.004355 | FlavorDB | 0.344798486 | 0.238584256 | 0.439288352 | 0.310350762 | 0.412726779 | 0.600849577 | 0.447912686 | 0.345234064 | 0.354191589 | 0.41317306 | 0.404215709 | 0.808810255 | 0.308051563 |
| 11 | Propanal, 2-methyl- | Aldehydes | pungent | 0.34 | FlavorDB | 0.270506817 | 0.164947966 | 0.426924903 | 0.149148165 | 0.233484714 | 0.302479159 | 0.285150304 | 0.272695896 | 0.275457401 | 0.239730905 | 0.149822535 | 0.445417114 | 0.196383084 |
| 12 | Pentanal | Aldehydes | sickening, rancid,decayed | 0.4 | FlavorDB | 0.193883625 | 0.051758594 | 0.025748818 | 0.078628299 | 0.100264216 | 0.105156865 | 0.071280434 | 0.063757557 | 0.121046988 | 0.105469307 | 0.100652199 | 0.136187711 | 0.066461536 |
| 13 | a-Terpineol | Lipids and lipid-like molecules | Piney, Iris, Teil | 1 | FlavorDB | 0.269112608 | 0.115551782 | 0.098062287 | 0.139591174 | 0.137712811 | 0.138872356 | 0.060814567 | 0.055741439 | 0.162573503 | 0.131857256 | 0.128914201 | 0.112277105 | 0.055995265 |
| 14 | Limonene | Lipids and lipid-like molecules | lemon, plastic, citrus,rubber, terpeny | 1.8 | FlavorDB | 0.2098171 | 0.04968933 | 0.21563253 | 0.153178419 | 0.147434098 | 0.066617364 | 0.01597106 | 0.055530263 | 0.166033112 | 0.066570869 | 0.088740327 | 0.037835576 | 0.039917826 |
| 15 | Acetaldehyde | Aldehydes | pungent, fruity, suffocating, fresh, green | 1.5 | FlavorDB | 0.24525179 | 0.055697992 | 0.102545168 | 0.103637026 | 0.073612782 | 0.067660132 | 0.044226548 | 0.047667477 | 0.108235797 | 0.129922321 | 0.035132336 | 0.095989727 | 0.0352592 |
| 16 | 1-Hexanol | Alcohols | green grass, plastic | 2.4 | FlavorDB | 0.120099338 | 0.086341724 | 0.175216779 | 0.181483461 | 0.127607481 | 0.07984038 | 0.022000185 | 0.036761671 | 0.201351984 | 0.100348346 | 0.081792837 | 0.087836186 | 0.038309157 |
| 17 | Butanal | Aldehydes | pungent | 0.3 | FlavorDB | 0.289649072 | 0.116248944 | 0.128726264 | 0.109903515 | 0.115181581 | 0.11523765 | 0.047635492 | 0.032482351 | 0.164724851 | 0.115033122 | 0.098598995 | 0.080950739 | 0.031548325 |
| 18 | Furan, 2-ethyl- | Organoheterocyclic compounds | Burnt,Sweet,Coffee-Like | 2.3 | FlavorDB | 0.114900407 | 0.047124205 | 0.051489131 | 0.039243095 | 0.041559862 | 0.040429264 | 0.020597157 | 0.018698779 | 0.049222849 | 0.045517791 | 0.036212765 | 0.035487014 | 0.016979297 |
| 19 | 2-Buten-1-one, 1-(2,6,6-trimethyl-1,3-cyclohexadien-1-yl)- | Ketones | Peach Jam, Sweet | 0.1 | FlavorDB | 1.08771E-05 | 0.026899382 | 0.049693577 | 0.060669389 | 0.138452608 | 5.14533E-06 | 0.047240355 | 0.016839797 | 0.153767772 | 0.061730371 | 0.095641164 | 0.075643134 | 0.05556794 |
| 20 | 1-Octanol | Alcohols | penetrating | 0.9 | FlavorDB | 0.19741521 | 0.090951926 | 0.132392064 | 0.083507417 | 0.070595205 | 0.073339062 | 0.022582922 | 0.01557487 | 0.08152585 | 0.072533797 | 0.064064561 | 0.036246957 | 0.017071991 |

# Table S4 Electronic nose aroma response values

| Serial number | Sensor name | Performance description | Response value | | p | VIP |
| --- | --- | --- | --- | --- | --- | --- |
|  |  |  | GZ1 | HGZ1 |  |  |
| S1 | W1C | Sensitive to aromatic components and benzenoids | 0.57±0.01 | 0.7±0.05 | <0.001 | 1.02 |
| S2 | W5S | Highly sensitive to nitrogen oxides | 21.18±3.27 | 8.17±1.26 | <0.001 | 1.08 |
| S3 | W3C | Sensitive to aromatic components and amines | 0.66±0.001 | 0.74±0.06 | 0.002 | 0.85 |
| S4 | W6S | Sensitive to hydrides | 1.21±0.01 | 1.18±0.04 | 0.067 | 0.51 |
| S5 | W5C | Sensitive to short-chain alkanes and aromatic components | 0.71±0.01 | 0.8±0.02 | 0.078 | 1.11 |
| S6 | W1S | Sensitive to methyl-containing compounds | 2.18±0.06 | 1.74±0.18 | <0.001 | 1 |
| S7 | W1W | Sensitive to sulfur compounds | 30.38±1.5 | 19.03±2.15 | 0.162 | 1.1 |
| S8 | W2S | Sensitive to alcohols, aldehydes, and ketones | 1.75±0.05 | 1.44±0.08 | <0.001 | 1.06 |
| S9 | W2W | Sensitive to aromatic components and organosulfur compounds | 4.75±0.11 | 3.64±0.29 | <0.001 | 1.08 |
| S10 | W3S | Sensitive to long-chain alkanes | 1.59±0.03 | 1.46±0.04 | 0.145 | 1.03 |
